# Comparative skull anatomy of terrestrial and crevice-dwelling *Trachylepis* skinks (Squamata: Scincidae) with a survey of resources in scincid cranial osteology

**DOI:** 10.1371/journal.pone.0184414

**Published:** 2017-09-13

**Authors:** Daniel J. Paluh, Aaron M. Bauer

**Affiliations:** Department of Biology, Villanova University, Villanova, Pennsylvania, United States of America; Institute of Vertebrate Paleontology and Paleoanthropology Chinese Academy of Sciences, CHINA

## Abstract

Skinks account for more than 25% of all lizard species; however, representatives of fewer than a quarter of all species have been characterized osteologically. All but a few of the available cranial descriptions concentrate solely on characters that can be seen externally on the intact skull. Mabuyid skinks of the genus *Trachylepis* are the dominant, fully limbed skinks in Sub-Saharan Africa, and nearly all species have the same generalized body plan. Although a few rock crevice-dwelling species possess slight body depression, extreme dorsoventral depression is observed only in *Trachylepis laevis*. We investigated the detailed skull anatomy of three *Trachylepis* skinks (*T*. *laevis*, *T*. *sulcata*, and *T*. *gonwouoi*, a recently described species allied to *T*. *affinis*) using high-resolution X-ray micro-computed tomography. Our goals were to review the scincid cranial osteology literature in a phylogenetic context, provide a detailed anatomical atlas for the mabuyid lineage, and investigate the morphological adaptations of the highly modified *T*. *laevis*. Our results demonstrate that there is significant morphological variation between these three taxa, including the loss and fusion of structures, as well as changes in the shape, scale, and relationship between individual elements. *Trachylepis laevis* possesses several osteological modifications that have produced a reducton in head depth that are likely functional consequences of extreme rupicolous habits, including a flat skull roof, many strongly recumbent elements, and a depressed neurocranium.We hypothesize these modifications may correspond to descreased bite force and increased capabilities of cranial kinesis. Our study is the first element-by-element description of a skink using computed tomography technology.

## Introduction

The lepidosaurian skull has been studied extensively by systematists and comparative anatomists to understand the evolutionary relationships, anatomy, and functional morphology of tuatara, lizards, and snakes [1–5]. Except for small or monotypic clades (i.e., Sphenodontidae [6], Shinisauridae, Helodermatidae, Lanthanotidae [7–9]), our understanding of the diversity of skull morphology within most extant squamate lineages is limited. The most speciose groups predictably have the smallest percentage of taxa that have been osteologically examined. Skinks (Family Scincidae Oppel, 1811) are one of the most diverse squamate clades, comprising more than 1,600 species and accounting for more than 25% of all lizards (as of March 2017, [10]); however, relatively few scincid cranial anatomy descriptions exist. The crania of ~250 species (16% of all skinks) have been examined and depicted in primary literature as either fully articulated skulls or disarticulated elements, usually focused on a subset of bones of interest (S1 Appendix).

**Fig 1:**
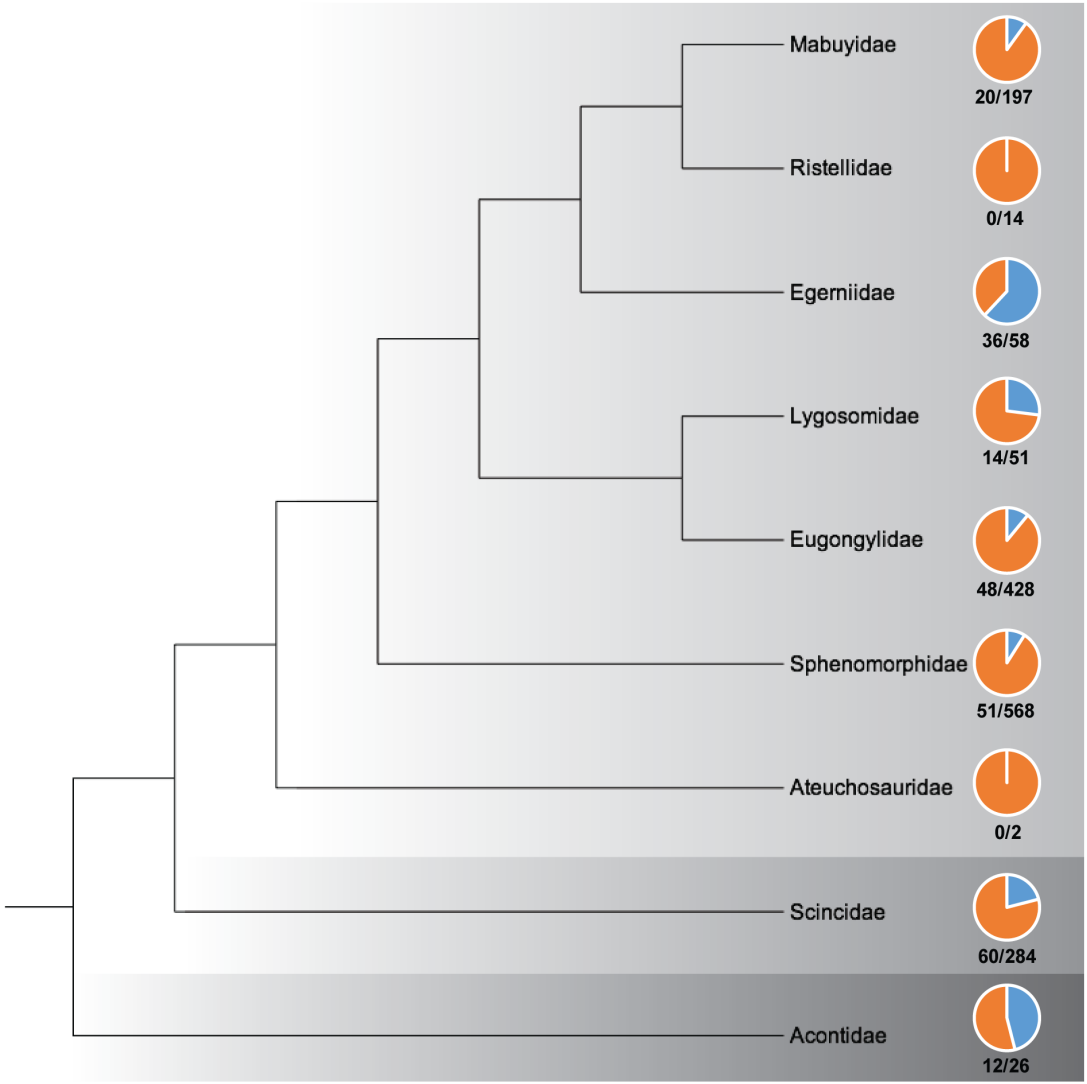
Familial level relationships of skinks based on Hedges [11] and percentage of species osteologically examined within each lineage. Light gray = Lygosomoidea; Medium gray = Scincoidea; Dark gray = Acontoidea. The piecharts illustrate the relative percentage of species that have been cranially described within each family (see S1 Appendix). No skull anatomy descriptions currently exist for ristellids or ateuchosaurids. The ratio below each piechart represents the number of species osteologically examined out of the total number of species within each family (total numbers [10]).

Hedges [11] recently proposed a taxonomic revision of skinks, elevating the three subfamilies within Scincidae to superfamilies (Acontoidea, Scincoidea, Lygosomoidea) and recognizing nine families: Acontidae, Scincidae, Aeuchosauridae, Egerniidae, Eugongylidae, Lygosomidae, Mabuyidae, Ristellidae, and Sphenomorphidae. Although aspects of this taxonomy are potentially contentious [12–14], we utilize it to review the scincid skull anatomy literature in a phylogenetic context to illustrate which regions of the skink tree of life have relatively robust and poor morphological sampling (Fig 1).

The earliest known comparative work on scincid cranial osteology was by Siebenrock [15], in which he described and illustrated seven taxa that currently represent four families (Lygosomidae, Mabuyidae, Scincidae, Sphenomorphidae; S1 Appendix); although the earliest known figured scincid skull was of the extinct giant skink *Chioninia coctei* by Cuvier [16]. Two reoccurring trends in scincid skull anatomy literature of the twentieth and twenty-first century include the investigation of ontogeny and development [17–24; S1 Appendix] and cranial biomechanics and function [25–28; S1 Appendix]. Scincid developmental studies have been facilitated by the relatively high clutch sizes of most skinks and their nearly cosmopolitan distribution, whereas the biomechanical studies have focused on large and easily instrumented taxa, such as the heavily-armored *Tiliqua rugosa*. Greer [29–41] studied the osteology, taxonomy, and evolution of skinks extensively. He proposed cranial diagnostic characters for the three major lineages of skinks (reconized by Hedges [11] as superfamilies): 1) Acontoidea: a divided frontal bone, palatine in broad contact with ectopterygoid, medial separation of the palatine bones in the secondary palate, and curvilinear contact between the ventrolateral ridge of the frontal or its ventral process and the prefrontal; 2) Scincoidea: a divided frontal bone, palatine widely separated from ectopterygoid, medial separation of the palatine bones in the secondary palate, curvilinear contact between the ventrolateral ridge of the frontal or its ventral process and the prefrontal; and 3) Lygosomoidea: a single (fused) frontal bone, palatine separated or in partial contact with ectopterygoid, medial apposition of the palatine bones in the secondary palate, and angular contact between the ventrolateral ridge of the frontal or its ventral process and the prefrontal [29, 38].

The scincid species that have been most often anatomically described are generally common, large taxa that occur in regions with a long history of morphological study, such as the scincid genus *Plestiodon* of North America and Japan, the egerniid skinks of Australia and Melanesia, and the limbless acontids of southern Africa (S1 Appendix, Fig 1). Acontidae is the only scincid family in which all genera have been osteologically described. Five lygosomoid families have been cranially examined, but the skull anatomy of ristellid and ateuchosaurid skinks is completely unknown. Of the lygosomoid families that have been anatomically described, Mabuyidae, Eugongylidae, and Sphenomorphidae have been poorly sampled considering overall diversity of these lineages (10%, 11%, and 9% respectively, Fig 1). Some of the most widespread and typical fully-limbed skinks are members of these understudied clades. Many skink cranial descriptions are limited in detail, and there is a preponderance of palatal images and fully articulated skulls in ventral view, likely due to the phylogenetically informative characters of the palate proposed by Greer [29, 38; S1 Appendix]. There is a scarcity of labeled images (S1 Appendix), particularly for the individual elements of the basicranium, and nearly all labels are of bone names rather than parts or characters of individual elements. The written descriptions vary in detail, but are generally non-comprehensive. Very few studies have described and illustrated every individual cranial element of a skink skull in detail and in isolation (but see [42]; S1 Appendix).

Within Mabuyidae, there are currently 197 species (12% of all skinks). These taxa are diurnal, terrestrial, and widespread where they occur in Africa, Asia, and South America. Few mabuyid species have been osteologically examined, and published cranial descriptions exist for only eight of the 22 genera (*Chioninia*, *Dasia*, *Eumecia*, *Eutropis*, *Hermites*, *Mabuya*, *Psychosaura*, and *Trachylepis*; S1 Appendix). The most speciose mabuyid genus is *Trachylepis*; however, skull anatomy descriptions exist for only six of the ~80 described species (*T*. *atlantica*, articulated skull [43], *T*. *brevicollis*, middle ear and adjacent bones [27], T. *capensis*, articulated skull and subset of disarticulated elements [20], *T*. *maculilabris*, articulated skull [44], *T*. *megalura*, histological sections [45]. and *T*. *polytropis*, palatal bones [33, 46]). Skinner [20] provided the most comprehensive cranial osteology of a skink in the Mabuyidae, *Mabuya* (now *Trachylepis*) *capensis*. Much of this monographic work is devoted to the development of the chondrocranium, however it does also present the descriptive anatomy of the fully-formed skull of neonates, juveniles and adults. The text descriptions of elements are detailed and dorsal, ventral, and lateral views of the entire skull are provided, but only nine fully-formed elements are shown in isolation. This work used reconstructions from histological sections to interpret the morphology.

The members of *Trachylepis* are the dominant, fully limbed skinks of Sub-Saharan Africa. All species within this lineage have the same general body form; however, they inhabitat a variety of environments and exhibit rupicolous, terrestrial, and arboreal habits. Rock crevice-dwelling species can possess flattened bodies compared to fully terrestrial forms (i.e., *Trachylepis sulcata*), but the most phenotypically distinct member of the group is *T*. *laevis*, which is highly dorsoventrally depressed and so morphologically distinct that it was formerly placed in its own monotypic genus [*Oelofsia*, 47]. We here describe the detailed skull anatomy of three *Trachylepis* species (*T*. *laevis*, *T*. *sulcata*, and *T*. *gonwouoi*) and describe every individual cranial element in isolation using high-resolution X-ray micro-computed tomography. Our goals are to provide a detailed anatomical atlas that can act as a reference point for any future studies of mabuyid skinks (and others scincids) and investigate the morphological adaptations of the highly modified *Trachylepis laevis*.

## Materials and methods

The head of an adult male specimen of *Trachylepis laevis* (California Academy of Sciences [CAS] 254838; 67.8 mm SVL), collected from Iona National Park, north of Tambor, Namibe Province, Angola (15° 59' 47.1" S, 12° 24' 25.6" E, 314 m elevation), was computed tomography (CT) scanned at a voxel size (volumetric pixel) of 18.0 μm, the head of an adult male *Trachylepis sulcata* (MCZA-28066 to be accessioned at the National Museum of Namibia; 65.6 mm SVL), collected from Farm Kromhoek Wes, north of Klein Aub, Hardap Region, Namibia (23° 37' 12.8'' S, 16° 42' 25.9'' E), was scanned at 18.8 μm, and the head of an adult male *Trachylepis gonwouoi* (Brigham Young University Bean Museum [BYU] 573530; 79 mm SVL), collected from Nyasoso Village, Mt. Kupe, Southwest Region, Cameroon (4.82312°N, 9.67045°E, 752 m elevation), was scanned at 17 μm. A Zeiss Xradia VersaXRM-520 MicroCT scanner (Pleasanton, CA, USA) was used for all specimens at the Imaging Facility at Cornell University, Institute of Biotechnology, with an X-ray source set to 140 kV, 72 uA, 9.98 W, and 1.0 s acquisition time. The 16bit image stack of each scan consists of 1004 slices.

Three-dimensional reconstructions were generated using Avizo^®^ 9.0.1 (VSG, Visualization Sciences Group, Burlington, Massachusetts, USA). Every individual bone of the skull, as well as an endocast of the inner ear, were digitally segmented. Lengths and angles of structures were measured using the measure tool in Avizo (S1 Fig). To verify the presence or absence of sutures that may be obscured in smoothed segmentations, we examined the 2-dimensional tomogram slices (S2–S4 Figs). To facilitate visualization, individual elements of the fully articulated skull were color coded using a 21-banded rainbow scheme [21-Color Salute, 48], developed with the RColorBrewer package for R. Three-dimensional stereolithography (STL) shape data and tiff stacks of the three specimens are available to view and download from Duke University’s morphological data archive (http://morphosource.com/Detail/ProjectDetail/Show/project_id/370). Identifications of anatomical structures were based on *Trachylepis capensis* as a general reference, as the developmental anatomy and skull osteology of this mabuyid species has been previously described [20]. Interpretations of identity were informed by broad-scale comparisons with a diversity of squamate anatomy descriptions, and homologous designations follow the terminologies of Evans [4], Conrad [9], Gauthier et al. [5], Gelnaw [42], Daza et al. [49–51], Oelrich [52], and Gamble et al. [53].

## Results

### Overall skull morphology

The skull of *Trachylepis* has a length that is roughly 1.5 times greater than its width. The maximum skull lengths (measured from the most anterior point of the dentary to the most posterior point of the retroarticular process) and maximum skull widths (measured from the lateral extent of quadrate to quadrate), respectively, are 14.76 mm (22% SVL) and 8.42 mm for *Trachylepis laevis*, 14.62 mm (22% SVL) and 9.53 mm for *Trachylepis sulcata*, and 17.14 mm (22% SVL) and 10.41 mm for *Trachylepis gonwouoi*. In lateral view, the skull is wedge-shaped, and the angle of snout is roughly 35° in *T*. *laevis*, 50° in *T*. *sulcata*, and 60° in *T*. *gonwouoi*.

**Fig 2:**
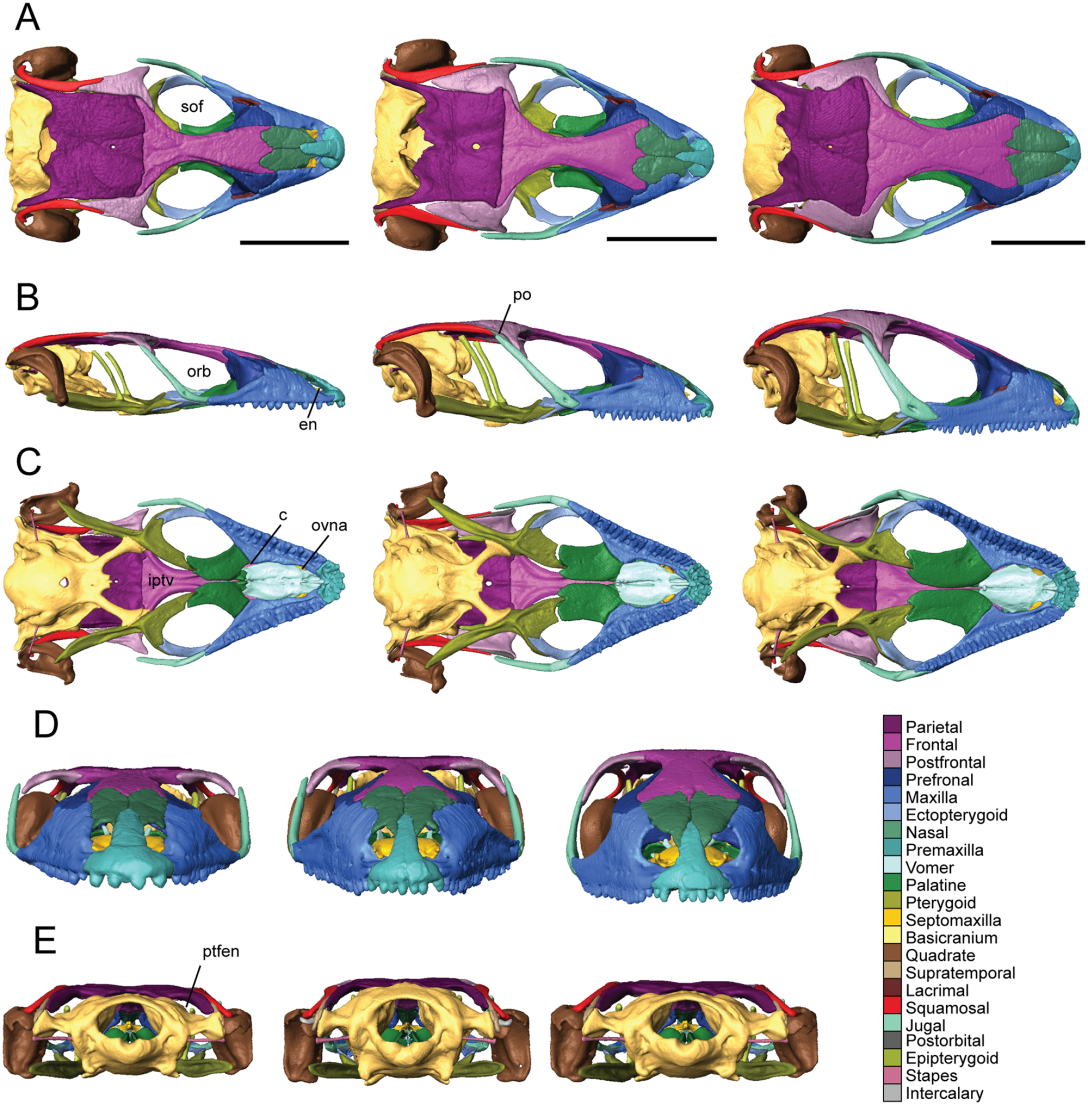
Articulated skulls of *Trachylepis laevis* (left; CAS 254838), *T*. *sulcata* (center; MCZA-28066) and *T*. *gonwouoi* (right; BYU 573530). **A**, dorsal; **B**, lateral; **C**, ventral; **D**, anterior; **E**, posterior views. **Abbreviations**: **c**, choanae; **en**, external nares; **iptv**, interpterygoid vacuity; **orb**, orbit; **ovna**, opening for the vomeronasal apparatus; **po**, postorbital; **ptfen**, posttemporal fenestra; **sof**, suborbital fenestra. Scale bars = 5 mm.

The supratemporal fenestrae are nearly absent in *T*. *sulcata* and *T*. *gonwouoi* and entirely absent in *T*. *laevis* due to enlarged postfrontals, but the postorbital bar is prominent. Osteoderms are present and well-ossified in all three taxa, however palpebral bones are absent. The neurocranium is exposed in dorsal view behind the parietal (Fig 2A). The skull roof (parietal + frontal) is flat in *T*. *laevis* and *T*. *sulcata*, while it is domed in *T*. *gonwouoi* (Fig 2B). The frontoparietal suture is slightly U-shaped and very wide in *T*. *gonwouoi* compared to the narrow suture in *T*. *laevis*. The oval external nares (en, Fig 2B) are visible and placed anteriorly in the rostrum; they are separated by the nasal process of the premaxilla and bordered posteriorly by the nasal and posterolaterally by the maxilla. The oval-shaped orbits (orb, Fig 2B) of *Trachylepis* occupy about 30% of the skull length, are surrounded by bone, and delimited by the prefrontal anteriorly, frontal medially, jugal laterally, and maxilla anteroventrally. The suborbital fenestra is roughly oval-shaped (sof, Fig 2A) and surrounded by the ectopterygoid and maxilla laterally, pterygoid flange and palatine process of the pterygoid posteriorly, and palatine anteromedially. Ventrally, the choanae (c, Fig 2C) are bordered posteriorly by the palatines and anteriorly by the vomer and maxillae, and the openings for the vomeronasal apparatus (ovna, Fig 2C) are bordered posteriorly by the vomer and anteriorly by the maxillae. The interpterygoid vacuity (iptv, Fig 2C) is widest at its center and becomes narrow anteriorly as it approaches the palatines and posteriorly as it approaches the basipterygoid processes. The jugal forms the postorbital bar and lies over the maxilla anteriorly. The lacrimal foramen is bordered by the prefrontal medially and the lacrimal laterally, with no participation of the maxilla or jugal. The fenestra ovalis lies posterior to the quadrate. The roof of the neurocranium (supraoccipital) lies at the same level as that of the dermatocranium (parietal). The posttemporal fenestrae, which are the bilateral posterior spaces between the parietal and braincase, are fully open in all three species (ptfen, Fig 2E).

**Fig 3:**
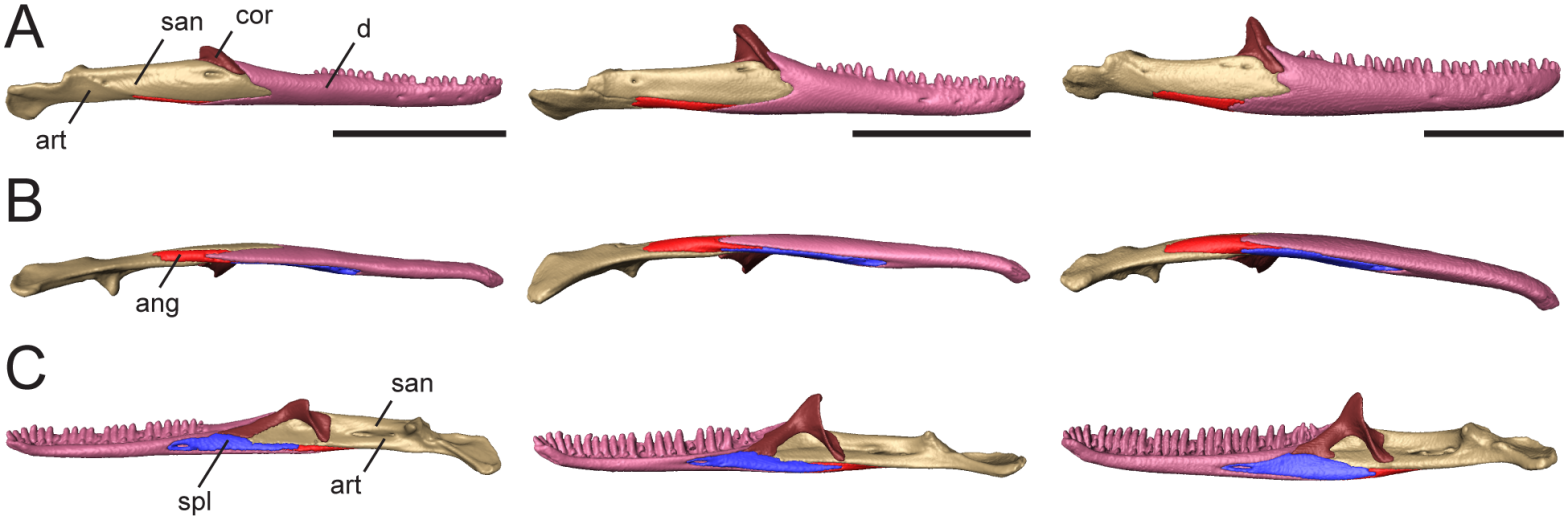
Articulated mandibles of *Trachylepis laevis* (left; CAS 254838), *T*. *sulcata* (center; MCZA-28066) and *T*. *gonwouoi* (right; BYU 573530). **A**, lateral; **B**, ventral; **C**, labial views. **Abbreviations**: **ang**, angular; **art**, articular; **cor**, coronoid; **d**, dentary; **spl**, splenial; **sur**, surangular. Scale bars = 5 mm.

The mandible comprises four discrete elements: the angular (ang), coronoid (cor), dentary (d), and splenial (spl), and two partially fused elements: the articular (art; articular+preacrticular [4]) and surangular (san). The dentary is curved inward anteriorly (Fig 3B) and is the longest bone of the lower jaw. Dentary teeth occupy about one-third of the total jaw length in *T*. *laevis*, one-half in *T*. *sulcata*, and two-thirds in *T*. *gonwouoi* (Fig 3C). In lateral view, the coronoid process is visible, although it is greatly reduced in *T*. *laevis*. The ventral margin of the lower jaw is arced in *T*. *sulcata* and *T*. *gonwouoi*, while it is level in *T*. *laevis* (Fig 3A). The meckelian canal is enclosed by the dentary and splenial, forming a closed tube that continues from the dentary to the mandibular fossa in all three taxa, a synapomorphy for mabuyid skinks [54]. The mandibular fossa is bordered by the articular and surangular. The degree of fusion between the surangular and articular is variable between the three specimens, with nearly complete separation in *T*. *sulcata* and complete fusion in *T*. *laevis*. These differences may reflect interspecific variation or the degree of fusion may signify different degrees of ageing.

### Description of isolated dermatocranial bones

#### Premaxilla

**Fig 4:**
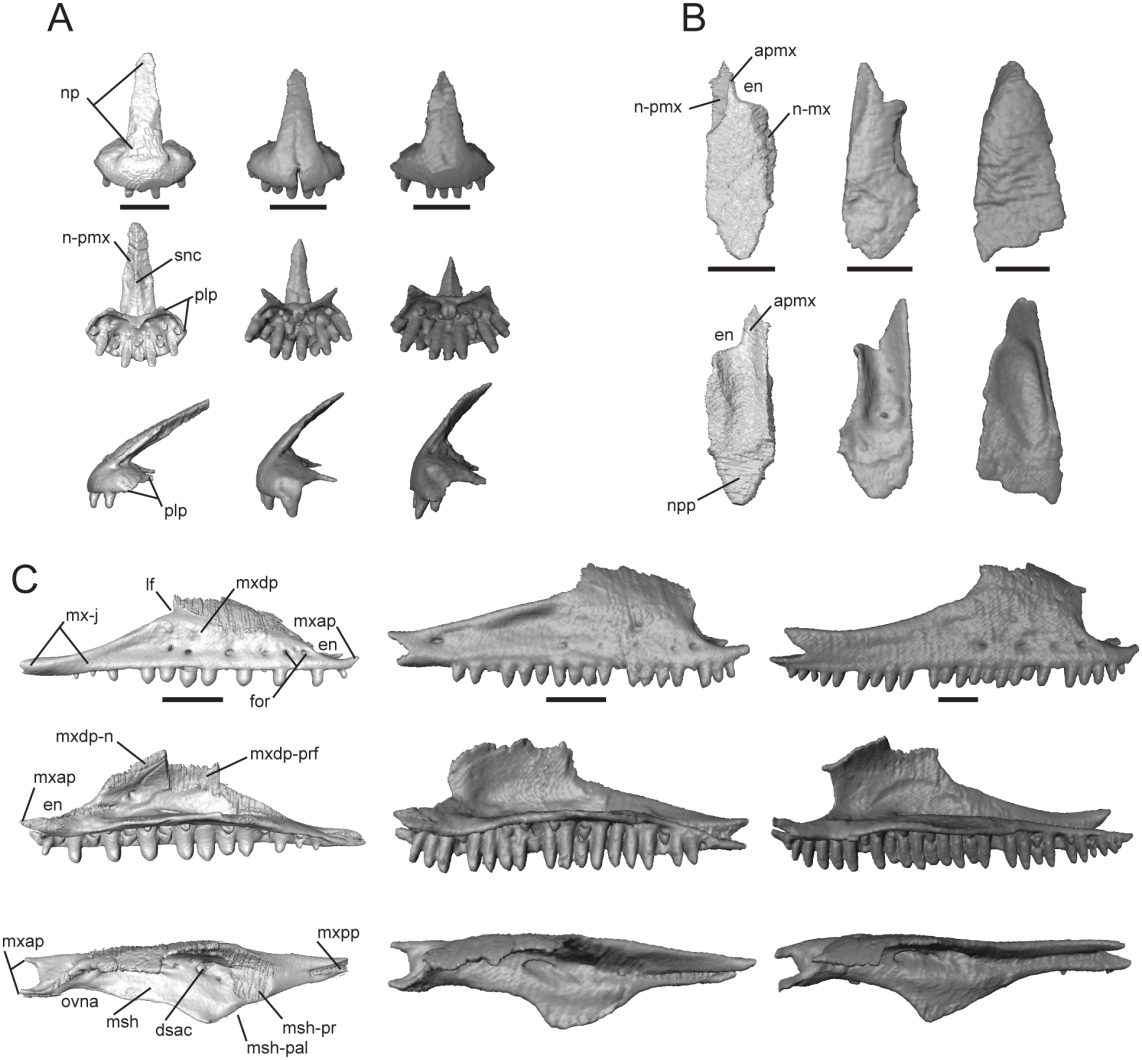
Isolated premaxillae, nasal, and maxilla of *Trachylepis* species examined. Light gray elements = *T*. *laevis*, medium gray elements = *T*. *sulcata*, and dark gray elements = *T*. *gonwouoi*. **A**, premaxillae in anterior (top), ventral (mid), and lateral (bottom) views. **B**, right nasal in dorsal (top) and ventral (bottom) views. **C**, right maxilla in lateral (top), labial (mid), and dorsal (bottom) views. **Abbreviations**: **apmx**, anteromedial premaxillary process; **dsac**, dorsal opening for the superior alveolar canal; **np**, nasal process; **en**, external nares; **for**, maxilla foramen; **lf**, lacrimal foramen; **msh**, medial shelf of the maxilla; **msh-pal**, palatine facet of the medial shelf; **msh-prf**, prefrontal facet of the medial shelf; **mx-j**, jugal facet of the maxilla; **mxap**, premaxillary process of the maxilla; **mxdp**, dorsal process of the maxilla; **mxdp-n**, nasal facet of the dorsal process; **mxdp-prf**, prefrontal facet of the dorsal process; **mxpp**, posterior process of the maxilla; **n-mx**, maxilla facet of the nasal; **n-pmx**, premaxilla facet of the nasal; **npp**, posterior process of the nasal; **ovna**, opening for the vomeronasal apparatus; **plp**, palatal process of the premaxilla; **pmx-n**, nasal process of the premaxilla; **snc**, septonasal crest. Scale bars = 1 mm.

This bone, normally paired in skinks, contacts the maxilla posteroventrally, nasal posterodorsally, and vomer ventromedially. *Trachylepis sulcata* and *T*. *gonwouoi* possess paired premaxillae, while *T*. *laevis* possesses a premaxilla that is fused at the base with a faint suture visible only on the nasal process (S2 Fig). The nasal process (np, Fig 4A) is triangular in dorsal and ventral view. The base of this process is wide, whereas the distal end tapers to a rounded tip in *T*. *laevis* and a pointed tip in *T*. *sulcalta* and *T*. *gonwouoi*. The distal end of the nasal process overlaps the paired nasals (pmx-n, Fig 4A), but does not reach the medial process of the frontal (fmp, Fig 4A). The septonasal crest (snc, Fig 4A) is a raised ridge that runs along the nasal process ventrally; it lies dorsal to the contact point between the paired nasals. The palatal process (plp, Fig 4A) extends posteromedially and is overlapped on its dorsomedial and dorsolateral surfaces by the premaxillary process of the maxilla (mxap, Fig 4C). The palatal process abuts the anterior edge of the vomer and does not participate in the formation of the medial foramen. All three species have nine marginal tooth loci. The teeth are isodont, cylindrical, pleurodont, and have rounded crowns. The tooth positions are evenly spaced in *T*. *laevis*, as each locus adjacent to a functional tooth is empty or in a replacement state. *Trachylepis laevis* has an elongated and sharply angled nasal process, whereas in *T*. *gonwouoi* this structure is short and possesses a low slope (Fig 4A). *Trachylepis sulcata* has a thin, elongated palatal process, and the anteriormost region of the premaxilla is unfused with a suture visible in the proximal nasal process.

#### Nasal

This is a paired bone, which is convex dorsally and roughly three times longer than wide. It contacts the premaxilla anteromedially, maxilla laterally, frontal posteriorly, prefrontal posterolaterally, and has medial contact with the other nasal. The anteromedial premaxillary process (apmx, Fig 4B) bears a shelf facet contacting half of the nasal process of the premaxilla (np, Fig 4A). In dorsal view, the area where the premaxilla lies is clearly visible (n-pmx, Fig 4B). The anterolateral edge is strongly emarginated and borders the posterior margin of the external naris (en, Fig 4B) in *T*. *laevis* and *T*. *sulcata*, but is only slightly emarginated in *T*. *gonwouoi*. The posterior process (npp, Fig 4B) overlaps a depressed region of the frontal that is situated anteromedially between the medial process of the frontal and the lateral process of the frontal. The posterior process is crescent-shaped in *T*. *laevis* and *T*. *sulcata*, but is triangular in *T*. *gonwouoi*. The dorsal process of the maxilla (mxdp, Fig 4C) slightly overlaps the dorsolateral surface of the nasal (n-mx, Fig 4B) in *T*. *laevis* and *T*. *sulcata*, but only abuts the nasal in *T*. *gonwouoi*. The contact between the two nasals is extensive, only being separated by the medial process of the frontal posteriorly and by the nasal process of the premaxilla (np, Fig 4C) anteriorly. A ventral concavity is present in all three taxa, but is most evident in *T*. *gonwouoi*.

#### Maxilla

This paired bone contacts the premaxilla anteriorly, nasal anterodorsally, prefrontal dorsally, jugal and lacrimal posterodorsally, ectopteryogoid posteromedially, and palatine and vomer medially. The anterior region of the medial shelf (msh, Fig 4C) extends towards the vomer and forms the lateral edge of the opening for the vomeronasal apparatus (ovna, Fig 4C). A much greater proportion of the medial shelf borders the vomer in *T*. *laevis* than in the other two taxa (Fig 2G–2I). The medial shelf also forms the floor of the external naris anterodorsally and the anterolateral edge of the choana. The medial shelf contacts the palatine posteromedially (msh-pal, Fig 4C) and the prefrontal posterodorsally (msh-prf, Fig 4C). The anterior premaxillary process (mxap, Fig 4C) is very wide and U-shaped in dorsal view with lateral and medial projections, and the two projections overlap the posteromedial and posterolateral surfaces of the palatal process of the premaxilla (plp, Fig 4A). The medial projection of the anterior premaxillary process overlaps the vomer in *T*. *laevis* and *T*. *gonwouoi*, but is overlapped by the vomer in *T*. *sulcata* (Fig 2G–2I). The dorsal opening for the superior alveolar canal (dsac, Fig 4C) lies at the junction of the medial shelf and the medial surface of the dorsal process. The dorsal process (mxdp, Fig4C) slopes dorsomedially to contact the nasal anteriorly (mxdp-n, Fig 4C) and prefrontal lateromedially (mxnp-prf, Fig 4C); it borders the posterior region of the external naris (en, Fig 4C) and the anterior margin of the lacrimal foramen (lf, Fig 4C). The anterior and posterior margins of the dorsal process have a similar slope in *T*. *laevis*, whereas the anterior edge is more steeply sloped than the posterior edge in *T*. *sulcata* and *T*. *gonwouoi* and the overall height of the dorsal process is greater in the latter two taxa as well. The dorsal process tapers posteriorly and terminates anterior to the posterior process (mxpp, Fig 4C) in *T*. *laevis*, but participates in the posterior process in *T*. *sulcata* and *T*. *gonwouoi*. The posterior process contacts the ectopterygoid medially in all three taxa, is overlapped by the jugal slightly in *T*. *laevis*, and is overlapped greatly by the jugal in *T*. *sulcata* and *T*. *gonwouoi* (mx-j, Fig 2A). The posteriormost region of the posterior process lacks teeth in *T*. *laevis* and *T*. *sulcata*, but retains four teeth in *T*. *gonwouoi*. *Trachylepis laevis* has 16 maxillary tooth loci and 11 teeth, *T*. *sulcata* has 19 tooth loci and 16 teeth, and *T*. *gonwouoi* has 27 tooth loci and 24 teeth. The teeth have the same morphology as those of the premaxilla. The teeth of *T*. *laevis* are more robust and blunt compared to those of its congeners. The nutritive foramina of the teeth and several tooth buds are close to the ventral surface of the medial shelf (msh, Fig 4C).

#### Prefrontal

**Fig 5:**
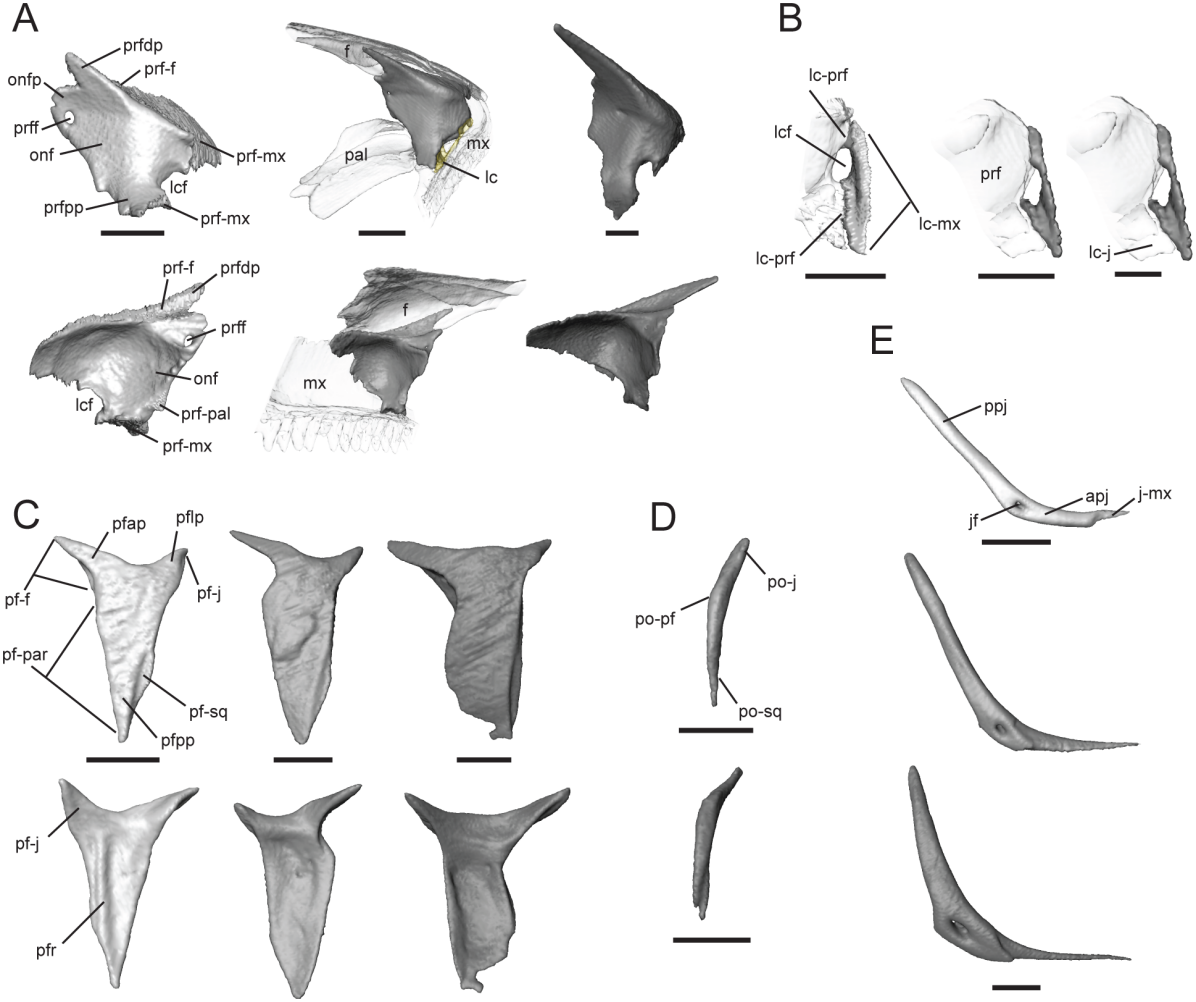
Isolated prefrontal, lacrimal, postfrontal, postorbital, and jugal of *Trachylepis* species examined. Light gray elements = *T*. *laevis*, medium gray elements = *T*. *sulcata*, and dark gray elements = *T*. *gonwouoi*. **A**, right prefrontal in posterolateral (top) and anteromedial (bottom) views. **B**, right lacrimal in posterior view. **C**, right postfrontal in dorsal (top) and ventral (bottom) views. **D**, right postorbital in dorsal view. **E**, right jugal in lateral view. **Abbreviations**: **apj**, anterior process of the jugal; **f**, frontal; **j-mx**, maxilla facet of the jugal; **jf**, jugal foramen; **lc-j**, jugal facet of the lacrimal; **lc-mx**, maxilla facet of the lacrimal; **lc-prf**, prefrontal facet of the lacrimal; **lcf**, lacrimal foramen; **mx**, maxilla; **onf**, orbitonasal flange; **onfp**, orbitonasal flange projection; **pal**, palatine; **pf-f**, frontal facet of the postfrontal; **pf-j**, jugal facet of the postfrontal; **pf-par**, parietal facet of the postfrontal; **pf-sq**, squamosal facet of the postfrontal; **pfap**, anterior process of the postfrontal; **pflp**, lateral process of the postfrontal; **pfpp**, posterior process of the postfrontal; **pfr**, ventral ridge of the postfrontal; **po-j**, jugal facet of the postorbital; **po-pf**, postfrontal facet of the postorbital; **po-sq**, squamosal facet of the postorbital; **ppj**, posterior process of the jugal; **prf**, prefrontal; **prf-f**, frontal facet of the prefrontal; **prf-pal**, palatine facet of the prefrontal; **prfdp**, dorsal process of the prfrontral; **prff**, prefrontal foramen; **prfpp**, posterior process of the prefrontal. Scale bars = 1 mm.

The prefrontal is a paired bone that contacts the maxilla anterolaterally and ventrally, frontal dorsally, palatine medially, nasal anterodorsally, and lacrimal laterally. The prefrontal narrowly contacts the jugal only in *T*. *gonwouoi*. The dorsolateral surface of the prefrontal is overlapped by the posterior portion of the dorsal process of the maxilla and the lateral process of the frontal (Fig 5A). The ventrolateral region is convex and forms the medial margin of the lacrimal foramen (lcf, Fig 5A). Posterior to the lacrimal foramen, the prefrontal posterior process (prfpp, Fig 5A) abuts the maxilla ventrally and lacrimal laterally. The prefrontal curves posteromedially forming the orbitonasal flange (onf, Fig 5A), which contacts the palatine medially (prf-pal, Fig 5A) and the frontal dorsally (prf-f, Fig 5A), separating these two bones with a small projection (onfp, Fig 5A). A prefrontal foramen (prff, Fig 5A) pierces the orbitonasal flange. This foramen is relatively large in *T*. *laevis* and very small in *T*. *sulcata* and *T*. *gonwouoi*. The dorsal process of the prefrontal (prfdp, Fig 5A) is directed posterodorsally and tapers gradually, contacting the frontal medially (f-prf, Fig 5A). The dorsal and posterior processes are more elongated and sharply tapered in *Trachylepis gonwouoi*.

#### Lacrimal

This paired bone contacts the prefrontal medially (lc-prf, Fig 5B) and the posterior process of the maxilla (lc-mx, Fig 5B) ventrally and laterally. The medial edge of the lacrimal forms the posterolateral margin of the lacrimal foramen (lcf, Fig 5B). The posterior region of the lacrimal has medial contact with the anterior process of the jugal in *T*. *gonwouoi* (lc-j, Fig 5B), nearly contacts the jugal in *T*. *sulcata*, and is widely separated from the jugal in *T*. *laevis*.

#### Postfrontal

This is a paired bone that is Y-shaped in outline. It clasps the frontoparietal suture, contacting the frontal anteromedially (pf-f, Fig 5C) and the parietal posteromedially (pf-par, Fig 5C), forming a lateral brace for the movable frontoparietal suture [55]. The postfrontal is strongly concave medially in *T*. *gonwouoi* due to the convex projection produced by the anterolateral portion of the parietal and posterolateral region of the frontal. The anterior process (pfap, Fig 5C) is wide at the base and tapers to a pointed tip. The lateral process (pflp, Fig 5C) is shorter than the anterior process, curves ventrolaterally, and nearly contacts the jugal laterally in *T*. *laevis*, narrowly contacts the jugal in *T*. *sulcata*, and has extensive contact of the jugal in *T*. *gonwouoi* (pf-j, Fig 5C). The lateral process of *T*. *sulcata* and *T*. *gonwouoi* extends further ventrally than in *T*. *laevis* (Fig 2D–2F). The entirety of the postfrontal in lateral view is relatively flat in *T*. *laevis*, weakly concave in *T*. *sulcata*, and strongly so in *T*. *gonwouoi* (Fig 2D–2F), corresponding to the domedness of the cranial vault in these taxa. The posterior process (pfpp, Fig 5C) is much broader than the anterior and lateral processes and a lateral facet is present for articulation with the anterior process of the squamosal (pf-sq, Fig 5C). A ventral ridge (pfr, Fig 5C) is present on the postfrontal in *T*. *laevis*. The postfrontal contacts the postorbital laterally in *T*. *sulcata* and *T*. *gonwouoi*. *Trachylepis gonwouoi* has a posteromedial notch on the posterior process of the postfrontal, and the postfrontal of *T*. *sulcata* possesses dorsal sculpturing.

#### Postorbital

This paired bone is either completely fused with the postfrontal or lost in *T*. *laevis*, but is present in *T*. *sulcata* and *T*. *gonwouoi*. This bone is slender and curved anteroventrally. The postorbital contacts the postfrontal (po-pf, Fig 5D) medially, the squamosal (po-sq, Fig 5D) posterolaterally, and the jugal (po-j, Fig 5D) anterolaterally. The surface contact between the squamosal and postorbital is greater in *T*. *sulcata* than in *T*. *gonwouoi*; however, a posterolateral facet is present in *T*. *gonwouoi* that is overlapped by the anterior process of the squamosal.

#### Jugal

This is a paired, elongated, strut-like bone that resembles a hockey stick. The anterior process is much narrower, flaring out dorsally into the posterior process, which keeps a constant width in *T*. *laevis* and *T*. *sulcata*, but tapers distally in *T*. *gonwouoi*. The posterior process is rounded, while the anterior process is pointed. Anteriorly, the jugal overlies the maxilla and extends beyond its posterior margin. It forms an articulation with the medially adjacent ectopterygoid, which prevents contact with the pterygoid flange. The anterior process of the jugal (apj, Fig 5E) is very short in *T*. *laevis*, barely extending beyond the length of the ectopterygoid and only slightly overlapping the posterior process of the maxilla (j-mx, Fig 5E), whereas the anterior process in *T*. *sulcata* and *T*. *gonwouoi* extends well beyond the ectopterygoid, overlaps the posterior process of the maxilla nearly entirely, and narrowly contacts the prefrontal medially and lacrimal laterally. The jugal does not participate in the lacrimal foramen in these two species, but medially it borders a shallow groove that is a continuation of this foramen. A jugal foramen (jf, Fig 5E) is present at the point of inflection, and this region is swollen in *T*. *gonwouoi*. A facet is present anterior to the foramen where the posterior process of the maxilla overlaps laterally, forming a butt-lap joint. This facet is more posterior in *T*. *sulcata* and *T*. *gonwouoi* than in *T*. *laevis*. The jugal is strongly recumbent in *T*. *laevis* (144°) but less so in *T*. *sulcata* (130°) and *T*. *gonwouoi* (125°).

#### Frontal

**Fig 6:**
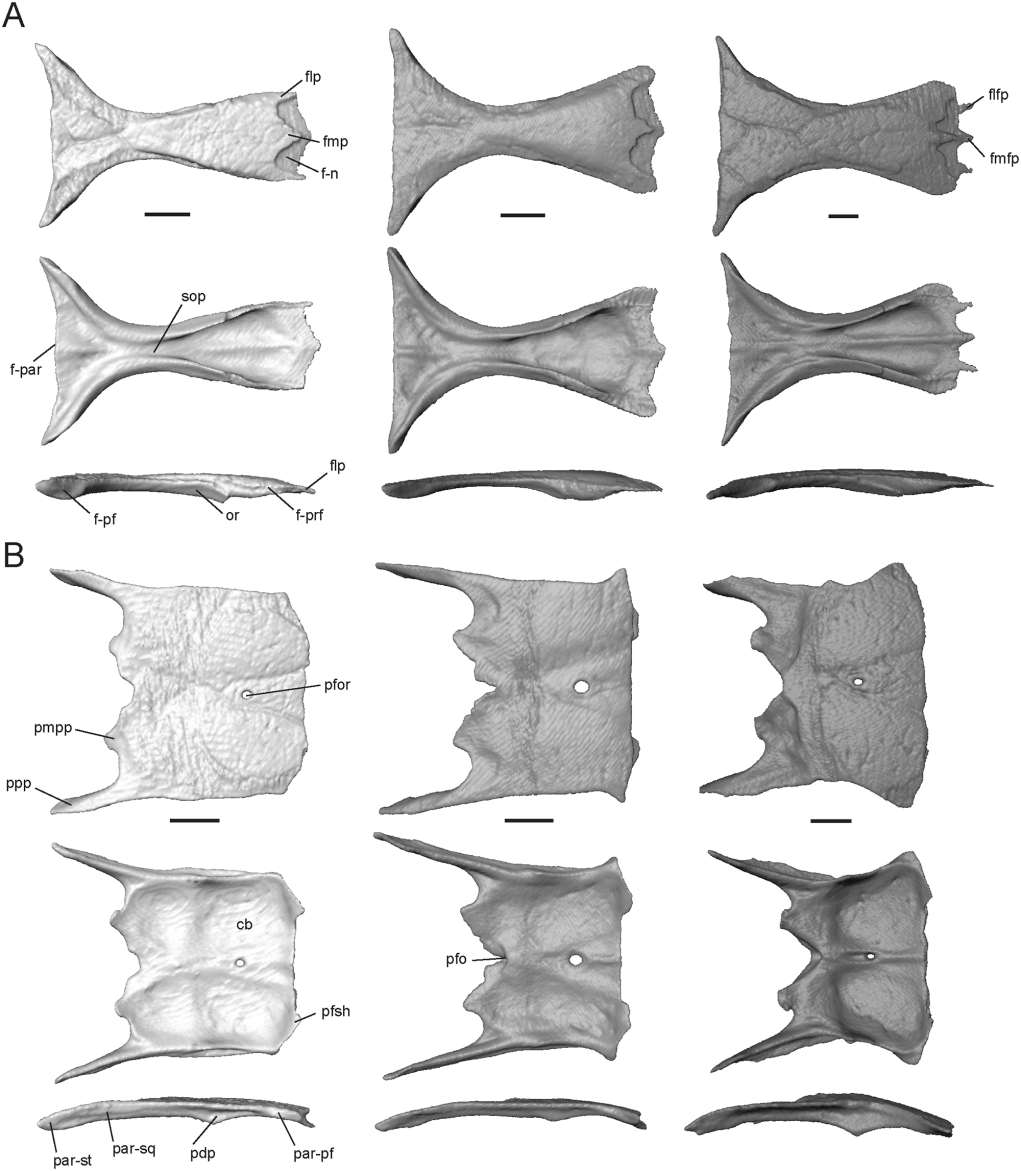
Isolated frontal and parietal of *Trachylepis* species examined. Light gray elements = *T*. *laevis*, medium gray elements = *T*. *sulcata*, and dark gray elements = *T*. *gonwouoi*. **A**, frontal in dorsal (top), ventral (mid), and lateral (bottom) views. **B**, parietal in dorsal (top), ventral (mid), and lateral (bottom) views. **Abbreviations**: **cb**, concavity of the parietal; **f-n**, nasal facet of the frontal; **f-par**, parietal facet of the frontal; **f-prf**, prefrontal facet of the frontal; **flfp**, lateral facet process of the frontal; **flp**, lateral process of the frontal; **fmfp**, medial facet process of the frontal; **fmp**, medial process of the frontal; **or**, orbital ridge; **par-pf**, postfrontal facet of the parietal; **par-sq**, squamosal facet of the parietal; **par-st**, supratemporal facet of the parietal; **pdp**, descending process of the parietal; **pfor**, parietal foramen; **pfo**, parietal fossa; **pfsh**, parietal shelf for the frontal; **pmpp**, posteromedial process of the parietal; **ppp**, posterior process of the parietal; **sop**, subolfactory process. Scale bars = 1 mm.

This is an unpaired, hourglass-shaped bone that is overlapped by the nasals anteriorly. It has contact with the dorsal process of the prefrontal anterolaterally, postfrontal posterolaterally, and parietal posteriorly. The medial process (fmp, Fig 6A) is variable in shape, ranging from a 70° triangle in *T*. *laevis* to a 35° triangle in *T*. *gonwouoi*. The lateral processes (flp, Fig 6A) of *T*. *laevis* are pointed, thinner, and longer than the medial process, while the lateral processes of *T*. *sulcata* and *T*. *gonwouoi* are rounded and much wider than the medial process. Depressed facets are present between the lateral and medial processes, which are overlapped by the posterior processes of the nasals (npp, Fig 4B) and leave the medial and lateral processes exposed. *Trachylepis gonwouoi* has additional medial (fmfp, Fig 6A) and lateral facet processes (flfp, Fig 6A), which are short, pointed projections that are also overlapped by the posterior process of the nasal. The anterior end of the frontal is roughly half the width of the posterior end. The latter is slightly curved in *T*. *laevis*, but is straight in its congeners. Each posterolateral margin overlaps the parietals in a hinge-like articulation for the uplift of the muzzle unit. The crista cranii, formed by the descending orbital ridges (or, Fig 6A) and subolfactory processes (sop, Fig 6A), are not fused ventrally; therefore, no tubular structure is present to encase the olfactory tracts. However, concavities are visible on the venter of the frontal to accommodate these structures. A lateral division of the ventral concavities is present in *T*. *laevis* and *T*. *gonwouoi*, but not in *T*. *sulcata*. The minimal interorbital width is 22% (in *T*. *laevis*), 21% (in *T*. *sulcata*), and 30% (in *T*. *gonwouoi*) of the respective maximum frontoparietal suture width. Dorsal surface sculpturing is present in all three taxa due to the fusion of the head shield osteoderms to the underlying bone (corresponding to the frontal, frontoparietal, and interparietal scales), although it is most prevalent in *T*. *gonwouoi*. The maximum thickness of the frontal table at the orbital ridge is variable, being thinnest in *T*. *laevis* (0.26 mm) and thickest in *T*. *sulcata* (0.41 mm).

#### Parietal

This is an unpaired element that contacts the frontal anteriorly, postfrontal anterolaterally, prootic posteroventrally, squamosal and supratemporal posterolaterally, and supraoccipital posteriorly. With the frontal, the parietal forms a relatively flat skull table in *T*. *laevis* and *T*. *sulcata*, but the parietal is convex in *T*. *gonwouoi* yielding a slanted frontal and domed skull table (Fig 2D–2F). The parietal is subrectangular; the anterior edge is slightly concave and bears two small shelves or tabs that are overlapped by the frontal (pfsh, Fig 6B). The parietal of *T*. *sulcata* and *T*. *gonwouoi* have elongated anterolateral projections that correspond to elongated posterolateral projections of the frontal, forming a concave medial edge of the postfrontal (Fig 2A–2C). A parietal foramen (pfor, Fig 6B) is present in all three taxa, however, it is reduced in *T*. *gonwouoi*. The posterior edge of the parietal bears two elongate posterior processes (ppp, Fig 6B) and two short posteromedial processes (pmpp, Fig 6B), forming a posterior notch. The posterior processes have an extended lateral contact with the squamosal and extend posteriorly over the paroccipital process (Fig 2A). Only in *T*. *gonwouoi* do the posteromedial processes overlap the prootic and project lateral to the processus ascendens of the supraoccipital. The relative length of the posterior processes varies, being 28% of the total parietal length in *T*. *sulcata*, 42% of the total parietal length in *T*. *sulcata*, and 38% of the total parietal length in *T*. *gonwouoi*. A short, ventrally-oriented descending process (pdp, Fig 6B) is present on the lateral surface of the parietal in all three taxa. This process does not directly contact the dorsal head of the epipterygoid in the specimens examined (Fig 2B), however it is extended most ventrally in *T*. *gonwouoi*, nearly contacting the epipterygoid. Anterolaterally, there is a facet for the posterior process of the postfrontal (par-pf, Fig 6B). Ventrally, there is a concavity that may accommodate the hemispheres of the cerebrum, optic lobe, and cerebellum (cb, Fig 6B). This concavity is most prominent in *T*. *gonwouoi*. A midventral ridge is present in *T*. *sulcata* and *T*. *gonwouoi*, however in *T*. *sulcata* it extends only from the parietal foramen to the frontal. A midventral parietal fossa (pfo, Fig 6B) is present on the posterior edge of the parietal in *T*. *sulcata* and *T*. *gonwouoi* between the two posteromedial processes. Dorsal surface sculpturing is present in all three taxa due to the fusion of the head shield osteoderms to the underlying bone (corresponding to the interparietal and parietal scales).

#### Squamosal

**Fig 7:**
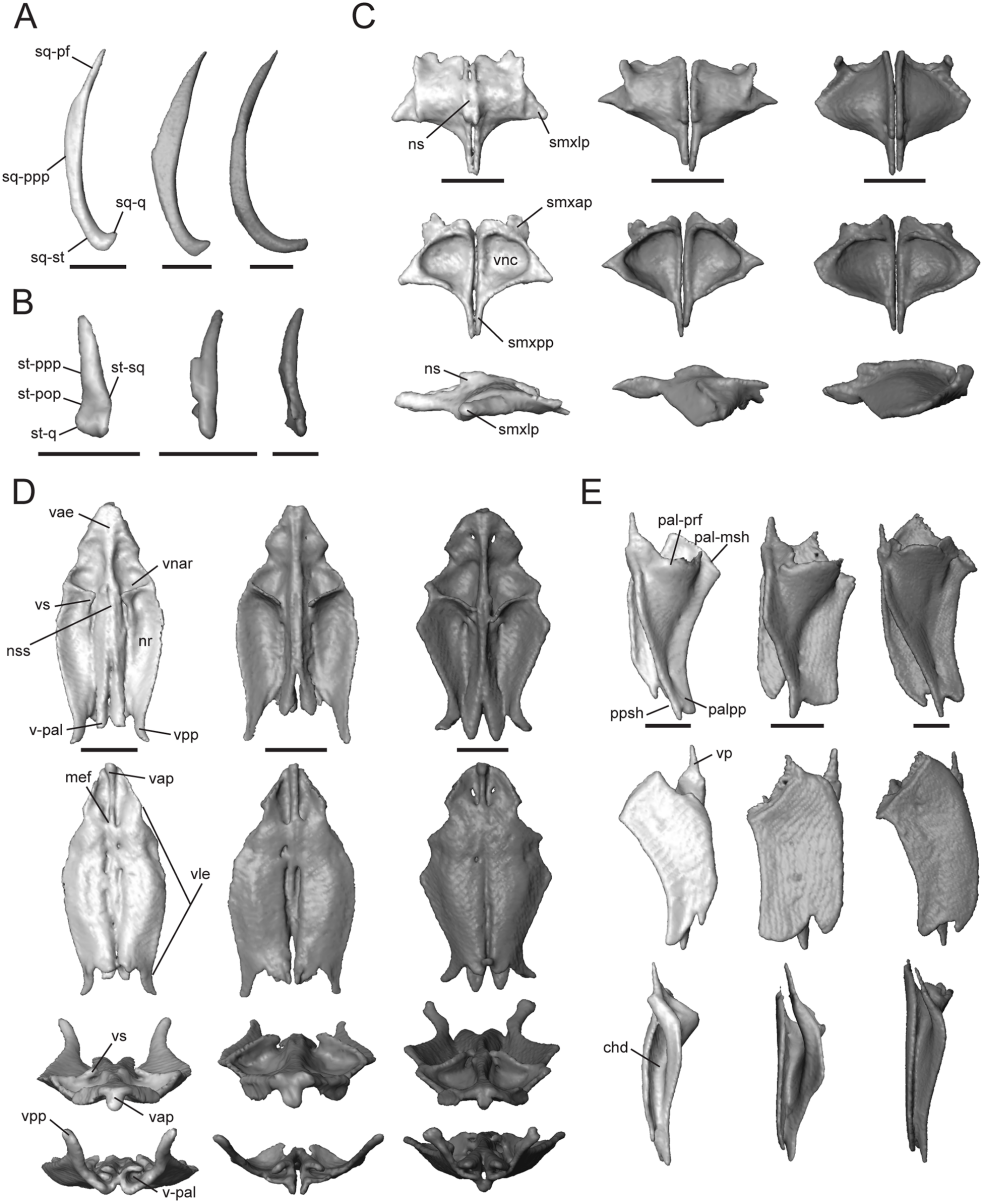
Isolated squamosal, supratemporal, septomaxilla, vomer, and palatine of *Trachylepis* species examined. Light gray elements = *T*. *laevis*, medium gray elements = *T*. *sulcata*, and dark gray elements = *T*. *gonwouoi*. **A**, right squamosal in dorsal view. **B**, right supratemporal in dorsal view. **C**, septomaxillae in dorsal (top), ventral (mid), and lateral (bottom) views. **D**, vomer in dorsal (top), ventral (second row), anterior (third row) and posterior (bottom) views. **E**, right palatine in dorsal (top), ventral (mid), and medial (bottom) views. **Abbreviations**: **chd**, choanal duct; **mef**, medial foramen of the vomer; **nr**, nasal region of the vomer; **ns**, nasal septum; **nss**, space for the nasal septum; **pal-msh**, medial shelf facet of the palatine; **pal-prf**, prefrontal facet of the palatine; **palpp**, pterygoid process of the palatine; **ppsh**, pterygoid process shelf; **smxap**, anterior process of the septomaxilla; **smxlp**, lateral process of the septomaxilla; **smxpp**, posterior process of the septomaxilla; **sq-pf**, postfrontal facet of the squamosal; **sq-ppp**, posterior process of the parietal facet for the squamosal; **sq-q**, quadrate facet of the squamosal; **sq-st**, supratemporal facet of the squamosal; **st-pop**, paroccipital process facet of the supratemporal; **st-ppp**, posterior process of the parietal facet of the supratemporal; **st-q**, quadrate facet of the supratemporal; **st-sq**, squamosal facet of the supratemporal; **v-pal**, palatine facet of the vomer; **vae**, anterior edge of the vomer; **vap**, anterior vomerine process; **vle**, lateral edge of the vomer; **vnar**, vomeronasal region of the vomer; **vnc**, concavity of the vomeronasal apparatus; **vpp**, posterior process of the vomer; **vs**, vomerine septum. Scale bars = 1 mm.

This is a paired element that contacts the postparietal process of the parietal dorsomedially (sq-ppp, Fig 7A), supratemporal posteromedially (sq-st, Fig 7A), postfrontal anteromedially (sq-pf, Fig 7A), quadrate posteroventrally (sq-q, Fig 7A), postorbital anteromedially (except for *T*. *laevis*, which lacks this element), and overlays the prootic and the otoccipital. The squamosal is a thin, elongate bone that curves posterolaterally, forming a hockey stick shape [4]. This bone plays a role in quadrate suspension in these taxa and interacts with the squamosal notch of the quadrate (Fig 2A–2C). The squamosal of *T*. *sulcata* and *T*. *gonwouoi* is relatively longer than that of *T*. *laevis*. The inflection point of the long axis is more posterior in *T*. *laevis* and *T*. *gonwouoi* as compared to *T*. *sulcata*. The curvature of the squamosal is most extreme in *T*. *gonwouoi*.

#### Supratemporal

This paired element contacts the paroccipital process (st-pop, Fig 7B) posteromedially, quadrate (st-q, Fig 7B) posterolaterally, posterior process of the parietal (st-ppp, Fig 7B) anteromedially, and squamosal (st-sq, Fig 7B) anterolaterally. The supratemporal of *T*. *laevis* is proportionally the shortest and has the most limited contact with the posterior process of the parietal and paroccipital process (Fig 2M–2O). In *T*. *sulcata*, a medial facet contacts the paroccipital process (Fig 7B), and in *T*. *gonwouoi* the supratemporal is curved anterolaterally (Fig 7B).

#### Septomaxilla

This is a paired bone (Fig 7C), which covers the vomeronasal apparatus (or Jacobson’s organ) dorsally. In ventral view, the concavity occupied by the vomeronasal apparatus (vnc, Fig 7C; or concha vomeronasalis) is visible. The dorsal surface of the septomaxilla forms the anterior part of the nasal capsule, and in *T*. *laevis*, the nasal septum is visible tightly joining the two bones (ns, Fig 7C, S3 Fig). The lateral process (smxlp, Fig 7C) is triangular and angled posterolaterally in *T*. *laevis*, triangular and angled laterally in *T*. *sulcata*, and is rounded and angled laterally in *T*. *gonwouoi*. This process abuts the maxillary surface medially. The posterior process (smxpp, Fig 7C) is a thin and prong-like projection that is proportionally longer in both *T*. *laevis* and *T*. *sulcata* than in *T*. *gonwouoi*. The anterior process (smxap, Fig 7C) extends into the cavity of the external naris anteriorly in *T*. *laevis* and *T*. *gonwouoi* and dorsoanteriorly in *T*. *gonwouoi* (Fig 2D).

#### Vomer

The vomer is a fused laminar bone. It contacts the premaxilla and maxilla anteriorly, septomaxilla dorsally, and palatine posteriorly. The central roof of the mouth and the anterior primary palate is formed by the vomer, the palatal process of the premaxilla (plp, Fig 4A), and the medial shelf of the maxilla (msh, Fig 4C). The anterior edge of the vomer is rounded, encasing paired medial foramina at the anterior end of two grooves on the ventral surface (mef, Fig 7D; or incisive foramen). The anterior vomerine process (vap, Fig 7D) is seen in ventral view and divides the medial foramina posteriorly. This process is fused to the anterior edge of the vomer (vae, Fig 7D) and extends slightly beyond it, contacting the premaxilla. The anterior edge of the vomer (vae, Fig 7D) contacts the premaxilla–maxilla suture (Fig 2C). The lateral edge (vle, Fig 7D) forms the medial boundary of the opening for the vomeronasal apparatus (ovna, Fig 2C), and the posterolateral edge forms the anteriormedial boundary of the choana. The posterior region of the vomer is M-shaped in dorsal and ventral view, having two medial processes that contact the vomerine process of the palatine (v-pal, Fig 7D) and two lateral processes (vpp, Fig 7D) that extend dorsolaterally above the height of the palatine and nearly contact the projection of the orbitonasal flange of the prefrontal. The medial processes are rounded in outline and much shorter than the lateral process in *T*. *laevis* and *T*. *sulcata*, whereas the medial processes are jagged-edged and extend beyond the length of the lateral processes in *T*. *gonwouoi*. The dorsal surface has vomeronasal and nasal regions (vnar and nr, Fig 7D) that are separated by a septum (vs, Fig 7D). The space for the nasal septum and the articulation of the septomaxilla is visible (nss, Fig 7D). *Trachylepis laevis* has a proportionately more elongate vomer than *T*. *sulcata* and *T*. *gonwouoi*. The vomeronasal region and septum is reduced in size in *T*. *laevis* compared to *T*. *sulcata* and *T*. *gonwouoi*.

#### Palatine

This is a paired element that contacts the vomer anteriorly, prefrontal anterodorsally, pterygoid posteriorly, and maxilla laterally. The palatine forms the anteromedial border of the suborbital fenestra and the posterior border of the choana (sof and c, Fig 2A and 2C). The palatine is highly three-dimensional, having a flat venter and a lateral flange that is dorsomedially raised. The medial edge of the flange is suspended and borders the anterior portion of the interpterygoid vacuity. In medial view, the continuation of the hollow choanal duct (chd, Fig 7E) is visible between the venter and flange of the palatine. Anteriorly, it has a slender vomerine process (vp, Fig 7E) that conctacts the vomer (v-pal, Fig 7D). The anterior edge curves dorsolaterally, contacting the medial shelf of the maxilla (pal-msh, Fig 7E) and the orbitonasal flange of the prefrontal (pal-prf, Fig 7E). The pterygoid process (palpp, Fig 7E) is variable in length and width in these taxa (narrowest and longest in *T*. *laevis*, broadest and shortest in *T*. *sulcata*, intermediate in *T*. *gonwouoi*), but is much larger than the vomerine process in all cases. Dorsally, the pterygoid process of the palatine bears a shelf that receives the palatine process of the pterygoid (ppsh, Fig 7E), forming a tongue-in-groove articulation [56]. The lateral edge of the palatine is concave, and the posterolateral edge borders a thin opening that extends medially from the suborbital fenestra. The lateral concavity is most extreme in *T*. *laevis*. The anterolateral edge contacts the suture between the medial shelf of the maxilla and the prefrontal (pal-mprf; Fig 7E). The orbitonasal flange of the prefrontal prevents contact between the two short pegs of the crista cranii of the frontal and the palatine in these taxa.

#### Pterygoid

**Fig 8:**
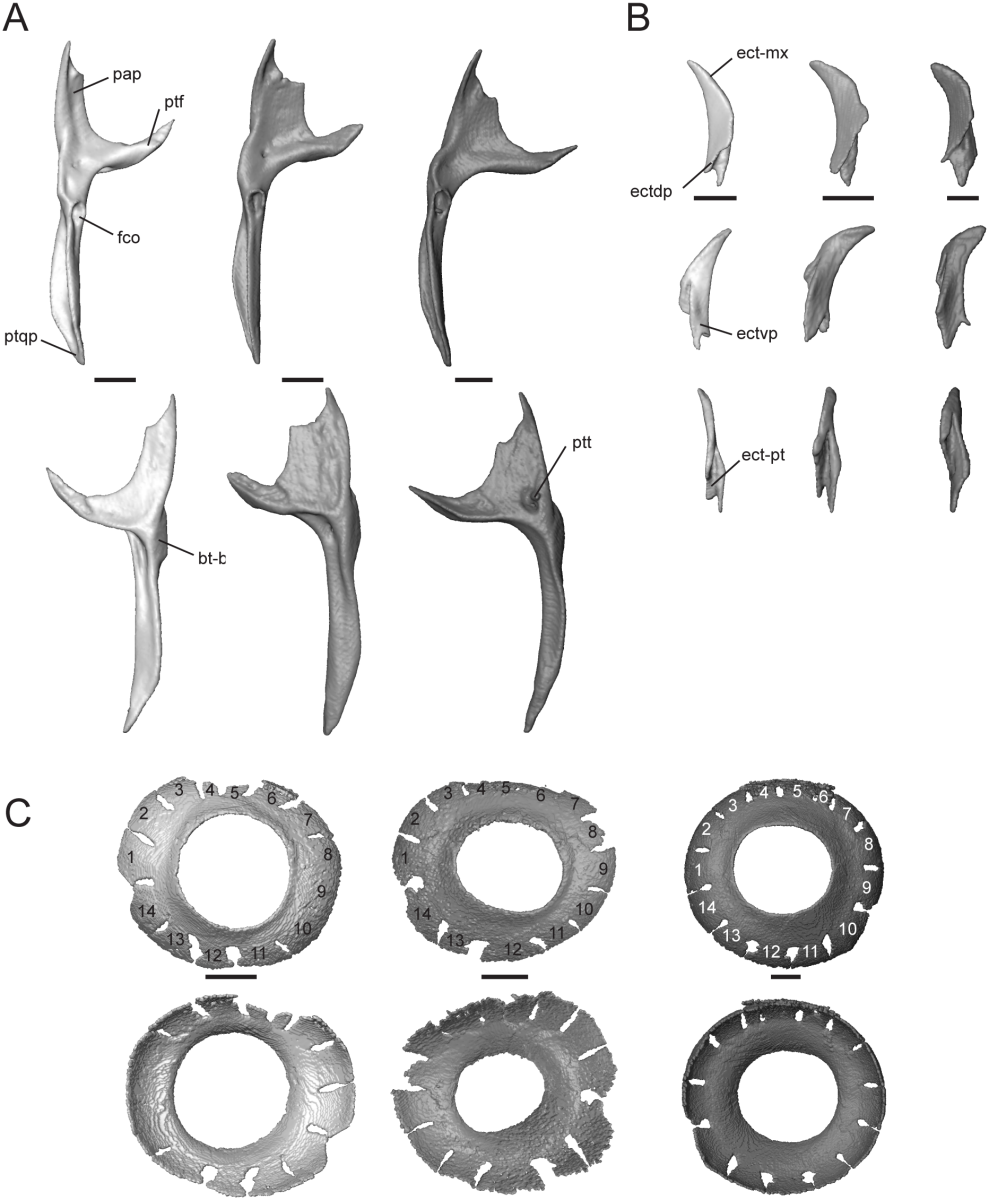
Isolated pterygoid, ectopterygoid, and scleral ring of *Trachylepis* species examined. Light gray elements = *T*. *laevis*, medium gray elements = *T*. *sulcata*, and dark gray elements = *T*. *gonwouoi*. **A**, right pterygoid in dorsal (top) and ventral (bottom) views. **B**, right ectopterygoid in dorsal (top), ventral (mid), and medial (bottom) views. **C**, right scleral ring in lateral (top) and medial (bottom) views. Scleral rings labeled 1–14. **Abbreviations**: **ect-mx**, maxilla facet of the ectopterygoid; **ect-pt**, pterygoid facet of the ectopterygoid; **ectdp**, dorsal process of the ectopterygoid; **ectvp**, ventral process of the ectopterygoid; **fco**, fossa columellae; **pap**, palatine process of the pterygoid; **pt-bs**, basipterygoid facet of the pterygoid; **ptf**, pterygoid flange; **ptqp**, quadrate process of the pterygoid; **ptt**, pterygoid teeth. Scale bars = 1 mm.

This paired bone contacts the palatine and ectopterygoid anteriorly, epipterygoid dorsally, sphenoid medially, and quadrate posterolaterally. The anterolateral edge is strongly concave, making the bone roughly Y-shaped. The palatine process (pap, Fig 8A) articulates with the palatine and is much broader and shorter in *T*. *sulcata* and *T*. *gonwouoi* than in *T*. *laevis*.

It is 45% of the total pterygoid length in *T*. *laevis*, 33% in *T*. *sulcata*, and 36% in *T*. *gonwouoi*. The pterygoid flange (ptf, Fig 8A) is arcuate, curves anterolaterally, and firmly articulates with the ectopterygoid. The distal, anterior edge of the palatine process is concave and forms the posterior border of the thin opening that extends medially from the suborbital fenestra. The lateral edge of the palatine process is also concave, forming the posteromedial edge of the oval suborbital fenestra (Fig 2C) Posterior to the concavity, the bone projects anterolaterally to form the pterygoid flange. The quadrate process (ptqp, Fig 8A) originates behind the fossa columellae (fco, Fig 8A), which accommodates the base of the epipterygoid bone. The process extends posteriorly in a straight line in *T*. *laevis* and *T*. *sulcata*, and curves laterally in *T*. *gonwouoi*, to contact the posteromedial edge of the quadrate. The knob-like basipterygoid process of the pterygoid (pt-bs, Fig 8A) contacts the basipterygoid and increases the tendency of the pterygoids to spread apart [57]. The basipterygoid of the sphenoid and the basipterygoid process of the pterygoid are well separated. Two pterygoid teeth (ptt, Fig 8A) are present within a depression on the venter of the pterygoid of *T*. *gonwouoi*, but no pterygoid teeth are present on *T*. *laevis* or *T*. *sulcata*.

#### Ectopterygoid

This is a paired, crescent-shaped bone that contacts the maxilla (ect-mx, Fig 8B) anteroventrally, pterygoid (ect-pt, Fig 8B) posteriorly, and the jugal anterolaterally. The crescent extends anterolaterally and forms the posterolateral border of the suborbital fenestra. No depression or facet is present on the ectoperygoid for contact with the maxilla or jugal. The posterior region has a deep triangular concavity that clasps the pterygoid flange dorsally and ventrally (ect-pt, Fig 8B), thus forming a dorsal (ectdp, Fig 8B) and ventral (ectvp, Fig 8B) process for the support of the pterygoid flange, as seen in geckos [49]. The ectopterygoid separates the pterygoid and maxilla. The dorsal process of the ectopterygoid (ectdp, Fig 8B) is shorter in *T*. *gonwouoi* than in *T*. *laevis* and *T*. *sulcata* (Fig 8B).

#### Scleral ring

The periphery of the eye is covered by 14 scleral ossicles in all three taxa (Fig 8C), although three anterior ossicles are completely fused together in *T*. *laevis*. The ossicles are roughly the same size and shape and appear to be narrower dorsally in all species. The aperture diameter is approximately 55% the external scleral ring diameter in *T*. *laevis*, 46% in *T*. *sulcata*, and 52% in *T*. *gonwouoi*. The ossicles are more strongly overlapped in *T*. *gonwouoi* than the two other taxa.

### Description of isolated splanchonocranial bones

The articular bone is discussed with the other bones of the jaw (see below).

#### Epipterygoid

**Fig 9:**
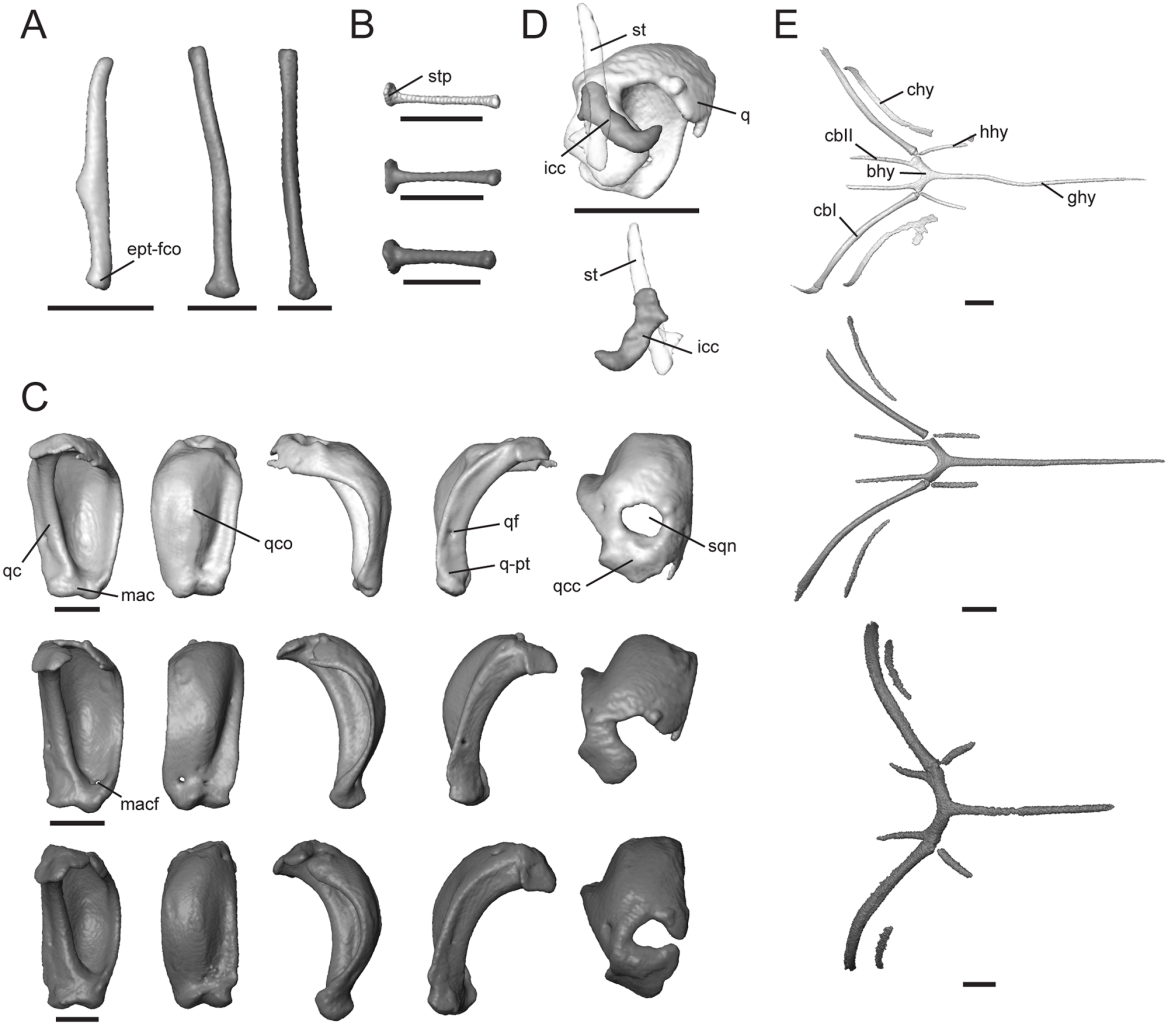
Isolated epipterygoid, stapes, quadrate, intercalary cartilage, and hyoid apparatus of *Trachylepis* species examined. Light gray elements = *T*. *laevis*, medium gray elements = *T*. *sulcata*, and dark gray elements = *T*. *gonwouoi*. **A**, right epipterygoid in anterior view. **B**, right stapes in posterior view. **C**, right quadrate in the following views from left to right: posterior, anterior, lateral, medial, dorsal. **D**, right intercalary cartilage in dorsal (top) and ventral (bottom) views. **E**, hyoid apparatus in ventral view. **Abbreviations**: **bhy**, basihyal; **cbI**, first ceratobranchial; **cbII**, second ceratobranchial; **chy**, ceratohyal; **ept-fco**, pterygoid facet of the epipterygoid; **ghy**, glossohyal; **hhy**, hypohyal; **icc**, intercalary cartilage; **mac**, mandibular condyle of the quadrate; **macf**, mandibular condyle foramen; **q**, quadrate; **q-pt**, pterygoid facet of the quadrate; **qc**, quadrate medial column; **qcc**, quadrate cephalic condyle; **qco**, quadrate conch; **qf**, quadrate foramen; **sqn**, squamosal notch; **st**, supratemporal; **stp**, oval footplate of stapes. Scale bars = 1 mm.

This is a columnar bone that is tilted posteriorly at an angle of 28° from the vertical in *T*. *laevis*, 20° from the vertical in *T*. *sulcata*, and 10° from the verticle in *T*. *gonwouoi* (Fig 2C). The epipterygoid extends from the pterygoid fossa columellae to the region of the descending process of the parietal and the crista alaris of the prootic; however, it does not make direct contact, but rather a ligamentous contact, with the parietal and neurocranium (Fig 2C). This element has a discrete expansion ventrally at the pterygoid contact (ept-fco, Fig 9A). The epipterygoid of *T*. *laevis* and *T*. *sulcata* are slightly curved, while this element is straight in *T*. *gonwouoi*. This bone is notably shorter and slightly thicker at its center in *T*. *laevis* compared to the other two taxa (Fig 9A).

#### Stapes

In combination with the cartilaginous extrastapes, this bone forms the columella auris. The oval footplate (stp, Fig 9B) fits into the fenestra ovalis of the braincase. The stapes approaches the medial column of the quadrate (Fig 2C), but does not make bone-to-bone contact. The stapes of *T*. *laevis* is thinner and more elongate than that of its congeners. The stapedial foramen is entirely enclosed distal to the oval footplate.

#### Quadrate

The quadrate is a prominent bone with a large posterior concavity. It contacts the squamosal and supratemporal dorsally, otooccipital dorsomedially, pterygoid posteromedially, and articular ventrally. It has a smooth, conch-like, convex anterior surface (qco, Fig 9C). Part of the space that is produced by the concavity of the quadrate supports the tympanic membrane and forms the auditory meatus. The cephalic condyle is positioned dorsomedially (qcc, Fig 9C), contacting the paroccipital process of the otooccipital, squamosal, and supratemporal, and is therefore at least partially suspended by “paroccipital abutting” [55]. A squamosal notch (sqn, Fig 9C) lies anterolaterally to the cephalic condyle. This notch is completely enclosed in *T*. *laevis*, laterally exposed in *T*. *sulcata*, and nearly enclosed in *T*. *gonwouoi*. There is a medial column (qc, Fig 9C) that extends between the cephalic condyle and mandibular condyle (mac, Fig 9C). This column is constricted near the base of the cephalic condyle and expands laterally as it approaches the mandibular condyle. The medial edge of the column is smooth and a quadrate foramen is present (qf, Fig 9C). The mandibular condyle is concave and appears as a distinct medial and lateral condyle. A pterygoid facet is present slightly above the medial mandibular condyle where it contacts the pterygoid (q-pt, Fig 9C). In lateral view, the quadrate is crescent-shaped and the point of inflection lies at the midbody. The inflection is most extreme in *T*. *laevis* (Fig 2C). The tympanic crest is not enlarged, but projects anteriorly where it outlines the anterior edge of the auditory meatus and the union of the tympanic membrane. On the anterior surface of the quadrate of *T*. *sulcata*, there is an oval foramen (macf, Fig 9C) above the mandibular condyle. The quadrate is recumbent in relation to the horizontal quadrate process of the pterygoid, forming a 45° angle from the vertical in *T*. *laevis*, 40° angle from the vertical in *T*. *sulcata*, and 35° angle from the vertical in *T*. *gonwouoi* (measured from the quadrate-pterygoid articulation to the quadrate-paroccipital articulation). In anterior view, the quadrate of *T*. *laevis* in widest in relation to height relative to the other species (Fig 9C). A small intercalary cartilage (icc, Fig 9D) contacts the quadrate ventrally, supratemporal medially, and squamosal dorsally in *T*. *sulcata* and is thin and crescent shaped (Figs 2E and 9D), but this element is absent in *T*. *laevis* and *T*. *gonwouoi*. It has a rounded anterior process and a pointed posterior process that curves anterolaterally (Fig 9D).

#### Hyoid apparatus

*Trachylepis* has the typical elements of the lizard hyoid apparatus [50], including a single glossohyal and basihyal and paired hypohyals, ceratohyals, first ceratobranchials, and second ceratobranchials. The largely cartilaginous elements, such as the epibranchials, are not visible in the CT scans. The posterior regions of ceratobranchial I and ceratobranchial II were incompletely captured during scanning due to the position of the specimens. The glossohyal (ghy, Fig 9E; or lingual process) originates from the basihyal and is elongate, reaching the posterior region of the vomer in *T*. *laevis* and *T*. *sulcata* and the posterior region of the palatine in *T*. *sulcata*. It lies ventral and nearly parallel to the cultriform process. The basihyal (bhy, Fig 9E) possesses a posterior bifurcation, and three distinct elements arise from each posterior bifurcating tip. The hypohyal (hhy, Fig 9E) originates anteriorly, the first ceratobranchial (cbI, Fig 9E) originates posterolaterally, and the second ceratobranchial (cbII, Fig 9E) originates posteromedially. The ceratohyal (chy, Fig 9E) runs parallel to the first certobranchial in all three taxa. The morphology of the hyoid apparatus in *T*. *laevis* and *T*. *sulcata* is quite similar: the hypohyals and second ceratobranchials are short, similar in length, and project nearly parallel to the glossohyal, while the elongate first ceratobranchials, as well as the ceratohyals, project dorsolaterally. Each individual hyoid element in *T*. *gonwouoi* is relatively shortened, more robust, and more laterally expanded than the other two taxa.

### Neurocranium

**Fig 10:**
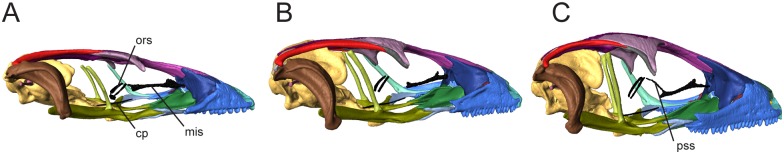
Orbitotemporal region of the neurocranium within the dermatocranium of *Trachylepis* species examined. **A**, *Trachylepis laevis*; **B**, *Trachylepis sulcata*; **C**, *Trachylepis gonwouoi*. **A–C**, lateral view. The right jugal is removed. Orbitotemporal elements are colored black. **Abbreviations**: **cp**, cultriform process; **mis**, medial interorbital septum; **ors**, paired oribtosphenoids; **pss**, planum supraseptale. Scale bars = 1 mm.

The neurocranium is divided into an orbitotemporal region, represented by paired orbitosphenoids, and an otooccipital region, which is formed by the sphenoid, basioccipital, supraoccipital, prootic, and otooccipital (opisthotic + exoccipital, [58]). These elements form a fused structure. The paired oribtosphenoids (ors, Fig 10A–10C) are present in all three taxa and are dorsal to the medial segment of the cultriform process of the sphenoid. A medial interorbital septum (mis, Fig 10A–10C) lies dorsal to the anterior extent of the cultriform process and extends anteriorly to the space between the prefrontals. The planum supraseptale (pss, Fig 10C) is visible only in *T*. *gonwouoi* and nearly connects the medial interorbital septum to the paired orbitosphenoids.

**Fig 11:**
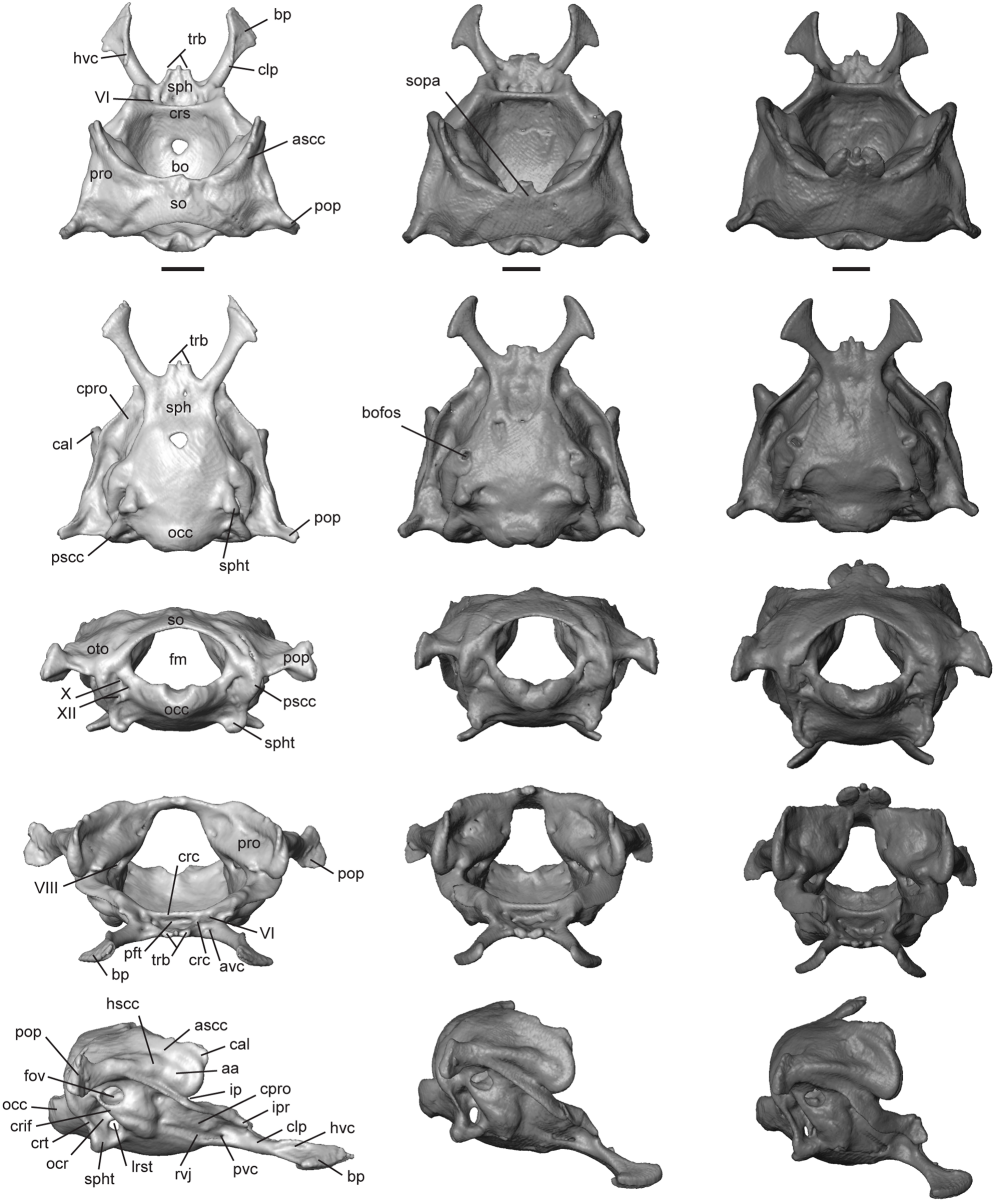
Isolated otooccipital region of the neurocranium of *Trachylepis* species examined. Light gray elements = *T*. *laevis*, medium gray elements = *T*. *sulcata*, and dark gray elements = *T*. *gonwouoi*. Otooccipital region in dorsal (top), ventral (second row), posterior (third row), anterior (fourth row), and lateral (bottom) views. **Abbreviations**: **aa**, anterior ampulla; **ascc**, anterior semicircular canal; **avc**, anterior opening of the vidian canal; **bo**; basioccipital; **bofos**, basioccipital fossa; **bp**, basipterygoid process; **cal**, crista alaris; **clp**, clinoid process; **cpro**, crista prootica; **crc**, carotid canal; **crif**, crista interfenestralis **crs**, crista sellae; **fm**, foramen magnum; **fov**, fenestra ovalis; **hscc**, horizontal semicircular canal; **hvc**, lateral head vein; **ip**, incisura prootica; **ipr**, inferior process of the prootic; **lrst**; lateral opening of recessus scalae tympani; **occ**, occipital condyle; **ocr**, occipital recess; **oto**, otooccipital; **pft**, fossa for the pituitary gland; **pop**, paroccipital process; **pro**, prootic; **pscc**, posterior semicircular canal; **pvc**, posterior opening of the vidian canal; **rvj**, recesus vena jugularis; **so**, supraoccipital; **sopa**, processus ascendens of the supraoccipital; **sph**, sphenoid; **spht**, sphenooccipital tubercle; **trb**, trabecula; **VI**, CN VI; **VIII**, CN VIII; **X**, CN X; **XII**, CN XII. Scale bars = 1 mm.

The otooccipital region forms the basicranium, a solid structure formed by the fusion of three medial unpaired elements (sphenoid, basioccipital, and supraoccipital) and two lateral paired elements (prootic and otooccipital). This compound structure is slightly covered by the parietals dorsally, and articulates with the quadrate, supratemporal, stapes, pterygoids, and the atlas. The fusion of the sphenoid and basioccipital is complete. The brain space is cone-shaped, largely due to the internally sloping walls formed by the prootics and supraoccipital (Fig 11). The brain cavity has a greater height than width in *T*. *sulcata* and *T*. *gonwouoi* (Fig 11), and the basioccipital and sphenoid form a concave ventral surface. In posterior view of the skull, the foramen magnum (fm, Fig 11) is large and roughly circular in shape. It is bounded by the supraoccipital dorsally, otooccipitals ventrolaterally, and basioccipital ventromedially (Fig 11). The occipital condyle is single. It is formed by the otooccipitals (exoccipitals) laterally and basioccipital medially. The inner ear cavities are complex and bounded by the prootic, supraoccipital, and otooccipital. The basicranium of *T*. *laevis* is markedly flattened in shape compared to the other two taxa and the brain cavity has a greater width than height. (Fig 11).

#### Sphenoid

This is a compound bone resulting from the fusion of the basisphenoid and the dermatocranial parasphenoid. It contacts the prootic dorsally, basioccipital posteriorly, and participates in the synovial palatobasal articulation with the pterygoids [57]. Anteriorly, the sphenoid (sph, Fig 11) bears paired trabeculae (trb, Fig 11), from which a cartilaginous rod or cultriform process (cp, Fig 10A–10C, or trabecula comunis) originates. The cultriform process extends to the anterior extent of the pterygoids in all three taxa. The trabeculae are short and rounded in ventral view, and a medial, spine-like process is present in *T*. *laevis* and *T*. *gonwouoi*, separating the two trabeculae (Fig 11). The basipterygoid processes (bp, Fig 11) are oriented anterolaterally, with expanded distal facets that articulate with the basipterygoid process of the pterygoid. The basipterygoid process of *T*. *laevis* is elongated and is 34% of the total neurocranium length, whereas the basipterygoid process is only 25% of total neurocranium length in *T*. *sulcata* and *T*. *gonwouoi*. Posteriorly, the sphenoid contacts the basioccpital with a median tab and a triangular lateral lappet on each side [42], as seen in ventral view in *T*. *sulcata* and *T*. *gonwouoi* (Fig 11). The sphenoid expands anteriorly to form the crista sellae (crs, Fig 11, or dorsum sellae), which is the posterior border of the deep, wide fossa for the pituitary gland (pft, Fig 11, or sella turcica). This fossa is larger and more laterally expanded in *T*. *gonwouoi* than in *T*. *laevis* or *T*. *sulcata*. Paired carotid canals (crc, Fig 11) are dorsomedial to the larger anterior opening for the vidian canal (avc, Fig 11), both of which are located within the fossa. The foramen for the abducens nerve (CN VI, Fig 11) is dorsolateral to the carotid canal and located at the base of the clinoid process. The clinoid process (clp, Fig 11, or alar process) is a dorsolateral expansion of the sphenoid and is bordered anteriorly by the crista prootica of the prootic. Laterally, the ventrolateral crest of the clinoid process, located near the base of the basipterygoid process, contains the posterior opening of the vidian canal (pvc, Fig 11, or entocarotid fossa). A posterolaterally expanded sulcus continues from this opening located ventral to the crista prootica, forming the recesus vena jugularis (rvj, Fig 11; [50]). Dorsally, the clinoid process extends anteriorly from the crista sellaris and overlaps the basipterygoid processes. A groove for the course of the lateral head vein (hvc, Fig 11) is located between these two structures [49]. The clinoid process and groove for the course of the lateral head vein is most visible in dorsal and lateral view of *T*. *laevis* (Fig 11).

#### Basioccipital

This is a ventrally convex bone that underlies the posterior region of the brain. It contacts the sphenoid anteriorly, otooccipital laterally and vertebral column (atlas) posteriorly. There is complete fusion (no suture visible) between the basioccipital and sphenoid in all three *Trachylepis* species. The basioccipitial is longer and wider than the sphenoid, excluding the basipterygoid processes. This bone forms the medial and ventral portion of the single occipital condyle. Paired lateroventral fossae are present in *T*. *sulcata* and *T*. *gonwouoi* at the border of the basioccipital and otooccipital (bofos, Fig 11), anterior to the sphenooccipital tubercle (spht, Fig 11, or basal tuber). The sphenooccipital tubercle is formed from the basioccipitial, with the otooccipital participating in the formation of only the dorsalmost extent. The tubercle projects posteroventrally in all three taxa (Fig 11), and ventral fossae are posterior to the sphenooccipital tubercles in *T*. *sulcata* and *T*. *gonwouoi*. The dorsalmost region of the basioccipital above the tubercle participates in the formation of the ventral part of the occipital recess (ocr, Fig 11)

#### Supraoccipital

The supraoccipital forms the posterodorsal margin or roof of the neurocranium. It contacts the prootics anteriorly, otooccipitals ventrally, and borders the foramen magnum dorsally. The supraoccipital has an “inverted-U” shape in anterior view (Fig 11; [9]. The space between the “inverted-U” is variable in size, being largest in *T*. *laevis* (Fig 11) and smallest in *T*. *gonwouoi* (Fig 11). In dorsal view the supraoccipital is concave posteriorly and anteriorly. An anterodorsal processus ascendens (sopa, Fig 11) is extensively ossified in *T*. *gonwouoi*, while remaining largely ligamentous in *T*. *laevis* and *T*. *sulcata*. The processus ascendens is a small medial notch in *T*. *sulcata* that does not contact the parietal (Fig 11), while the processus ascendens in *T*. *gonwouoi* is three-pronged, possessing a medial spine and two lateral bulbous projections, and contacts the parietal firmly at the parietal fossa, between the two short posteromedial processes (Fig 11). The posterolateral walls of the supraoccipitial house the dorsal portion of the otic capsules.

#### Prootic

**Fig 12:**
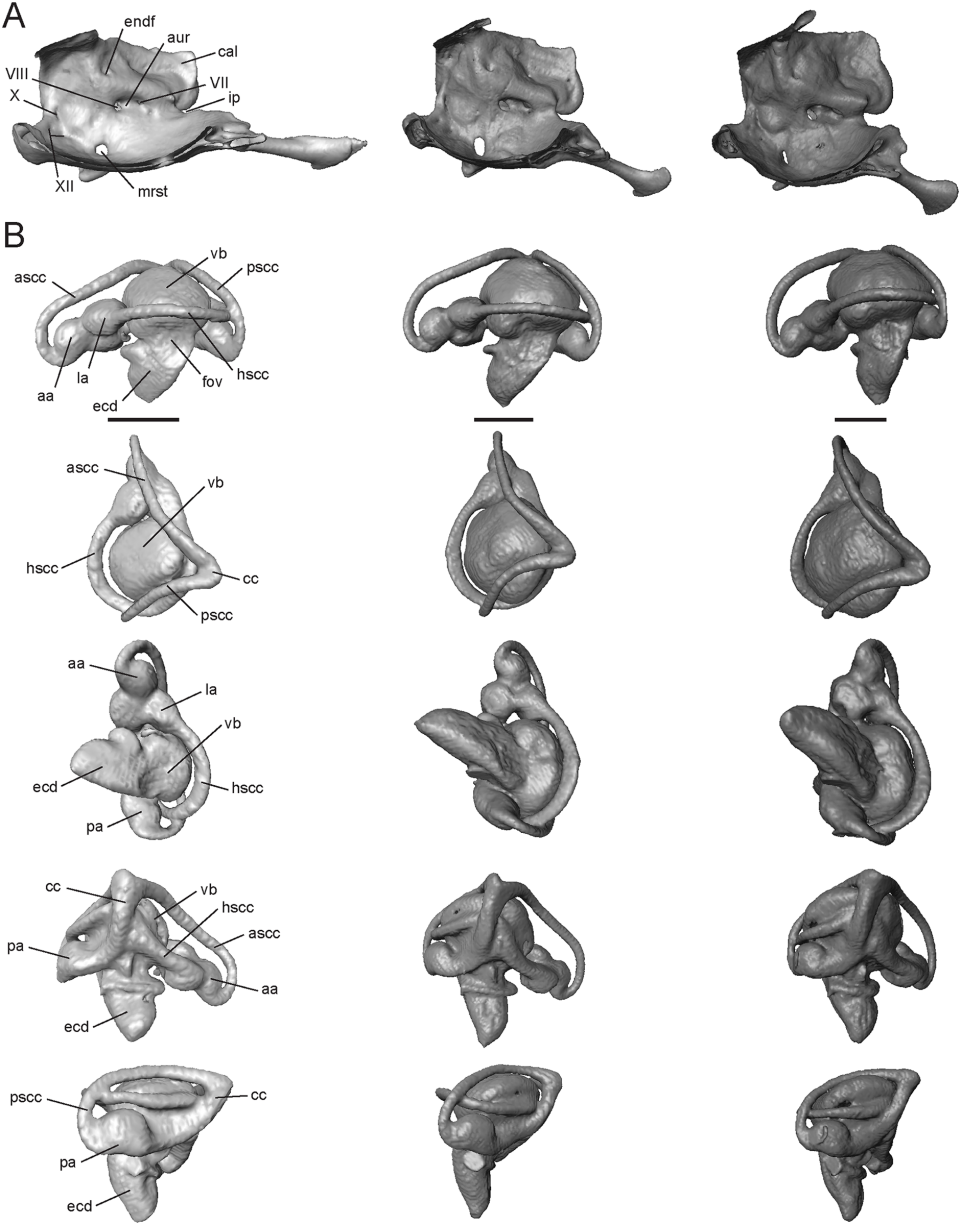
Isolated otooccipital region of the neurocranium and osseous labyrinth of *Trachylepis* species examined. Light gray elements = *T*. *laevis*, medium gray elements = *T*. *sulcata*, and dark gray elements = *T*. *gonwouoi*. **A**, Medial wall of the otooccipital region. **B**, right osseous labyrinth in lateral (top), dorsal (second row), ventral (third row), medial (fourth row), and posterior (bottom) views. **Abbreviations**: **aa**, anterior ampulla; **ascc**, anterior semicircular canal; **aur**, auditory recess of the prootica; **cal**, crista alaris; **cc**, common crus; **ecd**, endosseous cochlear duct; **endf**, endolymphatic foramen; **fov**, foramen ovale; **hscc**, horizontal semicircular canal; **ip**, incisura prootica; **la**, lateral ampulla; **mrst**, medial opening of recessus scalae tympani; **pa**, posterior ampulla; **pscc**, posterior semicircular canal; **vb**, vestibule; **VII**, CN VII; **VIII**, CN VIII; **X**, CN X; **XII**, CN XII. Scale bars = 1 mm.

This triradiate bone forms the anterodorsal part of the basicranium. It contacts the sphenoid anteroventrally, supraoccipital posterodorsally, otooccipital posteroventrally, and stapes posteriorly. The lateral and anterior regions of the prootic are partially roofed by the posterior processes of the parietal (Fig 11). The prootic houses the anterior regions of the inner ear, including: the anterior and horizontal semicircular canals, their ampullae, and the anterior portion of the vestibule. The most prominent feature of the prootic is the anterodorsally extended crista alaris (cal, Fig 11, or alar process), which bears the enlarged tracks of the anterior and horizontal semicircular canals (ascc and hscc, Fig 11; [59]). The crista alaris does not contact the epipterygoid or the decensus parietalis process in the three *Trachylepis* species examined. Posteroventral to the crista alaris, the anterior semicircular canal (ascc, Fig 11) curves downward and forms a loop, terminating at the bulging anterior ampulla (aa, Fig 11). Anteroventral to the anterior ampulla is the deep notch of the incisura prootica (ip, Fig 11), which is typically associated with the exit point for the trigeminal nerve (CN V). There is no trigeminal foramen, however, and this may suggest that cranial nerve V does not pass through the braincase, but rather flanks it medially [51]. The horizontal canal (hscc, Fig 11) continues from the anterior ampulla and extends posteriorly into the otooccipital. Ventrolateral to the incisura prootica, the crista prootica (cpro, Fig 11) contacts the otooccipital posteriorly, forming the dorsal and anterior margin of the fenestra ovalis (fov, Fig 11), and extends anterolaterally to the inferior process of the prootic (ipr, Fig 11), which contacts the clinoid process of the sphenoid. Medially, the internal openings of the foramina for the facial (CN VII, Fig 12A) and auditory (CN VIII 12A) nerves are present within the auditory recess of the prootica (aur, Fig 12A), posterior to the incisura prootica. An endolymphatic foramen (endf, Fig 12A) is dorsal to the auditory recess.

#### Otooccipital

The otooccipital is composed of the fused exoccipitial and opisthotic. It forms the lateral border of the foramen magnum and the posterolateral wall of the otic capsule, and it contacts the prootic anteriorly, basioccipital ventromedially, supraoccipital dorsally, and the quadrate, supratemporal, and stapes laterally. The horizontal semicircular canal (hscc, Fig 11) is partially housed within the otooccipital and runs laterally between its intersection with the posterior semicircular canal and the prootic. The occipital recess (ocr, Fig 11; or recessus scalae tympani) is elliptical and ventral to the fenestra ovalis. It is surrounded by the otooccipital with no participation of the basioccipital. Within the occipital recess, there is one large foramen, the lateral opening of recessus scalae tympani (lrst, Fig 11; or foramen rotundum). The medial opening of recessus scalae tympani (mrst, Fig 12A; or perilymphatic foramen) is ventral to the internal openings of the foramina for CN VII and VIII. The posterior border of the occipital recess is formed by a vertical crest, the crista tuberalis (crt, Fig 11). The crista tuberalis extends ventrally to form a V-shaped sphenooccipital tubercle (spht, Fig 11), which anteriorly borders the basioccipital. The anterior border of the occipital recess is formed by an anteroventrally directed crest, the crista interfenestralis (crif, Fig 11), which forms the posterior border of the fenestra ovalis. In posterior view of the neurocranium (Fig 11), three large foramina are present in the otooccipital region that are lateral to the formamen magnum. The posterolaterally directed vagus foramen (CN X, Fig 11) lies dorsal to two ventrolaterally directed hypoglossal foramina (CN XII, Fig 11). The internal opening of vagus nerve is located on the medial wall of the foramen magnum and is pinched into a lateral slit (X, Fig 12A), and the small circular internal openings of CN XII are near the lateral edge of the occipital condyle (XII, Fig 12A). The paroccipital process (pop, Fig 11) is positioned posterodorsal to the fenestra ovalis and extends posterolaterally to contact the posteromedial surface of the supratemporal and the cephalic condyle of the quadrate. The posterior processes of the parietal approach, but do not come in direct contact, with the paroccipital processes. The paroccipital process of *T*. *laevis* is elongated compared to *T*. *sulcata* and *T*. *gonwouoi*.

#### Osseous labyrinth

The osseous labyrinth (Fig 12B, or skeletal labyrinth, inner ear endocast), which corresponds to inner ear (membranous labyrinth) anatomy, is characterized by three semicircular canals (anterior, ascc; posterior, pscc; horizontal, hscc; Fig 12B), which are variably spaced from the vestibule (vb, Fig 12B) in the *Trachylepis* taxa examined. The horizontal semicircular canal is situated closest to the vestibule in *T*. *gonwouoi* and most distantly in *T*. *laevis*. The anterior semicircular canal is curved and highly arched in *T*. *sulcata* and *T*. *gonwouoi*, but is compressed in *T*. *laevis* (Fig 12B). The arc of the posterior semicircular canal, as well as the posterior region of the vestibule, is similarly compact in *T*. *laevis* compared to the other two species (Fig 12B). The anterior ampulla (aa, Fig 12B), lateral ampulla (la, Fig 12B), and posterior ampulla (pa, Fig 12B) are well developed in all three taxa. The anterior semicircular canal and posterior semicircular canal meet dorsomedially and form the common crus (cc, Fig 12B), which enters the ventricle medially. The body of the inner ear is divided into the vestibule (vb, Fig 12B), which corresponds to the dorsal bulbous structure where the semicircular canals converge, and the endosseous cochlear duct (ecd, Fig 12B, or cochlear recess), which is ventral to the ventricle [60]. The foramen ovale (fov, Fig 12B) opens to the lateral wall of the endosseous cochlear duct, which is anteroventrally elongated in these three taxa, as well as in other skinks [60]. The endosseous cochlear duct of *T*. *laevis* is strongly curved medially (Fig 12B). An otic sac is not visible.

### Mandibular Bones

#### Angular

**Fig 13:**
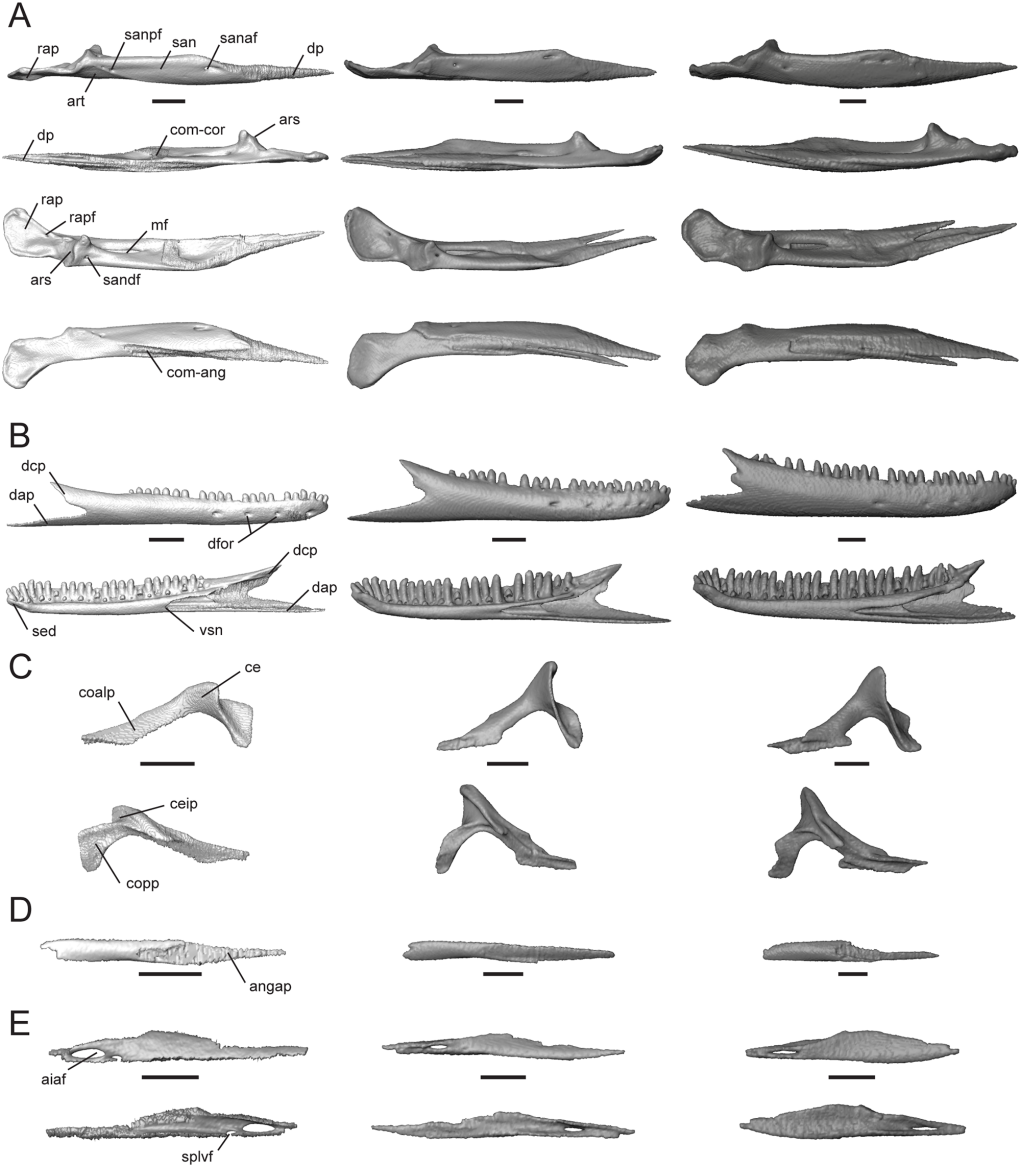
Isolated compound bone, dentary, coronoid, angular, and splenial of *Trachylepis* species examined. Light gray elements = *T*. *laevis*, medium gray elements = *T*. *sulcata*, and dark gray elements = *T*. *gonwouoi*. **A**, right compound bone in lateral (top), medial (second row), dorsal (third row), and ventral (bottom) views. **B**, right dentary in lateral (top) and labial (bottom) views. **C**, right coronoid in medial (top) and lateral (bottom) views. **D**, right angular in ventral view. **E**, right splenial in medial (top) and lateral (bottom) views. **Abbreviations**: **aiaf**, anterior inferior alveolar foramen; **angap**, anterior process of the angular; **ars**, articular surface; **art**, articular; **ce**, coronoid eminence; **ceip**; coronoid eminence insertion point; **coalp**, anterolateral process of the coronoid; **com-ang**, angular facet of the compound bone; **com-cor**, coronoid facet of the compound bone; **copp**, posterior process of the coronoid; **dap**, angular process of the dentary; **dcp**, coronoid process of the dentary; **dfor**, mental foramina; **dp**, dentary process of the compound bone; **mf**, mandibular fenestra; **rap**, retroarticular process; **rapf**, retroarticular process foramen; **san**, surangular; **sanaf**, anterior foramen of the surangular; **sandf**, dorsal foramen of the surangular; **sanpf**, posterior foramen of the surangular; **sed**; symphyseal edge; **splvf**, ventral foramen of the splenial; **vsn**, v-shaped notch of Meckelian canal. Scale bars = 1 mm.

This bone contacts the compound bone dorsally, dentary anterolaterally, and splenial anteromedially. In ventral view of the lower jaw (Fig 3C), the angular is visible overlapping the ventral surface of the compound bone and being overlapped anterolaterally by the dentary and anteromedially by the splenial. The angular of *T*. *laevis* is reduced compared to the other two taxa, while the angular of *T*. *gonwouoi* has an elongate, thin anterior process (angap, Fig 13D) that is nearly entirely overlapped by the dentary and splenial.

#### Articular and surangular

The articular and surangular are nearly separate in *T*. *sulcata* and *T*. *gonwouoi*, while these two elements are fused in *T*. *laevis* (S4 Fig). The surangular (san, Fig 13A) is a large bone that overlies the articular (art, Fig 13A). The surangular contains a posterior foramen (sanpf, Fig 13A) and anterior foramen (sanaf, Fig 13A) laterally [20], as well as the mandibular fossa (mf, Fig 13A) dorsally. Anteriorly, there is a dentary process (dp, Fig 13A) that surpasses the length of the anterolateral process of the coronoid and contacts the splenial and dentary. The dentary process is much broader and bifurcated in *T*. *sulcata* and *T*. *gonwouoi*. Ventrally, a facet is present for the angular (com-angular, Fig 13A). The articular forms the posteroventral region of the jaw and participates in craniomandibular articulation. The articular surface (ars, Fig 13A) bears a medial ridge and two depressions that accommodate the concave mandibular condyle of the quadrate. Posterior to the articular surface, the retroarticular process (rap, Fig 13A) is flattened. The retroarticular process of *T*. *laevis* is smaller than the process of *T*. *sulcata* and *T*. *gonwouoi*.

#### Coronoid

This bone contacts the dentary anteriorly, compound bone ventrally, and splenial anteromedially. The coronoid eminence (ce, Fig 13C) is laterally compressed, projects posterodorsally, and is greatly reduced in *T*. *laevis* compared to *T*. *sulcata* and *T*. *gonwouoi*. In lateral view, the insertion point for the jaw muscle adductor mandibulae externus medialis is visible on the eminence (ceip, Fig 13C; [50]). The surface area available for muscle attachment is much greater in *T*. *sulcata* and *T*. *gonwouoi*. The anterolateral process of the coronoid (coalp, Fig 13C) extends onto the labial side of the jaw, contacting the dentary and splenial, as well as overlaying the anterior process of the compound bone. The anterolateral process of *T*. *laevis* is more anteriorly extended than in the other two taxa. The lateral and medial outline of the coronoid is sickle-shaped (Fig 13C), due to the elongate anterolateral process and a highly arced coronoid eminence + posterior process (copp, Fig 13C). The posterior process extends ventrally onto the labial side of the jaw, anterior to the mandibular fossa. *Trachylepis laevis* possesses a relatively broadened and shortened posterior process compared to *T*. *sulcata* and *T*. *gonwouoi*.

#### Dentary

The dentary is a tubular bone that contacts the coronoid and compound bone posteriorly, splenial medially, and angular ventrally. It bears 24 tooth loci and 21 teeth in *T*. *laevis*, 24 tooth loci and 22 teeth in *T*. *sulcata*, and 31 tooth loci and 26 teeth in *T*. *gonwouoi* (Fig 13B). These teeth are isodont, cylindrical, and pleurodont with rounded crowns in *T*. *laevis*, whereas *T*. *sulcata* and *T*. *gonwouoi* possess slightly more pointed crowns. The lateral side of the dentary is slightly rounded and has four mental formina in *T*. *laevis* and *T*. *gonwouoi*, but six mental formina in *T*. *sulcata* (dfor, Fig 13B). Posteriorly, the dentary is bifurcated into coronoid and angular processes (dcp and dap respectively, Fig 13B). The shorter coronoid process abuts the suture of the coronoid and compound bone, whereas the longer angular process contacts the compound bone, splenial, and angular. The coronoid process supports no teeth or tooth loci in *T*. *laevis*, but does support teeth in *T*. *sulcata* and *T*. *gonwouoi*. The angular process is much longer than the cornoid process in *T*. *laevis* and *T*. *sulcata*, but these two processes are nearly the same length in *T*. *gonwouoi*. Anteriorly, the symphyseal edge (sed, Fig 13B) is obliquely oriented. The Meckelian canal is completely obliterated by the dentary and splenial; however, a v-shaped notch of the canal (vsn, Fig 13B) is visible where the splenial overlaps the dentary. The whole of the dentary, as well as the coronoid and angular processes, are more narrow and elongate in *T*. *laevis* compared to the other two taxa examined.

#### Splenial

This lingual jaw bone contacts the dentary anteriorly, compound bone posterodorsally, coronoid dorsally, and angular posteriorly. A large, anterior inferior alveolar foramen (aiaf, Fig 13E) is present in all three taxa, while a smaller, ventral foramen for the anterior mylohyoid nerve (splvf, Fig 13E) is present only in *T*. *laevis* [20]. The anterior process overlaps the dentary, while the posterior process overlaps the angular. The splenial is relatively narrow and elongate in *T*. *laevis* and *T*. *sulcata* compared to the broad and short structure in *T*. *gonwouoi*.

## Discussion

Our results provide the first detailed anatomical atlas of the adult mabuyid skull described and illustrated element-by-element in its entirety using micro-computed tomography. All three *Trachylepis* taxa examined possess nine premaxillary tooth loci and an obliterated Meckelian canal, two postulated morphological diagnoses of Mabuyidae [11]. Current taxonomic hypotheses place *T*. *laevis* and *T*. *sulcata* in the same southern and southeastern African clade, whereas *T*. *gonwouoi* is allied to *T*. *affinis* and nested within the western & central African clade [61; Bauer et al., unpublished data]. There is considerable morphological variation within these three taxa and the other *Trachylepis* previously examined (S1 Appendix), which illustrates the uncertainty in identifying potential morphological characters for this lineage. The most meaningful comparison of our data is with the description of the reconstructed adult skull of *T*. *capensis* by Skinner [20]. *Trachylepis capensis* is a terrestrial species found in clearings and open sandy areas [62] and is a member of the same large clade of southern African *Trachylepis* as *T*. *sulcata* and *T*. *laevis* (Bauer et al., unpublished data). The description of its skull is comparable to that of *T*. *gonwouoi* in overall shape, as both species have deeper, more heavily ossified skulls than *T*. *laevis* and *T*. *sulcata*. This similarity in skull shape may be the result of ecological convergence, as *T*. *gonwouoi* is also considered a terrestrial species, often found in leaf litter and on tree trunks [61]. Alternatively, if the terrestrial morphology represents the ancestral body form in *Trachylepis*, then similarity in skull proportions in *T*. *capensis* and *T*. *gonwouoi* may be plesiomorphic and represent a lack of divergence. The shape, arrangement, and relative proportions of the individual roofing bones (including the nasals, frontal, parietal, squamosals, supratemporals) and circumorbital bones (including the prefrontals, lacrimals, jugals, postfrontals, postorbitals) are more similar between *T*. *capensis* and *T*. *sulcata* than *T*. *gonwouoi*, however, which may be due to their shared clade membership. For example, the squamosal and jugal nearly contact one another in *T*. *sulcata* and *T*. *capensis*, but these two elements are broadly separated in *T*. *gonwouoi*. The roofing bones are narrower, the suborbital fenestrae are smaller, there are fewer maxillary and dentary tooth loci and teeth, and the jugal is more strongly recumbent in both *T*. *sulcata* and *T*. capensis than in *T*. *gonwouoi*. *Trachylepis capensis* and *T*. *gonwouoi* do share similarly angled nasal processes of the premaxilla, deep posterior processes of the maxilla, short basipterygoid processes, and the presence of pterygoid teeth. *Trachylepis capensis* is distinct from both *T*. *sulcata* and *T*. *gonwouoi* in possessing posterior fusion of the nasals, wide separation between the palatines, and a deep inflection of the pterygoid in lateral view [20]. The remaining features of the *T*. *capensis* skull, including the neurocranium, are similar to *T*. *gonwouoi*.

The head of *Trachylepis laeivs* is highly dorsoventrally depressed compared to *T*. *sulcata* and *T*. *gonwouoi* (Fig 2B), allowing this species to take refuge in narrow rock crevices [47, 62]. The skull of *Trachylepis sulcata* is also flattened compared to *T*. *gonwouoi*, and it is similarily considered a rupicolous species [62, 63]. *Trachylepis laevis* is distinct in that it lacks a postorbital bone, lacks an ossified processus ascendens of the supraoccipital, lacks a bifurication of the dentary process of the surangular, possesses a fused premaxilla, and possesses a fused articular and surangular. Both *T*. *laevis* and *T*. *sulcata* lack pterygoid teeth, while these structures are present in *T*. *gonwouoi*, *T*. *capensis* [20], *T*. *atlantica* [43], and *T*. *maculilabris* [44]. The jugal does not contact the lacrimal or prefrontal in *T*. *laevis* or *T*. *sulcata*, but does in *T*. *gonwouoi* and *T*. *capensis*. The presence of the postorbital is considered an ancestral trait for lygosomoid skinks and its absence indicates either a loss of the bone or fusion with the postfrontal [32]. *Trachylepis laevis* is the only mabuyid skink identified to lack a postorbital except for the extinct giant skink *Chioninia coctei* [37]. A ventral ridge is present on the postfrontal of *T*. *laevis* (Fig 5C), and this crest may be a fusion point between the postorbital and postfrontal during development; however, further examination of an ontogenetic series is required to verify the loss or fusion of this element. Most mabuyid skinks appear to possess pterygoid teeth, despite their absence in *T*. *laevis* and *T*. *sulcata*; although Greer [36] reported intraspecific variation in their presence or absence within lygosomoid species.

Dorsoventral depression of the skull has been documented in other crevice-dwelling skinks (*Egernia cygnitos* and *E*. *epsisolus*, [64]; *Cryptoblepharus* sp., [65]); however the depressed skulls of these species are not as extreme as in *T*. *laevis*. Widespead convergence in depressed head depth has been identified across rock-dwelling lizards more broadly as well, including members of the genus *Platysaurus* (Cordylidae), *Petrosaurus thalassinus* (Phrynosomatidae), *Anolis bartschi* (Polychrotidae), and *Tropidurus semitaeniatus* (Tropiduridae) [66]. Although the relationship between crevice-dwelling habits and dorsoventral depression of head height has been previously recognized in lizards, the osteological modifications that occur in the skull have yet to be identified.

*Trachylepis laevis* possesses a number of highly modified skull characters that we hypothesize are morphological adaptations related to its extreme rupicolous habits, including a flattened skull roof (resulting in a low arc of the skull), thin skull roof bones, many strongly recumbent elements, a dorsoventrally compressed neurocranium, reduced height of the dorsal process of the maxilla, elongate posterior lower jaws, and reduced coronoid processes. The strongly recumbent elements include all of the vertical-axis structures of the skull—the epipterygoid, quadrate, jugal, and nasal process of the premaxilla. The skull of *Trachylepis laevis* also exhibits other modifications that differ from its congeners and may be architecturally relevant to reducing cranial height, including the loss of the postorbital, tightly joined septomaxillae, fusion of the premaxillae, size reduction of the palatal bones, incomplete postorbital bar (jugal does not articulate with the postfrontal), shortened epipterygoid, and a completely enclosed squamosal notch of the quadrate.

The most significant functional consequence of these cranial modifications in *T*. *laevis* may be a shift in bite force and cranial kinesis performance. Previous research has demonstrated that head depth is a strong predictor of bite force strength in xenosaurids [67], lacertids [68] and iguanids [69], with head depression resulting in a diminished bite force due to shortening of the in-levers of the jaw adductor muscles. The reduced height of the coronoid processes in *T*. *laevis* also may suggest a reduction in bite force, as cornoid height corresponds to the effective lever arm for the temporalis muscle [70]. The different proportions of the tooth-bearing dentary versus the non-tooth-bearing posterior lower jaw bones between *T*. *laevis*, *T*. *sulcata*, and *T*. *gonwouoi*, as well as differences in maxilla tooth morphology, may suggest biomechanical (i.e., shifts in maximum jaw gape) and dietary divergences between these closely related taxa and requires further attention. The modifications of the postorbital bar and epipterygoid in *T*. *laevis* may correspond to increased cranial kinesis. The jugal of *T*. *laevis* is generally reduced in size and thickness, has a much narrower articulation with the posterior process of the maxilla, and does not directly contact the skull roof, resulting in an incomplete postorbital bar (potentially due to the absence of the postorbital). The complete loss of the postorbital bar has occurred within gekkotans and varanids, and both of these groups are reported to have enhanced cranial, and especially mesokinetic (the joint between the parietal and frontal), capabilities [71–73]. The reduction in height of the epipterygoid may further permit mesokinesis. Arnold [74] suggested that crevice-dwelling scincomorph lizards may have evolved enhanced cranial kinesis capabilities (specifically mesokinesis) to allow for further retreat into narrow fissures when pursued by predators. This hypothesis has yet to be supported with empircal data; however, we have identified patterns of similarity in *Trachylepis* that Arnold [74] described in lacertids: crevice-dwelling species possess flat, thin skulls that have greater potential for mobility, while wide-ranging species possess deeper, more heavily ossified skulls. Enlargement of the nasal openings, enlargement of the suborbital fenestrae, and reduction of the skull osteoderms are three modifications Arnold [74] identified in crevice-dwelling lacertids that were not found in *Trachlyepis laevis* or *T*. *sulcata*.

There is a need for increased sample sizes in squamate morphological research [56, 75], particularly in computed tomography studies [76], to further characterize the intraspecific variation of the lizard skull and to identify patterns of variation and phylogenetically informative characters of disarticulated skeletal elements. We recognize the need for further intraspecific and interspecfic morphological sampling of *Trachylepis* species, as well as other mabuyid genera, to fully comphrehend the phenotypic diversity and evolution of this speciose lineage of skinks. We hope our study, which is the first element-by-element description of a scincid lizard using CT technology, will act as an anatomical baseline for future work in cranial osteology of skinks. Our results indicate that there is much osteological variation present within this group, including the loss of structures, fusion of elements, and shape and scaling modifications across nearly every cranial bone. *Trachylepis laevis* possesses a highly dosoventrally depressed skull, and we have identified several osteological modifications that have evolved due to this species rupicolous habits. Furthermore, these modifications may possess functional consequences related to bite force and cranial kinesis capabilities, and we suggest these taxa would provide an ideal system to investigate how head morphology influences cranial biomechanic capabilities and ecology.

## Supporting information

S1 Appendix Survey of scincid morphological studies that depict articulated skulls, disarticulated elements, or histological sections. It is noted parenthetically which view(s) are provided and whether figure labels are included. Dagger (†) denotes extinct taxa and asterisk (*) denotes revised taxonomy (i.e., the species name here reflects current taxonomic hypotheses, rather than the taxonomy used in the referenced work).
(PDF)

S1 Fig Length and angle measurements recorded using the measure tool in Avizo. These measurements include skull length, skull width, thickness of the frontal table, angle of the snout, quadrate angle, epipterygoid angle, and angle of the medial process of the frontal.
(TIF)

S2 Fig Transverse cross-section tomograms of the snout of *Trachylepis laevis* (left), *T*. *sulcata* (center), and *T*. *gonwouoi* (right). The premaxilla is fused in *T*. *laevis*, while midline sutures are visible in *T*. *sulcata* and *T*. *gonwouoi*. **Abbreviations**: **mx**, maxilla; **pmx**, premaxilla; **v**, vomer.
(TIF)

S3 Fig Coronal cross-section tomogram of the snout of *Trachylepis laevis*. The septomaxilla is fused at the midline. **Abbreviations**: **d**, dentary; **mx**, maxilla; **smx**, septomaxilla; **v**, vomer.
(TIF)

S4 Fig Coronal cross-section tomograms of the mandible anterior to the mandibular fossa (A) and at the mandibular fossa (B) in *Trachylepis laevis* (left), *T*. *sulcata* (center), and *T*. *gonwouoi* (right). The articular and surangular are fused in *T*. *laevis*, while they are separate in *T*. *sulcata* and *T*. *gonwouoi*. **Abbreviations**: **ang**, angular; **art**, articular; **mf**, mandibular fossa; **pt**, pterygoid; **san**, surangular.
(TIF)
