# Supplementary material for: Comparative skull anatomy of terrestrial and crevice-dwelling Trachylepis skinks (Squamata: Scincidae) with a survey of resources in scincid cranial osteology
Source: PLoS One. 2017 Sep 13;12(9):e0184414. doi: 10.1371/journal.pone.0184414 (PMC5597209; doi:10.1371/journal.pone.0184414)
Supplement: S1 Appendix — It is noted parenthetically which view(s) are provided and whether figure labels are included. Dagger (†) denotes extinct taxa and asterisk (*) denotes revised taxonomy (i.e., the species name here reflects current taxonomic hypotheses, rather than the taxonomy used in the referenced work). (PDF) [file pone.0184414.s001.pdf]

S1 Appendix. Survey of scincid morphological studies that depict articulated skulls, disarticulated elements, or histological sections. It is noted parenthetically which view(s) are provided and whether figure labels are included. Dagger (†) denotes extinct taxa and asterisk (*) denotes revised taxonomy (i.e., the species name here reflects current taxonomic hypotheses, rather than the taxonomy used in the referenced work).

**Species**	**Cranial Illustrations**	**Reference**
ACONTIDAE		
*Acontias aurantiacus**	articulated skull (posterolateral, posteroventral; unlabeled) and mandible (lingual, unlabeled), articulated posterior portion of skull (ventral, lateral; labeled), histological sections	1
*Acontias aurantiacus**	articulated skull (lateral; labeled)	2
*Acontias aurantiacus**	articulated skull (lateral; labeled), mandible (lateral; labeled)	3
*Acontias aurantiacus**	articulated skull (lateral; labeled)	4 (modified from 3)
Acontias breviceps	articulated skull (ventral; unlabeled)	5
*Acontias cregoi**	articulated skull (dorsal, ventral, lateral; labeled)	2
*Acontias cregoi**	aticulated skull (lateral; labeled), mandible (lateral; labeled)	3
*Acontias cregoi**	aticulated skull (lateral; labeled)	4 (modified from 3)
Acontias gracilicauda	articulated skull (dorsal; labeled)	2
*Acontias kgalagadi**	articulated skull (ventral; labeled)	6
*Acontias lineatus**	articulated skull (dorsal, ventral, lateral; labeled), histological sections	2
*Acontias lineatus**	articulated skull (lateral; labeled), mandible (lateral; labeled)	3
*Acontias lineatus**	articulated skull (lateral; labeled)	4 (modified from 3)
*Acontias lineatus**	articulated skull (dorsal, ventral, lateral; labeled)	123 (modified from 2)
Acontias meleagris	histological sections	7
Acontias meleagris	mandible (lateral; labeled)	3
Acontias meleagris	chondrocranium (lateral, dorsal; labeled), early dermatocranium (lateral, ventral; labeled)	8
Acontias meleagris	hyoid (dorsal, ventral, lateral; labeled), inner ear (dorsal, ventral, lateral; labeled), mandible (lingual, labial; labeled)	9
Acontias meleagris	articulated skull (ventral; unlabeled)	5
Acontias meleagris	articulated skull (dorsal, ventral, lateral, posterior; labeled)	2
Acontias percivali	articulated skull (dorsal; labeled)	2
Acontias percivali	mandible (lateral; labeled)	3
Acontias percivali	articulated skull (ventral; labeled)	10

Acontias percivali	articulated skull (dorsal, lateral; unlabeled)	11
Acontias plumbeus	articulated skull (dorsal, ventral, lateral, posterior; labeled)	2
Acontias plumbeus	articulated skull (ventral; labeled)	6
Acontias plumbeus	articulated skull (lateral; labeled)	124 (modified from 2)
Acontias plumbeus	mandible (lingual; labeled)	12
*Acontias rieppeli**	articulated skull (dorsal, ventral, lateral; labeled), mandible (lateral; labeled)	3
Typhlosaurus braini	articulated skull (lateral; labeled)	2
Typhlosaurus braini	articulated skull (lateral; labeled)	3
Typhlosaurus braini	articulated skull (lateral; labeled)	4 (modified from 3)
Typhlosaurus vermis	articulated skull (lateral; labeled)	2
Typhlosaurus vermis	articulated skull (lateral; labeled)	3
Typhlosaurus vermis	articulated skull (lateral; labeled)	4 (modified from 3)
EGERNIIDAE		
Aethesia frangens†	mandible (lateral, lingual, dorsal; labeled)	12
Bellatorias sp.	dentary (lingual; labeled)	13
Corucia zebrata	articulated skull (dorsal, ventral; unlabeled), mandible (lingual; unlabeled)	14
Corucia zebrata	mandible (lingual; labeled)	12
Cyclodomorphus branchialis	coronoid region of mandible (lateral; unlabeled), articulated fronto-nasal region (dorsal; labeled), articulated jugal region (lateral; labeled)	15
*Cyclodomorphus casuarinae**	articulated palatal bones (ventral; unlabeled)	16
*Cyclodomorphus casuarinae**	articulated skull (oblique view of palatal region; unlabeled)	17
Cyclodomorphus casuarinae	coronoid region of mandible (lateral; unlabeled), articulated fronto-nasal region (dorsal; labeled), interorbital bones (dorsal; labeled), articulated jugal region (lateral; labeled), articulated palatal bones (ventral; labeled)	15
*Cyclodomorphus gerrardii**	articulated palatal bones (ventral; unlabeled)	16
*Cyclodomorphus gerrardii**	articulated skull (lateral; unlabeled), mandible (lingual; unlabeled)	18
Cyclodomorphus gerrardii	mandible (lingual; unlabeled)	19
Cyclodomorphus gerrardii	articulated palatal bones (ventral; labeled), articulated jugal region (lateral; labeled), coronoid region of mandible (lateral; unlabeled), articulated fronto-nasal region (dorsal; labeled	15
Cyclodomorphus gerrardii	articulated palatal bones (ventral; unlabeled)	20
Cyclodomorphus maximus	articulated fronto-nasal region (dorsal; labeled)	15
Egernia cunninghami	articulated palatal bones (ventral; unlabeled)	16
Egernia cunninghami	articulated palatal bones (ventral; labeled)	21

Egernia cunninghami	mandible (lingual; unlabeled)	19
Egernia cunninghami	coronoid region of mandible (lateral; unlabeled), articulated jugal region (lateral; labeled)	15
Egernia cygnitos	articulated skull (ventral; unlabeled)	22
Egernia depressa	articulated palatal bones (ventral; unlabeled)	16
Egernia depressa	articulated skull (ventral; unlabeled)	22
Egernia epsisolus	articulated skull (ventral; unlabeled)	22
Egernia hosmeri	maxillary fragment (lingual; unlabeled)	22
Egernia kingii	articulated palatal bones (ventral; unlabeled)	16
Egernia kintorei	articulated palatal bones (ventral; unlabeled)	16
Egernia major	articulated palatal bones (ventral; unlabeled)	16
Egernia mcpheei	articulated palatal bones (ventral)	15
*Egernia richardi**	articulated lacrimal region (dorsal; labeled)	15
Egernia saxatilis	articulated upper temporal arch (oblique; labeled)	15
Egernia stokesii	articulated palatal bones (ventral; unlabeled)	16
Egernia stokesii	articulated skull (ventral; unlabeled)	22
Egernia striolata	dentary (lateral, lingual; unlabeled)	24
Egernia sp.†	dentary (lateral, lingual; unlabeled)	24
Egernia sp.	dentary (lingual; labeled)	13
*Liopholis inornata**	articulated palatal bones (ventral; unlabeled)	16
*Liopholis modesta**	articulated skull (oblique view of palatal region; unlabeled)	17
*Liopholis modesta**	articulated interorbital bones (dorsal; labeled)	15
*Liopholis pulchra**	articulated palatal bones (ventral)	15
*Liopholis striata**	mandible (lingual; unlabeled)	25
*Liopholis striata**	articulated fronto-nasal region (dorsal; labeled)	15
*Liopholis striolata**	articulated palatal bones (ventral; unlabeled)	16
*Liopholis whitii**	articulated palatal bones (ventral; unlabeled)	16
*Liopholis whitii**	articulated skull (embryonic; dorsal, ventral, lateral; labeled)	26
Liopholis sp.	dentary (lingual; labeled)	13
*Lissolepis luctuosa**	articulated palatal bones (ventral; unlabeled)	16
Lissolepis sp.	dentary (lingual; labeled)	13
Proegernia palankarinnensis†	dentary (lingual, dorsal; unlabeled)	27
Tiliqua adelaidensis	articulated palatal bones (ventral; unlabeled)	16
Tiliqua adelaidensis	articulated skull (dorsal, ventral, lateral; labeled), mandible (lingual, lateral; unlabeled)	28
Tiliqua adelaidensis	articulated fronto-nasal region (dorsal; labeled)	15
Tiliqua adelaidensis	posterior dentary (lateral; unlabeled)	29
Tiliqua gigas	articulated palatal bones (ventral; unlabeled)	30

Tiliqua gigas	articulated palatal bones (ventral; unlabeled)	16
Tiliqua gigas	articulated interorbital bones (dorsal; labeled), articulated upper temporal arch (oblique; labeled), articulated fronto-nasal region (dorsal; labeled), articulated jugal region (lateral; labeled)	15
Tiliqua gigas	posterior dentary (lateral; unlabeled)	29
Tiliqua multifasciata	coronoid region of mandible (lateral; unlabeled), articulated fronto-nasal region (dorsal; labeled), articulated jugal region (lateral; labeled)	15
Tiliqua multifasciata	posterior dentary (lateral; unlabeled)	29
Tiliqua multifasciata	mandible (lingual; labeled)	12
Tiliqua multifasciata	dentary (lingual, dorsal; labeled)	13
Tiliqua nigrolutea	articulated skull (dorsal; unlabeled)	21
Tiliqua nigrolutea	disarticulated quadrate (posterior; unlabeled), retroarticular process of mandible (dorsal; unlabeled)	31
Tiliqua nigrolutea	coronoid region of mandible (lateral; unlabeled), articulated jugal region (lateral; labeled), articulated lacrimal region (dorsal; labeled)	15
Tiliqua nigrolutea	posterior dentary (lateral; unlabeled)	29
Tiliqua nigrolutea	dentary (lingual, dorsal; labeled)	13
Tiliqua occipitalis	articulated palatal bones (ventral; unlabeled)	16
Tiliqua occipitalis	disarticulated quadrate (posterior; unlabeled), retroarticular process of mandible (dorsal; unlabeled)	31
Tiliqua occipitalis	coronoid region of mandible (lateral; unlabeled), articulated fronto-nasal region (dorsal; labeled), articulated jugal region (lateral; labeled)	15
Tiliqua occipitalis	posterior dentary (lateral; unlabeled)	29
*Tiliqua rugosa**	articulated skull (dorsal, ventral; labeled), mandible (lingual, labial; labeled); parietal (oblique dorsal, lateral; labeled)	32
Tiliqua rugosa	articulated palatal bones (ventral; unlabeled)	16
Tiliqua rugosa	articulated skull (ventral; labeled)	21
Tiliqua rugosa	quadrate (posterior; unlabeled), retroarticular process of mandible (dorsal; unlabeled)	31
*Tiliqua rugosa**	articulated skull (dorsal, ventral, lateral; labeled), mandible (dorsal, ventral, lateral; labeled)	33
*Tiliqua rugosa**	articulated skull (lateral, ventral; unlabeled)	34
Tiliqua rugosa	coronoid region of mandible (lateral; unlabeled), articulated fronto-nasal region (dorsal; labeled), articulated jugal region (lateral; labeled)	15
Tiliqua rugosa	posterior dentary (lateral; unlabeled)	29

Tiliqua rugosa	dentary (lingual, dorsal; labeled)	13
Tiliqua scincoides	disarticulated quadrate (lateral; unlabeled), retroarticular process of mandible (dorsal; unlabeled)	31
Tiliqua scincoides	articulated skull (neonate and adult; lateral; unlabeled), mandible (neonate and adult; lingual; unlabeled).	18
Tiliqua scincoides	mandible (lingual; unlabeled)	19
Tiliqua scincoides	coronoid region of mandible (lateral; unlabeled), articulated fronto-nasal region (dorsal; labeled), articulated palatal bones (ventral, labeled), articulated jugal region (lateral; labeled)	15
Tiliqua scincoides	dentary (lingual, lateral; unlabeled)	23
Tiliqua scincoides	posterior dentary (lateral; unlabeled)	29
Tiliqua scincoides	articulated skull (ventral; labeled)	10
Tiliqua wilkinsonorum	dentary (lingual, lateral; labeled), posterior dentary (lateral; unlabeled)	29
EUGONGYLIDAE		
Ablepharus borealis†	dentary (unlabeled)	35
Ablepharus deserti	articulated palatal bones (ventral; unlabeled)	36
Ablepharus kitaibelii	articulated skull (ventral; unlabeled)	37
Ablepharus kitaibelii	articulated skull (ventral; unlabeled)	38
Ablepharus kitaibelii stepaneki	articulated palatal bones (ventral)	39
Ablepharus kitaibelii	articulated skull (ventral; labeled), histological sections	6
Ablepharus pannonicus	histological sections	40
Carlia bicarinata	articulated palatal bones (ventral; labeled)	41
Carlia bicarinata	articulated palatal bones (ventral; unlabeled)	36
Carlia longipes	articulated skull (ventral; unlabeled)	25
Carlia longipes	articulated skull (ventral; labeled)	17
Cryptoblepharus boutonii	articulated skull (ventral; unlabeled)	37
Cryptoblepharus boutonii	articulated skull (ventral; unlabeled)	38
Cryptoblepharus boutonii	articulated palatal bones (ventral; unlabeled)	36
Cryptoblepharus poecilopleurus	frontal (dorsal; unlabeled)	42
Emoia bogerti	articulated palatal bones (ventral; unlabeled)	36
Emoia cyanura	frontal (dorsal; unlabeled)	42
Emoia loyaltiensis	disarticulated elements (subset; labeled)	43
Emoia ponapea	articulated skull (dorsal, ventral; unlabeled)	44
Emoia samoense	articulated palatal bones (ventral; unlabeled)	41
Eugongylus albofasciolatus	articulated skull (ventral; labeled), mandible (lingual; unlabeled)	36
Eugongylus albofasciolatus	mandible (lingual; labeled)	12

Eugongylus rufescens	articulated palatal bones (ventral; unlabeled)	36
Eugongylus rufescens	mandible (lingual; unlabeled)	19
Eugongylus sp.†	partial mandible (lateral, lingual; unlabeled)	24
*Epibator nigrofasciolatum**	articulated skull (ventral; unlabeled)	45
Geomyersia glabra	articulated palatal bones (ventral; unlabeled)	41
Geomyersia glabra	articulated palatal bones (ventral; unlabeled)	36
*Lacertaspis reichenowi**	articulated skull (ventral; unlabeled)	45
Leiolopisma mauritiana	articulated skull (dorsal, ventral; unlabeled), mandible (lateral, lingual; unlabeled)	46
Leiolopisma telfairii	articulated palatal bones (ventral; unlabeled)	36
Leptosiaphos blochmanni	articulated palatal bones (ventral; unlabeled)	47
Leptosiaphos blochmanni	articulated palatal bones (ventral; unlabeled)	41
*Leptosiaphos meleagris**	articulated palatal bones (ventral; unlabeled)	36
Lioscincus nigrofasciatus	disarticulated elements (subset; labeled)	43
Lipinia noctua	frontal (dorsal; unlabeled)	42
Lobulia brongersmai	articulated palatal bones (ventral; labeled)	48
Lobulia elegans	articulated palatal bones (ventral; unlabeled)	36
Menetia greyii	articulated skull (ventral; unlabeled)	37
Menetia greyii	articulated skull (ventral; unlabeled)	38
Menetia greyii	articulated palatal bones (ventral; unlabeled)	36
*Morethia lineoocellata**	articulated palatal bones (ventral; labeled)	49
Morethia lineoocellata	articulated skull (ventral; unlabeled)	37
Morethia lineoocellata	articulated skull (ventral; unlabeled)	38
*Niveoscincus metallicus**	articulated palatal bones (ventral; unlabeled)	41
*Niveoscincus microlepidotus**	articulated skull (dorsal, ventral; unlabeled)	50
*Niveoscincus pretiosus**	articulated skull (ventral; unlabeled)	25
*Niveoscincus pretiosus**	articulated palatal bones (ventral; unlabeled)	50
*Niveoscincus pretiosus**	articulated palatal bones (ventral; unlabeled)	6
*Oligosoma alani**	disarticulated elements (subset; labeled)	51
*Oligosoma alani**	articulated skull (dorsal, ventral; unlabeled), disarticulated elements (subset; unlabeled)	52
*Oligosoma infrapunctatum**	disarticulated elements (subset; labeled)	51
*Oligosoma macgregori**	disarticulated elements (subset; labeled)	51
*Oligosoma northlandi**	articulated skull (dorsal, ventral; labeled), articulated mandible (lingual; unlabeled), disarticulated elements (subset; unlabeled)	52
*Oligosoma oliveri**	maxilla (medial; unlabeled)	53
*Oligosoma oliveri**	disarticulated elements (subset; labeled)	51

*Oligosoma suteri**	parietal (ventral; unlabeled), braincase (ventral; unlabeled)	53
*Oligosoma whitakeri**	mandible (anterior; unlabeled), parietal (ventral; unlabeled), braincase (ventral; unlabeled)	53
*Oligosoma whitakeri**	disarticulated elements (subset; labeled)	51
Oligosoma sp.†	disarticulated element framents (subset)	54
Panaspis africanus	articulated skull (ventral; unlabeled)	45
Panaspis cabindae	articulated skull (ventral; unlabeled)	37
Panaspis cabindae	articulated skull (ventral; unlabeled)	38
Panaspis cabindae	articulated skull (ventral; unlabeled)	55
Panaspis megalurus	articulated palatal bones (ventral)	55
*Panaspis nimbaensis**	articulated skull (ventral; unlabeled)	55
*Panaspis seydeli**	articulated palatal bones (unlabeled; ventral)	41
Panaspis seydeli	articulated skull (ventral; unlabeled)	55
*Panaspis seydeli**	articulated palatal bones (ventral; unlabeled)	36
Panaspis wahlbergi	articulated skull (ventral; unlabeled)	38
Panaspis wahlbergi	articulated skull (ventral; unlabeled)	55
Proablepharus reginae	articulated skull (ventral; unlabeled)	37
Proablepharus reginae	articulated skull (ventral; unlabeled)	38
*Pseudemoia rawlinsoni**	articulated skull (dorsal, ventral; unlabeled)	56
*Pseudemoia spenceri**	articulated palatal bones (ventral; unlabeled)	36
*Saproscincus challengeri**	articulated palatal bones (ventral; unlabeled)	36
LYGOSOMIDAE		
*Lamprolepis smaragdina**	articulated skull (dorsal, ventral, lateral; labeled), mandible (lateral, lingual; labeled)	57
Lamprolepis smaragdina	articulated palatal bones (ventral; labeled)	58
Lamprolepis sp.	articulated palatal bones (ventral; unlabeled)	59
*Leiolopisma mauritiana**†	articulated skull (dorsal, ventral; unlabeled)	60
*Lepidothyris bowringi**	articulated palatal bones (ventral; labeled)	58
*Lepidothyris fernandi**	articulated palatal bones (ventral; unlabeled)	59
*Lepidothyris punctata**	articulated palatal bones (ventral)	47
*Lygosoma albopunctatum**	articulated palatal bones (ventral; unlabeled)	59
Lygosoma bowringii	articulated palatal bones (ventral; unlabeled)	59
Lygosoma pembanum	articulated palatal bones (ventral; unlabeled)	59
Lygosoma punctatum	articulated palatal bones (ventral; unlabeled)	59
Lygosoma quadrupes	articulated palatal bones (ventral; unlabeled)	59
Lygosoma sp.	articulated skull (ventral, lateral; labeled)	61
*Mochlus afer**	articulated palatal bones (ventral; unlabeled)	59
*Mochlus afer**	articulated palatal bones (ventral; labeled), histological sections	6
*Mochlus mabuiiforme**	articulated palatal bones (ventral; unlabeled)	59

*Mochlus tanae**	articulated palatal bones (ventral; unlabeled)	59
*Mochlus vinciguerrae**	articulated palatal bones (ventral; unlabeled)	59
MABUYIDAE		
*Chioninia coctei**†	articulated skull (oblique lateral; labeled), mandible (labial; labeled), isolated tooth	62
*Chioninia coctei**†	articulated skull (dorsal, lateral; unlabeled)	63
*Chioninia coctei**†	articulated skull (dorsal, ventral; unlabeled)	60
*Chioninia coctei**†	articulated palatal bones (ventral; unlabeled)	59
*Chioninia coctei**†	articulated skull (dorsal, ventral; unlabeled), mandible (lingual; unlabeled)	14
Chioninia delalandii	articulated skull (ventral; labeled)	60
Chioninia stangeri	articulated skull (ventral; labeled)	60
Dasia olivacea	articulated palatal bones (ventral; labeled)	58
*Dasia vittata**	articulated palatal bones (ventral; labeled)	58
Dasia sp.	articulated palatal bones (ventral; unlabeled)	59
Eumecia anchietae	articulated palatal bones (unlabeled; ventral)	47
Eumecia anchietae	articulated palatal bones (ventral; unlabeled)	41
Eumecia sp.	articulated palatal bones (ventral; unlabeled)	59
*Eutropis carinata**	chondrocranium (dorsal, ventral, lateral; labeled), articulated skull (dorsal, ventral, lateral, mesial, posterior; labeled), mandible (lateral, lingual; labeled), disarticulated elements (subset; labeled), histological sections	64
*Eutropis dissimilis**	articulated palatal bones (ventral)	65
*Eutropis dissimilis**	articulated skull (lateral, posterior; labeled)	66
*Eutropis longicaudata**	articulated palatal bones (ventral; labeled)	58
*Eutropis longicaudata**	articulated skull (ventral; labeled)	59
*Eutropis multifasciata**	histological sections	57
*Eutropis multifasciata**	articulated skull (dorsal, ventral, lateral; unlabeled)	67
*Eutropis multifasciata**	coronoid region of mandible (lateral; unlabeled)	15
*Eutropis multifasciata**	articulated skull (dorsal view; labeled)	68 (modified from 67)
*Hermites aurata**	articulated skull (dorsal, ventral, lateral, posterior; labeled), mandible (lateral, lingual; labeled), disarticulated elements (subset)	69
*Hermites aurata**	chondrocranium (dorsal, vental; labeled), articulated skull (dorsal, ventral; labeled), histological sections	70
*Hermites aurata transcaucasica**	articulated skull (dorsal, ventral; unlabeled) mandible (lingual; unlabeled)	71

*Heremites vittata**	articulated skull (dorsal, ventral, lateral, posterior; labeled), mandible (lateral, lingual; labeled), disarticulated elements (subset)	69
Mabuya [sensu lato] sp.	articulated palatal bones (ventral; unlabeled)	59
Mabuya [sensu lato] sp.	articulated skull (dorsal, ventral, lateral; labeled)	72
Mabuya [sensu lato] sp.	chondrocranium (dorsal, lateral; labeled), articulated skull (dorsal, ventral; labeled)	73
*Psychosaura macrorhyncha**†	articulated skull (dorsal, ventral; unlabeled)	73
*Trachylepis atlantica**	articulated skull (dorsal, lateral, ventral; unlabeled), mandible (lateral, lingual; unlabeled), hyoid (unlabeled)	74
*Trachylepis brevicollis**	middle ear and adjacent bones (labeled)	75
*Trachylepis capensis**	articulated skull (dorsal, ventral, lateral; labeled), mandible (lateral, lingual; labeled), disarticulated elements (subset)	76
Trachylepis gonwouoi	articulated skull (dorsal, ventral, lateral, anterior, posterior; labeled), mandible (lateral, lingual, ventral; labeled), distarticulated elements (all elements; labeled)	This study
Trachylepis laevis	articulated skull (dorsal, ventral, lateral, anterior, posterior; labeled), mandible (lateral, lingual, ventral; labeled), distarticulated elements (all elements; labeled)	This study
Trachylepis maculilabris	articulated skull (dorsal, ventral, lateral; labeled)	72
*Trachylepis megalura**	histological sections	77
*Trachylepis polytropis**	articulated palatal bones (ventral; labeled)	47
*Trachylepis polytropis**	articulated palatal bones (ventral; labeled)	41
Trachylepis sulcata	articulated skull (dorsal, ventral, lateral, anterior, posterior; labeled), mandible (lateral, lingual, ventral; labeled), distarticulated elements (all elements; labeled)	This study
SCINCIDAE		
Brachymeles miriamae	articulated skull (dorsal, ventral, lateral; unlabeled)	78
Chalcides chacides	articulated skull (ventral; labeled), histological sections	6
Chalcides chacides	articulated skull (ventral; labeled), histological sections	79
Chalcides colosii	articulated skull (posterior; labeled), mandible (lingual, lateral; labeled)	79
Chalcides guentheri	articulated skull (dorsal, lateral, ventral; labeled), mandible (lateral, lingual; labeled), histological sections	80
Chalcides guentheri	articulated skull (dorsal, ventral; labeled)	81 (modified after 80)
Chalcides mauritanicus	articulated skull (posterior; labeled)	79
Chalcides mertensi	articulated skull (lateral; labeled)	79
Chalcides minutus	articulated skull (dorsal; labeled)	79
Chalcides ocellatus	chondrocranium (dorsal, ventral, lateral; labeled), articulated skull (dorsal, ventral,	82

	lateral; labeled), mandible (lateral, lingual; labeled), histological sections	
Chalcides ocellatus	articulated skull (dorsal, ventral, lateral, posterior; labeled), mandible (lingual, lateral; labeled)	83
Chalcides ocellatus	articulated skull (lateral; unlabeled)	84
Chalcides ocellatus	mandible (lateral; labeled)	3
Chalcides ocellatus	articulated skull (dorsal, ventral; labeled), mandible (lingual; labeled)	85
Chalcides ocellatus	articulated skull (dorsal, ventral, lateral; labeled), mandible (lingual, lateral; labeled)	86
Chalcides ocellatus	articulated skull (dorsal, ventral; labeled), mandible (ventral; labeled)	79
Chalcides polylepis	articulated skull (dorsal; labeled)	79
Chalcides sepsoides	articulated skull (dorsal, ventral; labeled)	79
Chalcides sexlineatus	hyoid (ventral; labeled)	79
Chalcides simonyi	articulated skull (lateral; unlabeled)	57
Chalcides simonyi	articulated skull (dorsal, lateral; unlabeled)	87
Eumeces hixonorum†	dentary (lingual; unlabeled)	88
Eumeces humilis	articulated skull (dorsal; labeled)	89
Eumeces minimus†	disarticulated frontal (dorsal, ventral; unlabeled)	90
Eumeces minimus†	disarticulated frontal (dorsal, ventral; unlabeled)	91
Eumeces schneiderii	articulated skull (dorsal, lateral)	57
Eumeces schneiderii	articulated skull (dorsal, ventral; labeled)	89
Eumeces schneiderii	articulated skull (dorsal, ventral; labeled)	92 (modified from 89)
Eumeces schneiderii princeps	articulated skull (dorsal, lateral)	93
Eumeces schneiderii	articulated skull (dorsal; labeled)	68
Eumeces schneiderii	articulated skull (dorsal, ventral; labeled), mandible (lateral, lingual; labeled)	94
Eumeces striatulus†	dentary (lateral, lingual; labeled)	95
Feylinia currori	articulated skull (dorsal, ventral, lateral, posterior; labeled), mandible (lateral, lingual; labeled), disarticulated elements (subset; labeled), histological sections	96
Feylinia currori	articulated skull (dorsal, ventral, lateral; labeled)	2
Feylinia currori	articulated skull (ventral; labeled), histological sections	79
Feylinia currori	articulated skull (dorsal, ventral, lateral; labeled)	123 (modified from 2)
Feylinia elegans	articulated skull (dorsal, lateral; labeled)	2
Feylinia elegans	articulated skull (lateral; labeled)	124 (modified from 2)

Feylinia polylepis	articulated skull (dorsal, ventral; unlabeled)	5
Feylinia polylepis	articulated skull (ventral; labeled)	10
Gongylomorphus bojerii	articulated palatal bones (ventral; labeled)	97
Janetaescincus braueri	articulated palatal bones (ventral; labeled)	97
Janetaescincus braueri	articulated palatal bones (ventral; labeled)	6
Melanoseps ater	articulated palatal bones (ventral; labeled)	97
Melanoseps ater	articulated palatal bones (ventral; labeled)	6
Melanoseps occidentalis	articulated palatal bones (ventral; labeled)	97
Mesoscincus managuae	dentary (labial; unlabeled), postfrontal (dorsal, ventral; unlabeled)	98
*Nessia bipes**	articulated skull (dorsal, lateral; labeled)	99
Nessia layardi	articulated skull (lateral, ventral; labeled)	2
Pamelascincus gardinieri	articulated palatal bones (ventral; labeled)	97
*Plestiodon chinensis**	articulated skull (dorsal, ventral; labeled)	89
*Plestiodon chinensis**	articulated skull (dorsal, ventral; labeled)	92 (modified from 89)
*Plestiodon copei**	anterior dentary (lingual; unlabeled)	100
*Plestiodon elegans**	articulated skull (dorsal, ventral, lateral, unlabeled)	67
*Plestiodon elegans**	articulated skull (ventral; labeled)	17
*Plestiodon fasciatus**	chondrocranium (dorsal, ventral, lateral; labeled), histological sections	101
*Plestiodon fasciatus**	articulated skull (dorsal; labeled)	89
*Plestiodon fasciatus**	mandible (lateral, lingual; labeled)	24
Plestiodon fasciatus	dentary (labial; unlabeled), postfrontal (dorsal, ventral; unlabeled)	98
*Plestiodon gilberti**	articulated skull (dorsal, ventral, lateral; labeled), hyoid (dorsal; labeled)	102
*Plestiodon inexpectatus**	disarticulated frontal (dorsal, ventral; unlabeled)	91
*Plestiodon laticeps**	articulated skull (dorsal, ventral; labeled)	89
*Plestiodon laticeps**	articulated skull (dorsal, ventral; labeled)	92 (modified from 89)
*Plestiodon longirostris**	articulated skull (dorsal, ventral; labeled)	89
*Plestiodon multivirgatus**	articulated skull (dorsal, ventral, lateral; labeled)	103
*Plestiodon obsoletus**	articulated skull (dorsal, ventral; labeled)	89
*Plestiodon obsoletus**	articulated skull (dorsal, ventral; labeled)	92 (modified from 89)
*Plestiodon obsoletus**	articulated skull (dorsal, ventral, lateral; unlabeled), mandible (lateral, lingual; unlabeled)	104
*Plestiodon obsoletus**	articulated skull (dorsal; labeled)	68
*Plestiodon septentrionalis**	articulated skull (dorsal, ventral; labeled)	89
*Plestiodon skiltonianus**	articulated skull (dorsal, ventral; labeled)	89

*Plestiodon skiltonianus**	articulated skull (dorsal, ventral, lateral; labeled), mandible (lateral, lingual, dorsal; labeled)	102
*Plestiodon skiltonianus**	articulated braincase and parietal (dorsal, lateral; labeled)	123
*Plestiodon tetragrammus**	articulated skull (dorsal, ventral; labeled)	89
Proscelotes aenea	articulated palatal bones (ventral; labeled)	97
Proscelotes aenea	articulated palatal bones (ventral; labeled)	6
Proscelotes arnoldi	complete unlabeled skull (ventral)	5
Proscelotes arnoldi	articulated palatal bones (ventral; labeled)	97
Proscelotes eggeli	articulated palatal bones (ventral; labeled)	97
Scelotes arenicola	articulated palatal bones (ventral; labeled)	97
Scelotes mira	articulated palatal bones (ventral; labeled)	97
*Scelotes mossambicus**	articulated palatal bones (ventral; labeled)	97
*Scelotes sexlineatus**	articulated skull and chondrocranium (dorsal, ventral, lateral; labeled); disarticulated elements (subset; labeled); histological sections	105
Scincus hemprichii	ear, quadrate, temporal region (lateral; labeled)	106
Scincus mitranus	ear, quadrate, temporal region (lateral; labeled)	106
Scincus scincus	articulated skull (dorsal, posterior; labeled), disarticulated elements (subset; labeled), histological sections	107
Scincus scincus	ear, quadrate, temporal region (lateral; labeled)	106
Scincus scincus	disarticulated premaxilla (anterior; lateral; labeled)	123
Scincus scincus	articulated skull (dorsal, ventral, lateral; labeled) mandible (lateral; lingual; labeled)	124
Scincus sp.	hyoid (ventral; unlabeled)	108
Scolecoseps boulengeri	articulated palatal bones (ventral; labeled)	97
Sepsina angolensis	articulated palatal bones (ventral; labeled)	97
Sepsina bayoni	articulated palatal bones (ventral; labeled)	97
Sepsina tetradactyla	articulated palatal bones (ventral; labeled)	97
Typhlacontias brevipes	articulated palatal bones (ventral; labeled)	97
Typhlacontias gracilis	articulated palatal bones (ventral; labeled)	97
Typhlacontias ngamiensis	articulated palatal bones (ventral; labeled)	97
Typhlacontias rohani	articulated palatal bones (ventral; unlabeled)	6
Voeltzkowia mira	articulated skull (dorsal, ventral, lateral; labeled), articulated mandible (lateral, lingual, labeled), parietal (ventral; labeled), occipital-sphenoid (dorsal, labeled), nasal capsule (dorsal, labeled), histological sections	109
SPHENOMORPHIDAE		
Anomalopus brevicollis	articulated skull (dorsal, ventral, lateral; unlabeled)	110
Anomalopus gowi	articulated skull (dorsal, ventral, lateral; unlabeled)	110

Anomalopus leuckartii	articulated skull (dorsal, ventral, lateral; unlabeled)	110
Anomalopus pluto	articulated skull (dorsal, ventral, lateral; unlabeled)	110
Anomalopus swansoni	articulated skull (dorsal, ventral, lateral; unlabeled)	110
Anomalopus verreauxii	articulated skull (dorsal, ventral, lateral; unlabeled)	110
Calyptotis ruficauda	articulated skull (dorsal, ventral, lateral; unlabeled)	111
Calyptotis scutirostrum	articulated skull (dorsal, ventral, lateral; unlabeled)	111
Coeranoscincus frontalis	articulated skull (dorsal, ventral, lateral; unlabeled)	110
Coeranoscincus reticulatus	articulated skull (dorsal, ventral, lateral; unlabeled)	110
Coeranoscincus reticulatus	mandible (lingual; unlabeled)	19
Coggeria naufragus	hyoid (ventral; labeled), articulated palatal bones (ventral; labeled)	112
*Ctenotus australis**	articulated skull (ventral; unlabeled)	57
Ctenotus leae	articulated skull (dorsal, ventral; labeled)	19
Ctenotus inornatus	articulated skull (ventral; labeled)	17
*Eulamprus quoyii**	articulated skull (dorsal, ventral, lateral; labeled), mandible (lateral, lingual; labeled)	57
*Eulamprus quoyii**	articulated skull (dorsal, ventral, lateral, posterior; labeled), mandible (lateral; labeled), hypobranchial apparatus (ventral; labeled)	113
Eulamprus quoyii	articulated skull (lateral; unlabeled)	17
Eulamprus quoyii	dentary (lateral, lingual; unlabeled), frontal (dorsal, ventral; unlabeled)	23
Eremiascincus douglasi	mandible (lingual; unlabeled)	25
Eremiascincus fasciolatus	articulated skull (dorsal, ventral; labeled)	114
*Eremiascincus pardalis**	articulated palatal bones (ventral; labeled)	49
*Eremiascincus pardalis**	articulated skull (ventral; unlabeled)	17
Eremiascincus richardsonii	articulated skull (dorsal, ventral, lateral, posterior; labeled), disarticulated elements (all elements; labeled)	115
Glaphyromorphus mjobergi	articulated skull (ventral; unlabeled)	17
*Glaphyromorphus mjobergi**	mandible (lateral, lingual; unlabeled)	24
Isopachys anguinoides	articulated skull (dorsal, ventral, lateral; unlabeled)	78
Isopachys borealis	articulated skull (dorsal, ventral, lateral; unlabeled, hyoid (ventral; unlabeled)	116
Isopachys gyldenstolpei	articulated skull (dorsal, ventral, lateral; unlabeled)	78
Isopachys roulei	articulated skull (dorsal, ventral, lateral; unlabeled)	78
Kaestlea bilineata	articulated palatal bones (ventral; unlabeled)	117
*Larutia larutensis**	articulated skull (ventral; unlabeled)	59

Lerista bougainvilli	articulated palatal bones (ventral; labeled)	49
Lerista elegans	articulated palatal bones (ventral; labeled)	49
Lerista elegans	articulated skull (ventral; unlabeled)	37
Lerista elegans	articulated skull (ventral; unlabeled)	38
Lipinia semperi	articulated palatal bones (ventral; unlabeled)	36
Nangura spinosa	articulated skull (dorsal, ventral, lateral; unlabeled), mandible (lateral, lingual; unlabeled)	118
*Notoscincus ornatus**	articulated skull (ventral; unlabeled)	37
*Notoscincus ornatus**	articulated skull (ventral; unlabeled)	38
Ophioscincus cooloolensis	articulated skull (dorsal, ventral, lateral; unlabeled)	110
Ophioscincus ophioscincus	articulated skull (dorsal, ventral, lateral; unlabeled)	110
Ophioscincus truncatus	articulated skull (dorsal, ventral, lateral; unlabeled)	110
Prasinohaema virens	articulated palatal bones (ventral; unlabeled)	36
Saiphos equalis	articulated skull (dorsal, ventral, lateral; unlabeled)	111
Scincella darevski	articulated skull (lateral; labeled)	119
Scincella formosensis	mandible (lingual; unlabeled)	36
Scincella huanrenensis	articulated skull (dorsal; unlabeled)	120
Scincella lateralis	articulated palatal bones (ventral; unlabeled)	36
Scincella reevesii	articulated skull (dorsal; unlabeled)	120
Scincella lateralis	dentition	121
*Silvascincus murrayi**	articulated skull (ventral; labeled)	17
*Silvascincus murrayi**	mandible (lingual; unlabeled)	19
Sphenomorphus derooyae	articulated skull (ventral; unlabeled)	122
*Sphenomorphus indicus**	snout (lateral; unlabeled)	30
*Sphenomorphus indicus**	articulated skull (lateral, posterior; labeled)	66
Sphenomorphus jabiensis	articulated skull (dorsal, ventral; unlabeled)	5
*Sphenomorphus muelleri**	snout (lateral; unlabeled)	30
Sphenomorphus pardalis	articulated skull (ventral; unlabeled)	122
Sphenomorphus solomonis	articulated palatal bones (ventral; labeled)	58
Sphenomorphus sp.†	mandible (lateral, lingual; unlabeled)	24
Tropidophorus beccari	articulated palatal bones (ventral; labeled)	58
Tropidophorus misaminius	articulated skull (dorsal, ventral, lateral, posterior; labeled), mandible (lateral, lingual; labeled), detail of braincase (lateral; labeled)	123

References

1. Van den Heever JA. The cranial and cervical muscles of the South African limbless lizard *Typhlosaurus aurentiacus aurantiacus* Peters (Reptilia: Sauria). Ann S Afr Mus. 1976; 69: 169-214.
2. Rieppel O. The skull and jaw adductor musculature in some burrowing scincomorph lizards of the genera *Acontias*, *Typhlosaurus* and *Feylinia*. J Zool, Lond. 1981;195: 493-528.
3. Rieppel O. The phylogenetic relationship of the genus *Acontophiops* Sternfeld (Sauria: Scincidae), with a note on mosaic evolution. Ann Transvaal Mus. 1982;33: 241-257.
4. Rieppel O. The upper temporal arcade of lizards: an ontogenetic problem. Rev Suisse Zool. 1994;91: 475-482.
5. Greer AE. A subfamilial classification of scincid lizards. Bull Mus Comp Zool Harvard Univ. 1970;139: 151-185.
6. Caputo, V. The secondary palate in squamates: structure and functional hypotheses. In: Lanzavecchia G, Valvassori R, editors. Form and Function in Zoology. Selected Symposi and Monographs U.Z.I. 5. Modena: Mucchi; 1991. pp. 307-324.
7. De Villiers CGS. Über den Schädel des südafrikanischen schlangenartigen Scinciden *Acontias meleagris*. Anat Anz. 1939;88: 320-347.
8. Brock GT. The skull of *Acontias meleagris*, with a study of the affinities between lizards and snakes. Zool J Linn Soc London. 1941;41: 71-88.
9. Van der Merwe NJ. Die Skedelmorfologie van *Acontias meleagris* (Linn.). Tydskr Wetenskap Kuns. 1944;5: 59-88.
10. Rieppel O, Gauthier J, Maisano J. Comparative morphology of the dermal palate in squamate reptiles, with comments on phylogenetic implications. Zool J Linn Soc. 2008;152: 131-152.
11. Vanhooydonck B, Boistel R, Fernandez V, Herrel A. Push and bite: trade-offs between burrowing and biting in a burrowing skink (*Acontias percivali*). Biol J Linn Soc. 2011;102: 91-99.
12. Hutchinson MN, Scanlon J. New and unusual Plio-Pleistocene lizard (Reptilia: Scincidae) from Wellington Caves, New South Wales, Australia. J Herpetol. 2009;43: 139-147.
13. Hollenshead MG, Mead JI, Swift SL. Late Pleistocene *Egernia* group skinks (Squamata: Scincidae) from Devil's Lair, Western Australia. Alcheringa 2010;35: 31-51.
14. Hutchinson MN. A skeletal specimnen of the giant skink *Macroscincus coctei* in the American Museum of Natural History. Copeia 1989:492-494.
15. Shea GM. The genera *Tiliqua* and *Cyclodomorphus* (Lacertilia: Scincidae): generic diagnoses and systematic relationships. Mem Queensl Mus. 1990;29: 495-519.
16. Mitchell FJ. The scincid genera *Egernia* and *Tiliqua* (Lacertilia). Rec South Aust Mus. 1950;9: 275-308.
17. Greer AE. The Biology and Evolution of Australian Lizards. Chipping Norton: Surrey Beatty and Sons Pty Ltd; 1989.
18. Peracca MG. 1895. Sul fatto di due distinte dentizioni nella Tiliqua scincoides White. Bollettino dei Musei di Zoologia ed Anatomia Comparata della R. Università di Torino 1895;10(217): 1-3, 1 plate. [Reprinted on pp. 269-272 of The Life and Herpetological

Contributions of Mario Giacinto Peracca (1861-1923). Society for the Study of Amphibians and Reptiles, in Cooperation with Societas Herpetologica Italica, Villanova, Pennsylvania].
19. Hutchinson MN. Family Scincidae. In: Glasby CJ, Ross GJB, Beesley PL, editors. Fauna of Australia, vol 2A, Amphibia and Reptilia. Australian Government Publishing Service, Canberra; 1993. pp. 261-279.
20. Arena A, Archer M, Godthelp H, Hand SJ, Hocknull S. Hammer-toothed 'marsupial skinks' from the Australian Cenozoic. Proc R Soc B. 2011; doi:10.1098/rsb.2011.0486.
21. Hutchinson MN. The systematic relationships of the genera *Egernia* and *Tiliqua* (Lacertilia: Scincidae). A review and immunological reassessment. In: Banks CB, Martin AA, editors. Proceedings of the Melbourne Herpetological Symposium. Melbourne: Zoological Board of Victoria; 1981. pp. 176-193.
22. Hollenshead MG. Geometric morphometric analysis of cranial variation in the *Egernia depressa* (Reptilia: Squamata: Scincidae) species complex. Rec West Aust Mus. 2011;26: 138-153.
23. Mackness BS, Hutchinson MN. Fossil lizards from the Early Pliocene Bluff Downs local fauna. T Roy Soc South Aust. 2000;124: 17-30.
24. Hutchinson MN. Origins of the Australian scincid lizards: a preliminary report on the skinks of Riversleigh. Beagle 1992;9: 61-69.
25. Greer AE. A phylogenetic subdivision of Australian skinks. Rec Australian Mus. 1979;32: 339-371.
26. Hugi J, Mitgutsch C, Sánchez-Villagra MR. Chondrogenic and ossification patterns and sequences in White's skink *Liopholis whitii* (Scincidae, Reptilia). Zoosyst. Evol. 2010;86 :21-32.
27. Martin JE, Hutchinson MN, Meredith R, Case JA, Pledge NS. The oldest genus of scincid lizard (Squamata) from the Tertiary Etadunna Formation of South Australia. J Herpetol. 2004;38: 180-187.
28. Hutchinson MN, Milne T, Croft T. Redescription and ecological notes on the pygmy bluetongue, *Tiliqua adelaidensis* (Squamata: Scincidae). Trans Roy Soc South Aust. 1994;118: 217-226.
29. Hutchinson MN, Mackness BS. Fossil lizards from the Pliocene Chinchilla Local Fauna, Queensland, with a description of a new species. Rec South Aust Mus. 2002;35: 169-184.
30. Smith, MA. A review of the genus Lygosoma (Scincidae: Reptilia) and its allies. Rec Indian Mus. 1937;39 :213-234.
31. Smith M. Reptiles from late Pleistocene deposits on Kangaroo Island, South Australia. T Roy Soc South Aust. 1982;106: 61-66.
32. Brühl, CB. Zootomie aller Thierklassen für Lernende, nach Autopsien, skizziert. Lief. 38. Vienna: Alfred Holder; 1886.
33. Wineski LE, Gans C. Morphological basis of the feeding mechanics in the Shingle-back lizard *Trachydosaurus rugosus* (Scincidae, Reptilia). J Morphol. 1984;181: 271-295.
34. De Vree F, Gans C. Kinetic movements in the skull of adult *Trachydosaurus rugosus*. ZBL VET MED C. 1987;16: 206-209.
35. Darevsky IS, Tschumakov IS. A new Pleistocene species of the lizard *Ablepharus* from Rudny Altai. Paleontol Zh. [in Russian]. 1962;1962: 127-30.

36. Greer AE. The generic relationships of the scincid lizard genus *Leiolopisma* and its relatives. Australian J Zool suppl ser. 1974;31: 1-67.
37. Fuhn IE. Revision and redefinition of the genus *Ablepharis* Lichtenstein, 1823 (Reptilia, Scincidae). Rev Roumaine Biol (ser. Zool). 1969;14: 23-41.
38. Fuhn IE. The "polyphyletic" origin of the genus *Ablepharus* (Reptilia, Scincidae): a case of parallel evolution. J Zoolog Syst Evol Res. 1969;7: 67-76.
39. Gruber U. *Ablepharus kitaibelii* Bibron & Bory 1833 - Johannisechse. In: Böhme W, editor. Handbuch der Reptilien und Amphibien Europas, Band I., Echsen I. Wiesbaden: Akademische Verlagsgesellschaft; 1981. pp. 292-307.
40. Haas G. Zum Bau des Primordialcraniums und des Kopfskelettes von *Ablepharus pannonicus*. Acta Zool Stockholm. 1935;16: 409-429.
41. Greer AE, Parker F. *Geomyersia glabra*, a new genus and species of scincid lizard from Bouganville, Solomon Islands, with comments on the relationships of some lygosomine genera. Breviora. 1968;302: 1-17.
42. Pregill GK. Fossil lizards from the late Quaternary of 'Eua, Tonga. Pac Sci. 1993;47: 101-114.
43. Daza JD, Bauer AM, Sand C, Lilley I, Wake TA, Valentin F. Reptile remains from Tiga (Tokanod), Loyalty Islands, New Caledonia. Pacific Science. 2015;69: 531-557.
44. Kiester AR. A new forest skink from Ponape. Breviora 1982;468: 1-10.
45. Fuhn IE. Revision du phylum forestier du genre *Panaspis* Cope (Reptilia, Scincidae, Lygosominae). Rev Roumaine Biol (ser. Zool). 1972;17: 257-271.
46. Estes R. Handbuch der Paläoherpetologie. Sauria terrestria, Amphisbaenia. Stuttgart: Fischer; 1983.
47. Greer AE. The generic relationships of the African scincid genus *Eumecia*. Breviora. 1967;276: 1-9.
48. Zweifel RG. A new scincid lizard of the genus *Leiolopisma* from New Guinea. Zool Meded. 1972;47: 530-539.
49. Greer AE. A new generic arrangement for some Australian scincid lizard. Breviora. 1967;267: 1-19
50. Hutchinson MN, Robertson P, Rawlinson PA. Redescription and ecology of the endemic Tasmanian scincid lizards *Leiolopisma microlepidotum* and *L. pretiosum*. Pap Proc Roy Soc Tasmania. 1989;123: 257-274.
51. Worthy TH. Osteological observations on the larger species of the skink *Cyclodina* and the subfossil occurrence of these and the gecko *Hoplodactylus duvaucelii* in the North Island, New Zealand. New Zealand J Zool. 1987;14: 219-229.
52. Worthy TH. Fossil skink bones from Northland, New Zealand, and description of a new species of *Cyclodina*, Scincidae. J R Soc N Z. 1991;21: 329-348.
53. Gill BJ. Subfossil bones of a large skink (Reptilia: Lacertilia) from Motutapu Island, New Zealand. Rec Auckland Inst Mus. 1985;22: 69-76.
54. Lee MS, Hutchinson MN, Worthy TH, Archer M, Tennyson AJD, Worthy JP, Scofield RP. Miocene skinks and geckos reveal long-term conservatism of New Zealand's lizard fauna. Biol Lett .2009;5: 833-837.
55. Fuhn IE. Contribution à la systématique des lygosomines africains (Reptilia, Scincidae). 1. Les espèces attribuées au genre *Ablepharis*. Rev Roumaine Biol (ser. Zool). 1970;15: 379-393.
56. Hutchinson MN, Donnellan SC. A new species of scincid lizard related to *Leiolopisma*

entrecasteauxii, from southeastern Australia. Trans R S Aust. 1988;112: 143-151.
57. Siebenrock R. Zur Kenntnis des Kopfskelettes der Scincoiden, Anguiden and Gerrhosauriden. Ann Naturhist Hofmus Wien. 1892;7: 163-195.
58. Greer AE. The relationships of the skinks referred to the genus *Dasia*. Breviora. 1970;348: 1-30.
59. Greer AE. The systematics and evolutionary relationships of the scincid lizard genus *Lygosoma.* J Nat Hist. 1977;11: 515-540.
60. Greer AE. On the evolution of the giant Cape Verde scincid lizard *Macroscincus coctei*. J Nat Hist. 1976;10: 691-712.
61. Pearson H. The skull and some related structures of a late embryo of *Lygosoma*. J Anat. 1921;56: 20-44.
62. Cuvier G. Recherches sur les Ossements Fossiles où l'on Rétablit les Caractères de Plusieurs Animaux dont les Révolutions du Globe ont Détruit les Espèces, nouvelle édition., Vol. 5. Paris: Dufour & D'Ocagne; 1824.
63. Orlandi S. Note anatomische sul *Macroscincus coctei* (Barb. D. Boc.). Atti Soc Linguistica. 1894;5: 175-204.
64. Rao MKM, Ramaswami LS. The fully formed chondrocranium of *Mabuya* with an account of the adult osteocranium. Acta Zool. 1952;33: 209-275.
65. Prasad J. The bony palate in skull of *Mabuya dissimilis* Hallowell. Sci Cult. 1954;20: 49-51.
66. Prasad J. Studies on the temporal region in the skull of skinks, *Lygosoma indicum indicum* (Gray) and *Mabuya dissimilis* (Hallowell). Agra Univ J Res Sci. 1957;5: 321-325.
67. Greer AE. Lygosomine (Scincidae) monophyly: a third, corroborating character and a reply to critics. J Herpetol. 1986;20: 123-126.
68. Griffith H, Ngo A, Murphy RW. A cladistics evaluation of the cosmopolitan genus *Eumeces* Wiegmann (Reptilia, Squamata, Scincidae). Russian J Herpetol. 2000;7: 1-16.
69. Rastegar-Pouyani NR, Fattahi R, Gholamifard A. cranial osteology of the bridled skink, *Trachylepis vittata* (Olivier, 1804), and its comparison with *Trachylepis aurata transcaucasica* (Chernov, 1926). Russ J Herpetol. 2013;20: 276-286.
70. Mohammed MBH. The development and growth of the skull of *Mabuya aurata* (Scincidae, Reptilia). J Egyptian German Soc Zool. 1991;3: 25-40.
71. Faizi H, Rastegar-Pouyani NR. Further studies on the lizard cranial osteology, based on a comparative study of the skull in *Trachylepis aurata transaucasica* and *Laudakia nupta* (Squamata: Sauria). Russian J Herpetol. 2007;14: 107-116.
72. Jerez A. Structural characteristics of the skeleton in *Mabuya sp.* (Squamata: Scincidae): a comparison with African scincids. Actualidades Biológicas. 2012;34: 207-223.
73. Jerez A, Sánchez-Martínes PM, Guerra-Fuentes RA. Embryonic skull development in the Neotropical viviparous skink *Mabuya* (Squamata: Scincidae). Acta Zoológica Mexicana. 2015;31: 391-402.
74. Travassos H. Estudo da variação de *Mabuya punctata* (Gray, 1939) (Ordem Squamata Oppel, 1811 — Fam. Scincidae Gray, 1825). Bol Mus Nac Brasil, Zool n s. 1946;60: 1-56, 11 pls.
75. Wever EG. The function of the middle ear in lizards: *Eumeces* and *Mabuya* (Scincidae). J Exp Zool. 1973;183: 225-240.

76. Skinner MM. Ontogeny and adult morphology of the skull of the South African skink, *Mabuya capensis* (Gray). Ann Univ Stellenbosch. 1973;48: 1-116.
77. Louryan S. Développement des ébauches squelettiques du complexe mandibulo-otique chez *Mabuia megalura* (Lacertilia: Scincidae). Ann soc R zool Belg. 1989;119: 47-57.
78. Heyer WR. A new limbless skink (Reptilia: Scincidae) from Thailand with comments on the generic status of the limbless skinks of Southeast Asia. Fieldiana Zool. 1972;58: 109-129.
79. Caputo V. The cranial osteology and dentition in the scincid lizards of the genus *Chalcides* (Reptilia, Scincidae). In: Picariello O, Odierna G, Guarino FM, editors. Proceedings of the Fourth National Congress of Societas Herpetologica Italica (SHI) (Ercolano, Naples, Italy, 18-22 June, 2002). Ital J Zool. 2004;71(Supplement 2): 35-45.
80. Haas G. Über das kopfskelett von *Chalcides guentheri* (*Seps monodactylus*). Acta Zool Stockholm. 1936;17: 55-74.
81. Romer AS. Osteology of the Reptiles. Chicago: University of Chicago Press; 1956.
82. El-Toubi MR, Kamal AM. The development of the skull of *Chalcides ocellatus*. II. The fully formed chondrocranium and the osteocranium of a late embryo. J Morphol. 1959;105: 55-104.
83. Kamal AM. The cranial osteology of the adult *Chalcides ocellatus*. Anat Anz. 1965;117: 338-370.
84. Schneider B. *Chalcides ocellatus* (Forskål 1775) – Walzenskink. In: Böhme W, editor. Handbuch der Reptilien und Amphibien Europas, Band 1: Echsen (Sauria) (Gekkonidae, Agamidae, Chamaeleonidae, Anguidae, Amphisbaenidae, Scincidae, Lacertidae). Wiesbaden: Akademische Verlagsgesellschaft; 1981. pp. 338-354.
85. Mohammed MBH. Sequence of ossification in the skeleton of growing lizard *Chalcides ocellatus* Forscal (Scincidae, Reptilia). Qatar Univ Sci Bull. 1988;8: 117-136.
86. Mermer A. Anadolu'da Yasayan *Chalcides ocellatus* 'un –(Sauria: Scincidae) Kafatasi osteolojisi. Turk J Zool. 1996;20: 259-264. [The Cranial osteology of *Chalcides ocellatus* (Sauria: Scincidae) in Anatolia].
87. López-Jurado LF. *Chalcides simonyi* Steindacher, 1891 – Purpurarien-Skink. In: Böhme W, editor. Handbuch der Reptilien und Amphibien Europas Band 6: Die Reptilien der Kanarischen Inseln, der Selvagens-Inseln unde des Madeira-Archipels. Wiesbaden: Akademische Verlagsgesellschaft; 1998. pp. 189-199.
88. Holman, JA. Herpetofauna of the WaKeeney local fauna (Lower Pliocene: Clarendonian) of Trego County, Kansas. Papers on Paleontology, U Mich. 1975;12: 49-66.
89. Kingman RH. A comparative study of the skull in the genus *Eumeces* of the family Scincidae. Univ Kansas Sci Bull. 1932;20: 273-295.
90. Holman, JA. A new *Peltosaurus* (Reptilia, Sauria, Anguidae) from the Upper Miocene of Nebraska. J Herpetol. 1976; 10: 41-44.
91. Wellstead CF. Taxonomic re-assignment of the Miocene lizard, *Peltosaurus minimus*, from Nebraska. Copeia. 1982;1982: 549-553.
92. Taylor EH. A taxonomic study of the cosmopolitan scincoid lizards of the genus *Eumeces* with an account of the distribution and relationships of its species. Univ Kansas Sci Bull. 1935;36: 1-643.
93. Darevsky IS. *Eumeces schneiderii* (Daudin 1802) – Tüpfelskink. In: Böhme W, editor. Handbuch der Reptilien und Amphibien Europas, Band 1: Echsen (Sauria)

(Gekkonidae, Agamidae, Chamaeleonidae, Anguidae, Amphisbaenidae, Scincidae, Lacertidae). Wiesbaden: Akademische Verlagsgesellschaft; 1981. pp. 355-365.
94. Yildirim E, Kumlutaş Y, Ilgaz Ç. A preliminary study of comparative osteology of the scincid lizard *Eumesces schneideri* (Daudin, 1802) (Squamata: Scincidae) subspecies in Anatolia. Anadolu Doğa Bilimleri Dergisi. 2015;61: 34-43.
95. Taylor EH. Extinct lizards from the upper Pliocene deposits of Kansas. Kans State Geol Surv Bull. 1941;38: 165-176.
96. Propach M. Untersuchungen zur Osteologie des Schädels von *Feylinia currori* Gray (1845) und ihre systematiche Eingliederung innerhalb der Sauria. Ph.D. Dissertation, Ludwig-Maximilians- Universität. 1968.
97. Greer AE. The systematics and evolution of the subsaharan African, Seychelles and Mauritius scincine scincid lizards. Bull Mus Comp Zool Harvard Univ. 1970;140: 1-24.
98. Covacevich JA, Couper PJ, James C. A new skink, *Nangura spinosa* gen. et sp. nov., from a dry rainforest of southeastern Queensland. Mem Queensland Mus. 1993;34: 159-167.
99. Deraniyagala PEP. A Colored Atlas of some Vertebrates from Ceylon. Colombo: The Ceylon Government Press; 1953.
100. Olson RE, Marx B, Rome R. Descriptive dentition morphology of lizards of Middle and North America, I. Scincidae, Teiidae, and Helodermatidae. Bull Maryland Herpetol Soc. 1986;22: 97-124.
101. Rice EL. The development of the skull in the skink, *Eumeces quinquelineatus* L. 1. The chondrocranium. J Morphol. 1920;34: 119-243.
102. Nash DF, Tanner WW. A comparative study of the head and thoracic osteology and myology of the skinks *Eumeces gilberti* Van Denburgh and *Eumeces skiltonianus* (Baird and Girad). Brigham Young Univ Sci Bull, Biol Ser. 1970;12: 1-32.
103. Hikida T. Postembryonic development of the skull of the Japanese skink, *Eumeces latiscutatus* (Scincidae). Japenese J Herpetol. 1978;7: 56-72.
104. Estes R, de Queiroz K, Gauthier JA. Phylogenetic relationships within Squamata. In: Estes R, Pregill G, editors. Phylogenetic Relationships of the Lizard Families. Essays Commemorating Charles L Camp. Stanford: Stanford University Press; 1988. pp. 119-281.
105. Leonard CJ. The cranial morphology of the didactyle sand skink *Scelotes bipes sexlineatus* (Harlan). Pub Univ Western Cape, ser B. 1978;1: 1-88.
106. Arnold EN, Leviton AE. A revision of the lizard genus *Scincus* (Reptilia: Scincidae). Bull Br Mus (N.H.) Zool. 1977;31: 187-248.
107. El-Toubi MR. The osteology of the lizard *Scincus scincus* (Linn). Bull Fac Sci Cairo Univ. 1938;14: 1-38.
108. Corsy F. Evolution de l'Appareil Hyo-branchial. Marseille: P. Ciarfa; 1933.
109. Rabanus K. Über das Skelett von *Voeltzkowia mira* Bttgr. Ein Beitrag zur Osteologie der Eidechsen. In: Voeltzkow, A, editor. Reise in Ostafrika in den Jahren 1903–1905 Wissenschaftliche Ergebnisse. Band IV (3). Anatomie und Entwickelungsgeschichte; 1911. pp. 279-330, pls. 20–22.
110. Greer AE, Cogger HG. Systematics of the reduce-limbed and limbless skinks currently assigned to the genus *Anomolopus* (Lacertilia: Scincidae). Rec Australian Mus. 1985;37: 11-54.

111. Greer AE. The Australian scincid lizard genus *Calyptotis* de Vis: resurrection of the name, description of four new species, and discussion of relationships. Rec Australian Mus. 1983;35: 29-59.
112. Couper PJ, Covacevich JA, Marsterson SP, Shea GM. *Coggeria naufragus* gen. et sp. nov., a sand-swimming skink from Fraser Island, Queensland. Mem Queensl Mus. 1996;39: 233-241.
113. King D. The osteology of the water skink, *Lygosoma* (*Sphenomorphus*) *quoyii*. Aust J Zool. 1964;12: 201-216.
114. Greer AE. *Eremiascincus*, a new generic name for some Australian sand swimming skinks (Lacertilia: Scincidae). Rec Australian Mus 1979;32: 321-338.
115. Gelnaw WB. On the cranial osteology of *Eremiascincus* and its use for identification. M.S. Thesis, East Tennessee State University. 2011. Available: http://dc.etsu.edu/cgi/viewcontent.cgi?article=2485&context=etd
116. Lang M, Böhme W. Description and phylogenetic position of a new species of *Isopachys* from central Thailand and southern Burma (Squamata: Scincidae). Bull Inst R Sci Nat Belg, Biol. 1990;60: 231-240.
117. Eremchenko VK, Das, I. 2004. *Kaestlea*: A new genus of scincid lizards (Scincidae: Lygosominae) from the western Ghats, south-western India. Hamadryad 2004;28: 43-50.
118. Chovanec K. Non-anguimorph Lizards of the Late Oligocene and Early Miocene of Florida and Implications for the Reorganization of the North American Herpetofauna. M.S. Thesis, East Tennessee State University. 2014.
119. Nguyen QN, Ananjeva NB, Orlov NL, Rybaltovsky E, Böhme W. A new species of the genus *Scincella* Mittelmann, 1950 (Squamata: Scincidae) from Vietnam. Russ J Herpetol. 2010;17: 269-274.
120. Wang ZY, Zhao EM. Studies on Chinese species of *Scinella* (Scincidae, Sauria). Acta Herpetol Sinica. 1986;5: 267-277.
121. Townsend VR Jr, Akin JA, Felgenhauer BE, Dauphine J, Kidder SA. Dentition of the ground skink, *Scincella lateralis* (Sauria, Scincidae). Copeia. 1999: 783-788.
122. Greer AE, Parker F. The *fasciatus* species group of *Sphenomorphus* (Lacertilia: Scincidae): notes on eight previously described species and descriptions of three new species. Proc Sci Soc Papua New Guinea. 1974;25: 31-61.
123. Evans SE. The skull of lizards and tuatara. In: Gans C, Gaunt AS, Adler K, editors. Biology of the Reptilia, Volume 20 (The Skull of Lepidosauria). Ithaca: Society for the Study of Amphibians and Reptiles; 2008. pp. 1-347.
124. Conrad JL. Phylogeny and systematics of Squamata (Reptilia) based on morphology. Bull Am Mus Nat Hist. 2008;310: 1-182.
