# Supplementary figures and images for: Comparative skull anatomy of terrestrial and crevice-dwelling Trachylepis skinks (Squamata: Scincidae) with a survey of resources in scincid cranial osteology
Source: PLoS One. 2017 Sep 13;12(9):e0184414. doi: 10.1371/journal.pone.0184414 (PMC5597209; doi:10.1371/journal.pone.0184414)

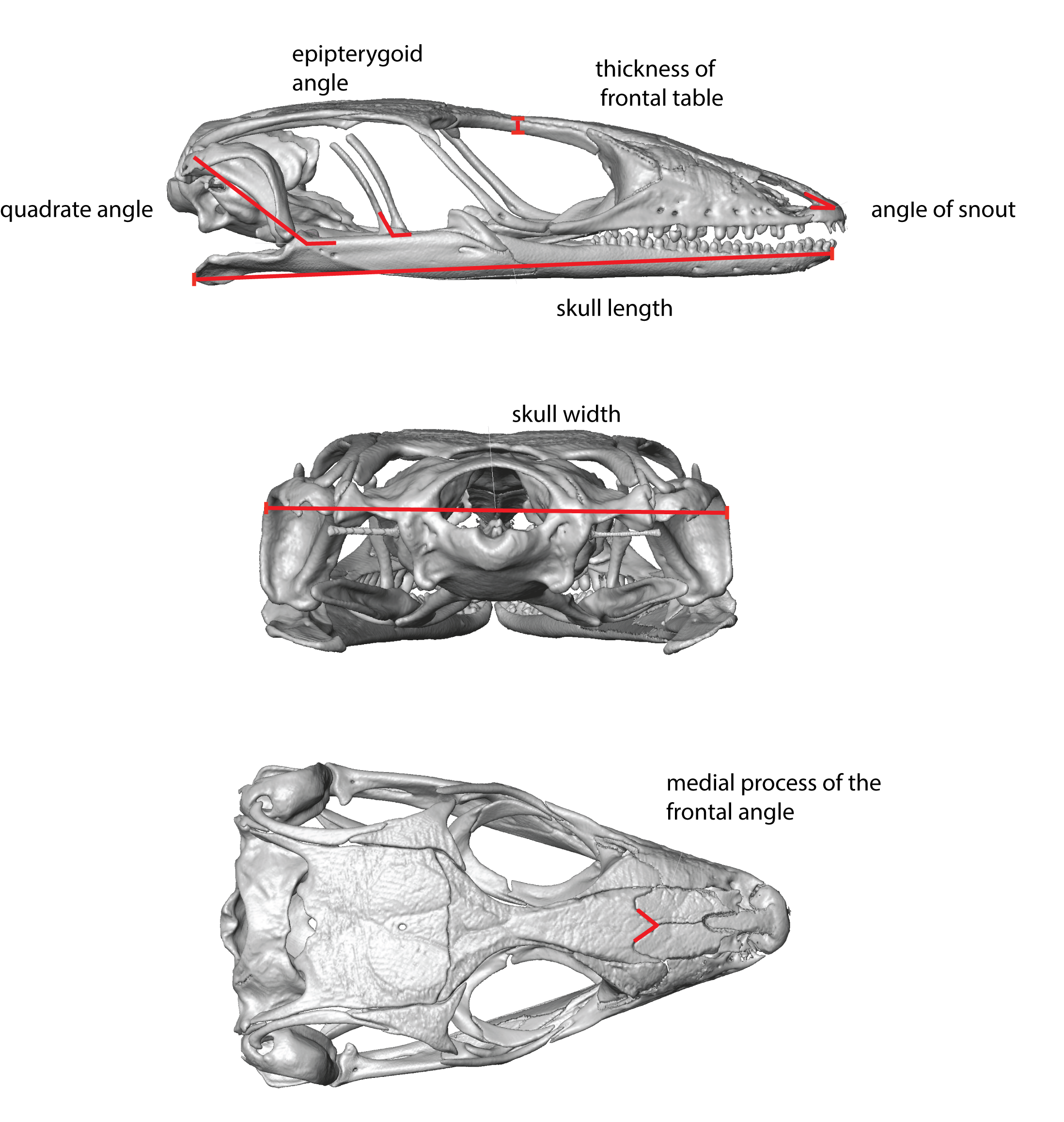

Supplement: S1 Fig — These measurements include skull length, skull width, thickness of the frontal table, angle of the snout, quadrate angle, epipterygoid angle, and angle of the medial process of the frontal. (TIF) [file pone.0184414.s002.tif]

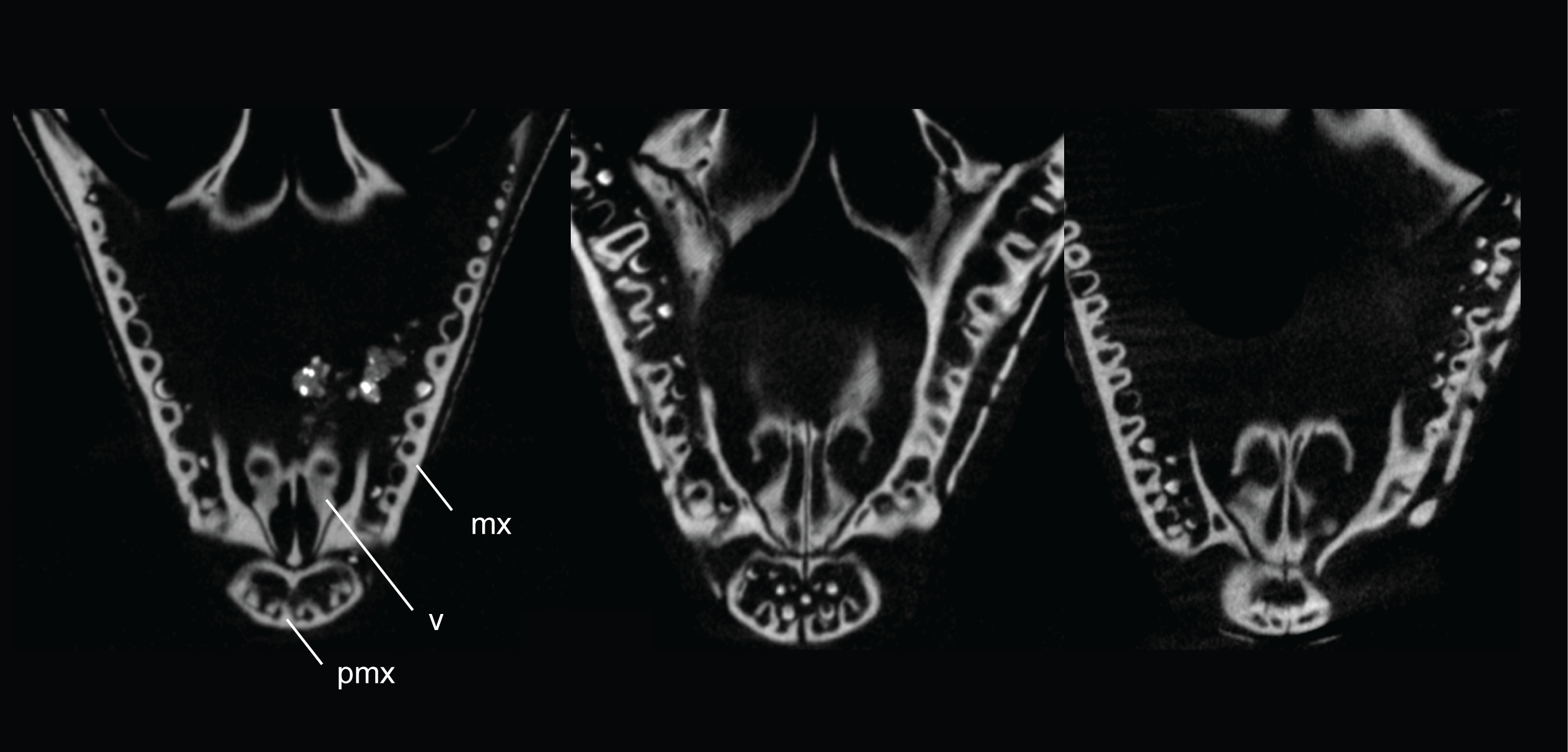

Supplement: S2 Fig — The premaxilla is fused in T. laevis, while midline sutures are visible in T. sulcata and T. gonwouoi. Abbreviations: mx, maxilla; pmx, premaxilla; v, vomer. (TIF) [file pone.0184414.s003.tif]

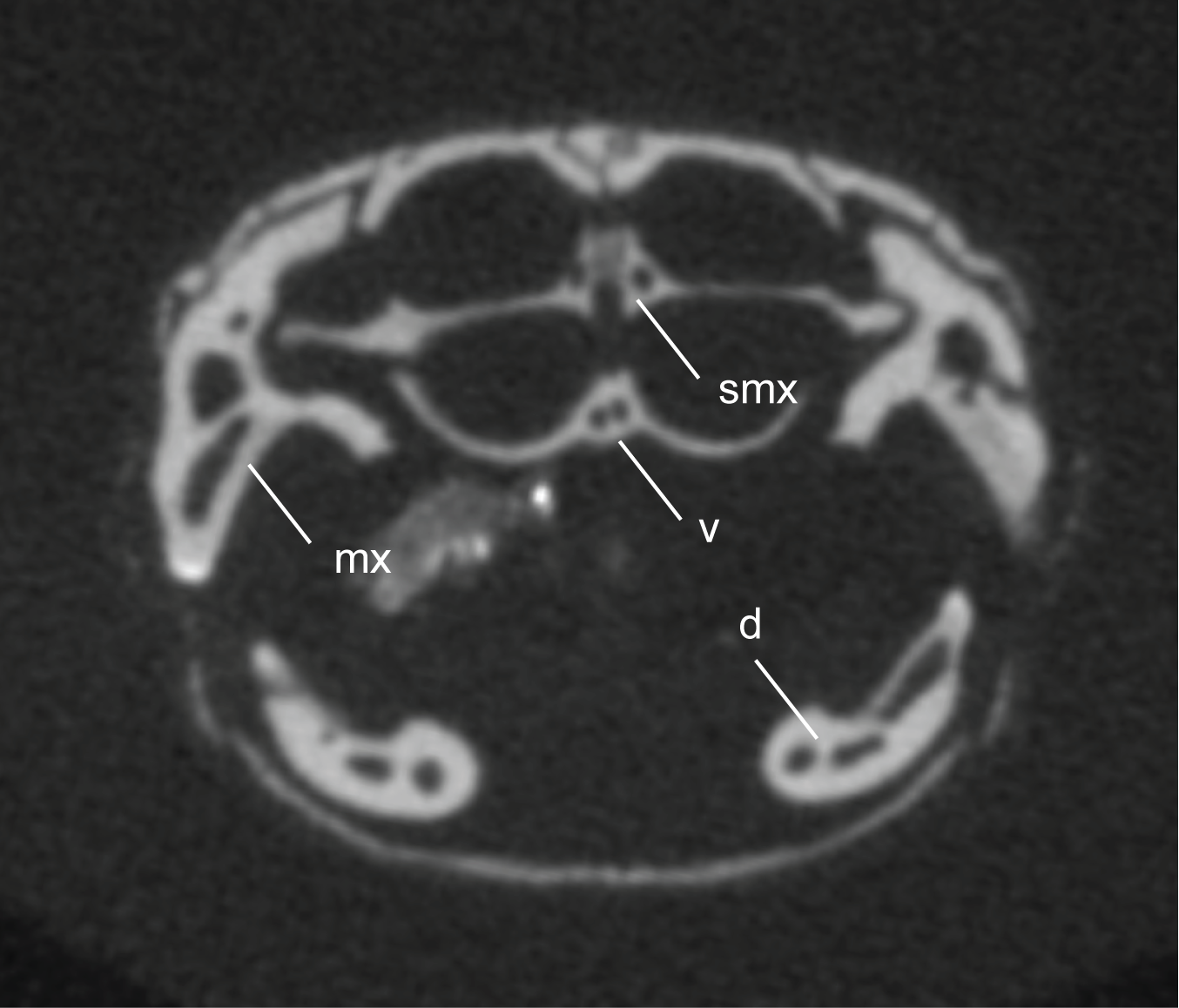

Supplement: S3 Fig — The septomaxilla is fused at the midline. Abbreviations: d, dentary; mx, maxilla; smx, septomaxilla; v, vomer. (TIF) [file pone.0184414.s004.tif]

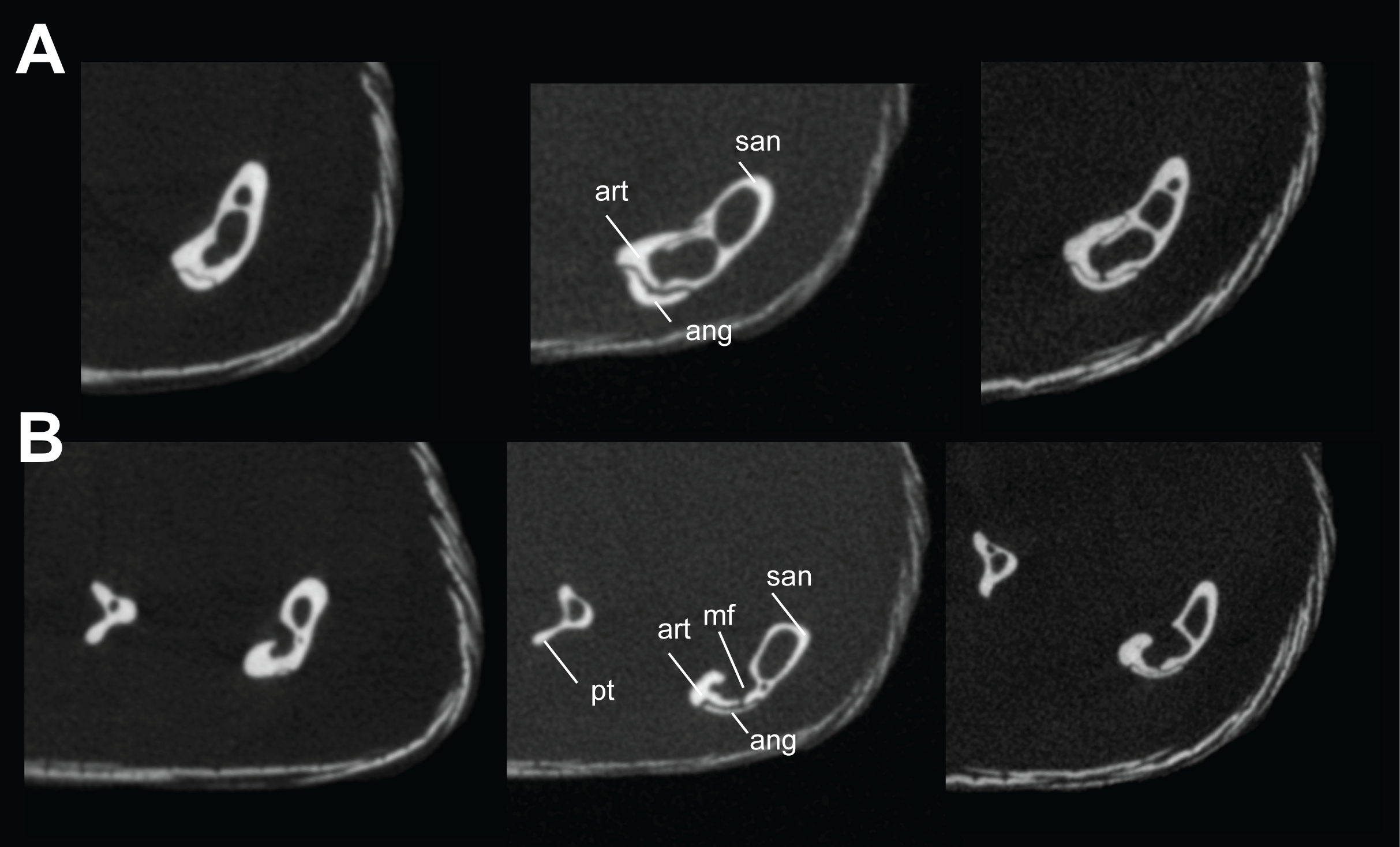

Supplement: S4 Fig — The articular and surangular are fused in T. laevis, while they are separate in T. sulcata and T. gonwouoi. Abbreviations: ang, angular; art, articular; mf, mandibular fossa; pt, pterygoid; san, surangular. (TIF) [file pone.0184414.s005.tif]
